# Tankyrase inhibition sensitizes melanoma to PD-1 immune checkpoint blockade in syngeneic mouse models

**DOI:** 10.1038/s42003-020-0916-2

**Published:** 2020-04-24

**Authors:** Jo Waaler, Line Mygland, Anders Tveita, Martin Frank Strand, Nina Therese Solberg, Petter Angell Olsen, Aleksandra Aizenshtadt, Marte Fauskanger, Kaja Lund, Shoshy Alam Brinch, Max Lycke, Elisabeth Dybing, Vegard Nygaard, Sigurd Læines Bøe, Karen-Marie Heintz, Eivind Hovig, Clara Hammarström, Alexandre Corthay, Stefan Krauss

**Affiliations:** 1 Department of Immunology and Transfusion Medicine, Oslo University Hospital, P.O. Box 4950, Nydalen 0424 Oslo, Norway; 2 Hybrid Technology Hub - Centre of Excellence, Institute of Basic Medical Sciences, University of Oslo, P.O. Box 1110, Blindern 0317 Oslo, Norway; 3 K. G. Jebsen Centre for B cell malignancies, Oslo University Hospital, P.O. Box 4950, Nydalen 0424 Oslo, Norway; 4 School of Health Sciences, Kristiania University College, P.O. Box 1190, Sentrum 0107 Oslo, Norway; 5 Department of Tumor Biology, Institute for Cancer Research, Oslo University Hospital, P.O. Box 4953, Nydalen 0424 Oslo, Norway; 6 Department of Medical Biochemistry, Oslo University Hospital, Radiumhospitalet, Ullernchausseen 70, 0379 Oslo, Norway; 7 Center of Bioinformatics, Department of Informatics, University of Oslo, P.O. Box 1080, Blindern 0316 Oslo, Norway; 8 Department of Pathology, Oslo University Hospital, Rikshospitalet, P.O. box 4950, Nydalen 0424 Oslo, Norway

**Keywords:** Cancer immunotherapy, Targeted therapies, Melanoma

## Abstract

The development of immune checkpoint inhibitors represents a major breakthrough in cancer therapy. Nevertheless, a substantial number of patients fail to respond to checkpoint pathway blockade. Evidence for WNT/β-catenin signaling-mediated immune evasion is found in a subset of cancers including melanoma. Currently, there are no therapeutic strategies available for targeting WNT/β-catenin signaling. Here we show that a specific small-molecule tankyrase inhibitor, G007-LK, decreases WNT/β-catenin and YAP signaling in the syngeneic murine B16-F10 and Clone M-3 melanoma models and sensitizes the tumors to anti-PD-1 immune checkpoint therapy. Mechanistically, we demonstrate that the synergistic effect of tankyrase and checkpoint inhibitor treatment is dependent on loss of β-catenin in the tumor cells, anti-PD-1-stimulated infiltration of T cells into the tumor and induction of an IFNγ- and CD8^+^ T cell-mediated anti-tumor immune response. Our study uncovers a combinatorial therapeutical strategy using tankyrase inhibition to overcome β-catenin-mediated resistance to immune checkpoint blockade in melanoma.

## Abstract (web summary)

Waaler et al. show that a tankyrase inhibitor, G007-LK, decreases WNT/β-catenin and YAP signaling, sensitizing tumors to anti-PD-1 immune checkpoint therapy in mice. This study suggests that a combinatorial therapy using tankyrase inhibition can be used to overcome β-catenin-mediated resistance to immune checkpoint blockade in melanoma.

## Introduction

Cancer immunotherapy is undergoing rapid advances. Treatment of patients using immune checkpoint inhibitors, such as antibodies against cytotoxic T-lymphocyte-associated protein 4 (CTLA-4), programmed cell death 1 (PD-1) and programmed death-ligand 1 (PD-L1) that enhance T cell-mediated immune responses against cancer, is considered a major breakthrough^1,2^. However, many cancer patients, including 40–65% of melanoma patients, do not respond to checkpoint inhibitor treatment and the underlying mechanisms are not well understood^1–4^. Resistance mechanisms are currently being mapped, and cellular signaling pathways and components, such as epidermal growth factor receptor (EGFR), phosphoinositide-3-kinase (PI3K)/AKT serine/threonine kinase (AKT), vascular endothelial growth factor (VEGF) and B-Raf proto-oncogene, serine/threonine kinase (BRAF)V600E, as well as Wingless-type mammary tumor virus integration site (WNT)/β-catenin signaling, emerge as promising targets for therapeutical intervention^1,5,6^. WNT/β-catenin signaling can play a central regulatory role in immune cell homeostasis, development and function as well as in peripheral T cell activation, differentiation and tumor-immune cell interplay^7^. β-catenin is the key transcriptional regulator of WNT/β-catenin signaling^8^. β-catenin-induced immune evasion is found in 13% of all tumors^9^ and 42% of cutaneous melanoma^10^.

A recent study, using genetically engineered murine melanoma models, revealed that tumors expressing a dominant stable form of β-catenin showed negligible T-cell infiltration and were resistant to checkpoint blockade therapy^11^. In these β-catenin-positive tumors, production of C–C motif chemokine ligand 4 (CCL4) and additional chemokines was reduced. This, at least partly^12^, resulted in reduced recruitment of cluster of differentiation (CD)103^+^/ basic leucine zipper transcriptional factor ATF-like 3 (BATF3)-lineage dendritic cells (DC) to the tumor microenvironment and finally defective host priming of antigen-specific T cells^11,13^. In contrast, in melanoma tumors with low levels of β-catenin, DCs and CD8^+^ T cells migrated into the tumor and the tumor-killing activities of CD8^+^ T cells could be unleashed or enhanced by the use of checkpoint inhibitors^11^. Hence, interventions that reduce WNT/β-catenin signaling may have the potential to broaden the anti-tumor spectrum of checkpoint inhibitors.

Although dysregulation of WNT signaling is a hallmark characteristic in a major fraction of cancers, anti-cancer therapy that targets this pathway is currently not available in clinical practice^8,14–16^. Target identification and characterization of the small-molecular WNT/β-catenin signaling inhibitor XAV939 revealed that telomeric repeat factor (TRF1)-interacting ankyrin-related ADP-ribose polymerases 1 and 2 (tankyrase 1 and 2, TNKS1/2) are key and druggable regulatory enzymes in the signaling pathway^17^. TNKS1/2 catalyze the post-translational modification poly(ADP-ribosyl)ation. AXIN1 and AXIN2 proteins are the main rate-limiting structural proteins that together with adenomatous polyposis coli (APC) control the formation of the β-catenin degradosome, which also contains the β-catenin-targeting kinase glycogen synthase kinase 3 beta (GSK3β)^18^. TNKS1/2 poly(ADP-ribosyl)ate AXIN proteins to earmark them for degradation by the ubiquitin-proteasomal system^19^. Inhibition of TNKS1/2 can therefore lead to stabilization of AXIN proteins and hence the degradosomes. This, in turn, enhances the phosphorylation and degradation of the central transcriptional regulator β-catenin and inhibits WNT/β-catenin signaling^17,20–23^.

TNKS1/2 catalytic activity does not only regulate the stability of AXIN proteins, but also interferes with additional biological mechanisms and cell signaling pathways including telomere homeostasis, mitotic spindle formation, vesicle transport, and energy metabolism, as well as AKT/PI3K, AMPK and Hippo signaling^19,24–26^. In Hippo signaling, tankyrase inhibitor-mediated stabilization of angiomotin (AMOT) proteins shifts the subcellular location of the transcription cofactors yes associated protein 1 (YAP) and tafazzin (TAZ), leading to a reduction of oncogenic YAP signaling^27,28^. Recent reports show that YAP signaling may support immune evasion in cancer and melanoma by inducing PD-L1 expression^29,30^, whereas others show that enhanced YAP signaling, due to loss of the regulating kinases large tumor suppressor kinase 1 and 2 (LATS1/2), may promote an anti-cancer immune response^31^.

Checkpoint inhibitor treatment, including blockade of the PD-1 receptor, has shown limited efficacy in the murine B16-F10 melanoma model, despite strong expression of the ligand PD-L1 on the tumor cells^32^; a feature attributed to low tumor infiltration by effector CD8^+^ T cells^33,34^. G007-LK is a potent preclinical stage tankyrase inhibitor with a high selectivity towards tankyrase 1 and 2 and a favorable pharmacokinetic profile in mice with an oral bioavailability of 76% and a t_1/2_ of 2.6 hours in female mice^23,35,36^.

Here, we describe G007-LK-mediated blockade of both WNT/β-catenin and YAP signaling in B16-F10 cells in vitro and in vivo. We show that two murine melanoma models display resistance to monotherapy with either anti-PD-1 or G007-LK. A synergistic anti-tumor effect was observed upon combined anti-PD-1/G007-LK treatment. We show that the mechanistic basis for the synergy was not G007-LK-mediated enhanced release of the BATF3-lineage DC-attracting chemokine CCL4 or increased tumor infiltration by CD8^+^ T cells. Instead, we find that alterations in T cell infiltration are mainly orchestrated by anti-PD-1 treatment alone. Next, we provide evidence that combined anti-PD-1/G007-LK treatment of B16-F10 tumors is effectuated by G007-LK-induced loss of β-catenin in the tumor cells and induction of an interferon-γ (IFNγ)- and CD8^+^ T cell-dependent anti-tumor-immune response. Finally, upon RNA sequencing of G007-LK-treated human melanoma cell lines and B16-F10 cells, we reveal a transcriptional response profile for a cell line subpopulation displaying high relative baseline YAP signaling activity and predisposition for reduced *MITF* expression upon tankyrase inhibition.

## Results

### G007-LK inhibits WNT/β-catenin and YAP signaling

**Fig. 1:**
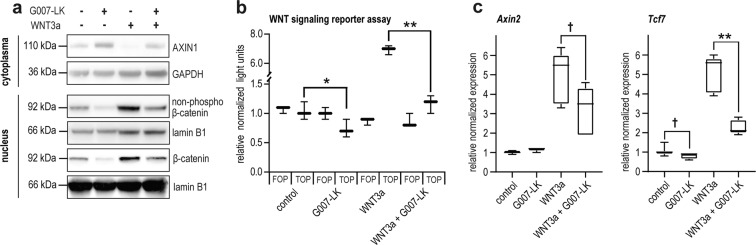
G007-LK can reduce WNT/β-catenin signaling in B16-F10 cells in vitro. **a** Representative immunoblots of cytoplasmic AXIN1 (upper) and nuclear active form of β-catenin (non-phospho, serine [Ser] 33/37/threonine [Thr] 41) and total β-catenin (lower). GAPDH or lamin B1 document equal protein loading. Treatments used for cultured B16-F10 cells in **a**–**c**: Vehicle (DMSO, 0.01%), G007-LK (1 µM), recombinant WNT3a (activator of WNT/β-catenin signaling) or WNT3a + G007-LK for 24 h. **b** Luciferase-based reporter assay for measuring WNT/β-catenin signaling activity. B16-F10 cells transiently transfected with superTOPflash (vector with TCF promoter binding sites) or FOPflash (control vector with mutated TCF binding sites) along with *Renilla* luciferase (for normalization). All samples normalized to superTOPflash signal for wild-type control. For **b**, **c** Boxplots show median, first and third quartiles and maximum and minimum whiskers. One-tailed *t*-tests are indicated by *(*P* < 0.05) or **(*P* < 0.01). Background SuperTOPflash versus FOPflash activities were not significantly different, indicating low basal WNT/β-catenin signaling activation. One representative experiment of two repeated assays with three replicates is shown. **c** Real-time RT-qPCR analyses of WNT/β-catenin signaling target genes (*Axin2* and *Tcf7*). One-tailed *t*-test is indicated by **(*P* < 0.01) and one-tailed Mann-Whitney rank sum tests are indicated by ^†^(*P* < 0.05). Combined data from minimum three independent experiments with three replicates each are shown.

Tankyrase inhibition can inhibit proliferation and viability in a subset of cancer cell lines in vitro^8,25^. When the anti-proliferative effect of G007-LK on cultured B16-F10 mouse melanoma cell line was monitored, only a limited cell growth reduction was observed (Supplementary Fig. 1a, b). Efficacy of G007-LK treatment on WNT/β-catenin and YAP signaling in B16-F10 cells was then explored in vitro and in vivo. In cell culture, G007-LK-treated B16-F10 cells displayed stabilization of TNKS1/2 and AXIN1 proteins (Fig. 1a, Supplementary Fig. 2a and Supplementary Fig. 27), as well as formation of cytoplasmic TNKS1/2-containing puncta (Supplementary Fig. 3), indicating the formation and accumulation of β-catenin degradosomes^22,23,37^.

Moreover, in WNT3a-induced B16-F10 cells, G007-LK treatment reduced the level of nuclear and cytoplasmic β-catenin protein, transcription of the WNT/β-catenin signaling target genes, such as *Axin2* and transcription factor 7 (*Tcf7*) and also luciferase-based WNT/β-catenin signaling reporter activity (Fig. 1a–c and Supplementary Figs. 2b, c, 4a, c, 5b, 27 and Supplementary Table 1a, b). No distinct effect of G007-LK on WNT/β-catenin signaling was observed in neither B16-F10 cells without WNT3a stimulation nor in β-catenin knockout cells (B16-F10^*Ctnnb1*KO^), indicating low endogenous pathway activity in vitro (Fig. 1a–c, Supplementary Figs. 4a, 5a–e and Supplementary Table 1a, b). Treatment with G007-LK was unable to counteract luciferase-based WNT signaling reporter activity rescued by overexpression of N-terminal mutated β-catenin in B16-F10^*Ctnnb1*KO^ cells (Supplementary Fig. 5f). These results suggest that tankyrase inhibition only induces turn-over of wild-type β-catenin containing intact GSK3β phosphorylation sites.

G007-LK treatment also stabilized angiomotin like 1 (AMOTL1) and angiomotin like 2 (AMOTL2) proteins and decreased transcription of YAP signaling target genes, such as cellular communication network factor 1 (*Ccn1*, previously named *Cyr61*), cellular communication network factor 1 (*Ccn2*, previously named *Ctgf*), *Amotl2* and YAP signaling luciferase reporter activity (Supplementary Figs. 4b, 6a–c, 28 and Supplementary Table 1a,b). The nuclear YAP protein level, instead of being reduced upon tankyrase inhibition as previously reported^27,38^, actually increased in both B16-F10 and HEK293 cells upon G007-LK treatment (Supplementary Fig. 6a, d and 28). Confocal imaging further revealed that G007-LK treatment induced the aggregation of puncta, predominantly in the cytoplasma, with not only colocalized AMOTL1-YAP and AMOTL2-YAP but also AMOTL1-TNKS1/2 and AMOTL2-TNKS1/2 (Supplementary Fig. 7a, b).

**Fig. 2:**
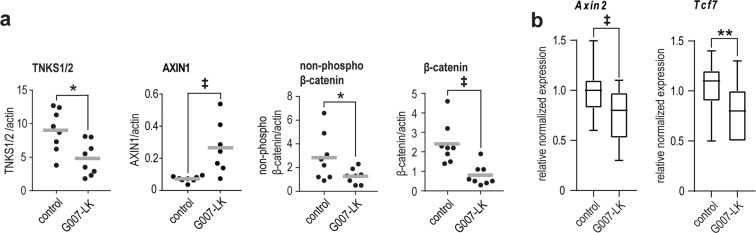
G007-LK can reduce WNT/β-catenin signaling in B16-F10 tumors in C57BL/6 N mice. **a** Representative quantified protein immunoblot ratios (protein vs. loading control) from whole subcutaneous (s.c.) B16-F10 tumors showing altered expression of TNKS1/2, AXIN1, active form of β-catenin (non-phospho, Ser33/37/Thr41) and β-catenin (total). Mean values are indicated by grey lines. For **a** and **b** upon 4 days of treatment with G007-LK diet (*n* = 8, except *n* = 7 for AXIN1) compared to controls (*n* = 8). Two-tailed *t*-tests are indicated by *(*P* < 0.05) and two-tailed Mann-Whitney rank sum tests are indicated by ^‡^(*P* < 0.01). **b** Real-time RT-qPCR analyses of WNT/β-catenin signaling target genes (*Axin2* and *Tcf7*). One-tailed *t*-test is indicated by **(*P* < 0.01) and one-tailed Mann-Whitney rank sum test is indicated by ^‡^(*P* < 0.01). Boxplots show median, first and third quartiles and maximum and minimum whiskers for combined data from two independent measurements with three replicates each are shown.

Next, C57BL/6 N mice with established B16-F10 tumors were treated with G007-LK for four days. This treatment destabilized TNKS1/2 and stabilized AXIN1 protein levels, similar to previous reports^23^, and decreased β-catenin protein levels as well transcription of WNT/β-catenin target genes in the tumors (Fig. 2a, b and Supplementary Figs. 8 and 29). In parallel, AMOTL2 protein was stabilized and transcription of the YAP signaling target genes *Ccn1*, *Ccn2*, and *Amotl2* were reduced in the tumors (Supplementary Figs. 9a–c and 29).

These results show that tankyrase inhibitor treatment using G007-LK can attenuate WNT/β-catenin signaling and YAP signaling target gene expression in B16-F10 cells in vitro and in vivo.

### Synergistic tankyrase and PD-1 inhibition treatment effect

**Fig. 3:**
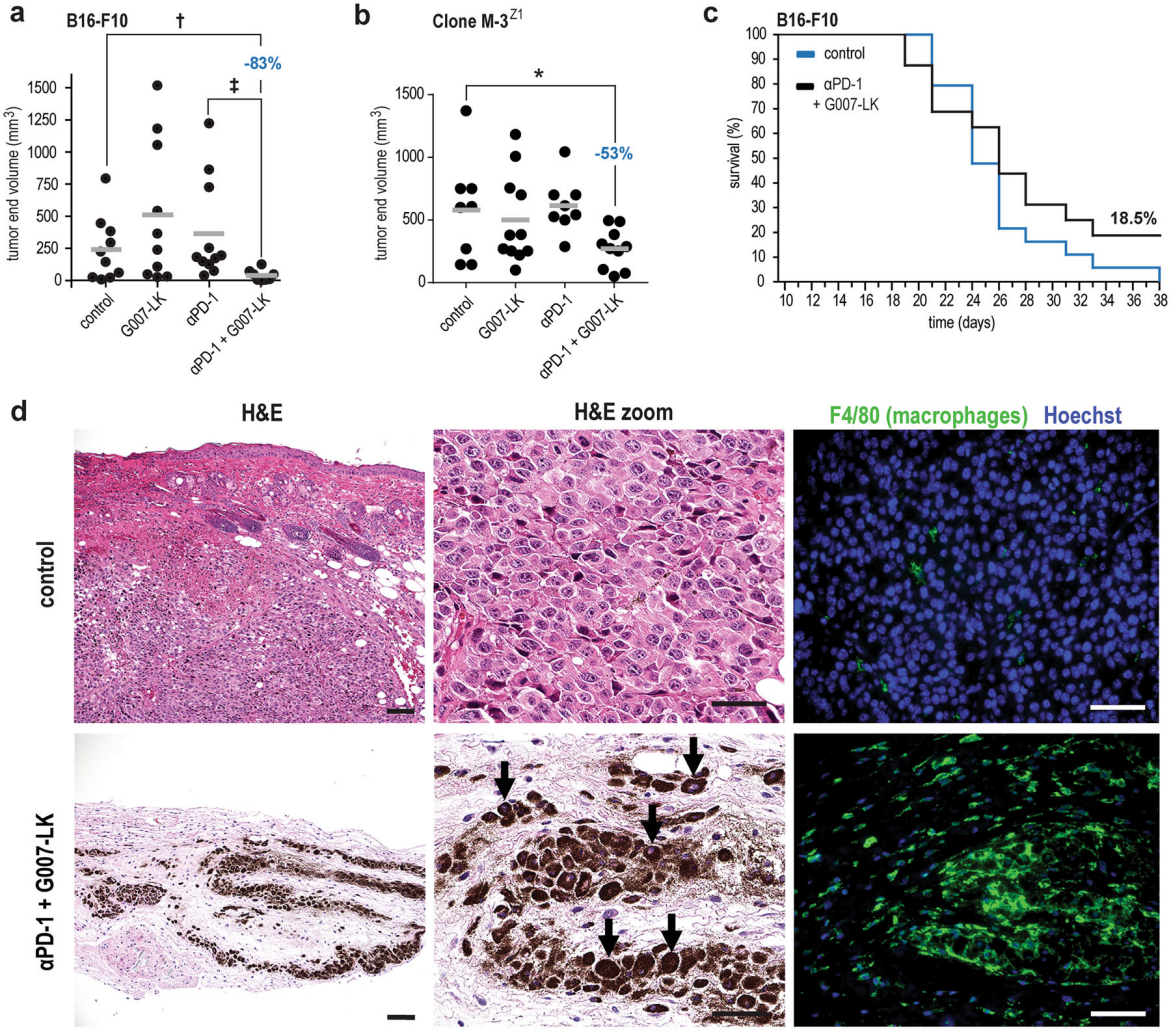
Dual inhibition of tankyrase and PD-1 confers synergistic anti-tumor efficacy in mouse melanoma. **a** B16-F10 tumor (s.c.) end volume upon anti-PD-1/G007-LK treatment (−83% when compared to control) in C57BL/6 N mice treated from day 10 through 21. Control diet (*n* = 10), G007-LK diet (*n* = 10), anti-PD-1 (*n* = 11), and anti-PD-1/G007-LK (*n* = 8). Mann-Whitney rank sum tests are indicated by ^†^(*P* < 0.05) and ^‡^(*P* < 0.01). For **a**, **b** Mean values are indicated by grey lines. Absence of depicted statistical comparisons indicates lack of statistical significance. **b** Clone M-3^Z1^ tumor (s.c.) end volume reduction upon anti-PD-1/G007-LK treatment (−53% when compared to control) in DBA/2 N mice treated from day 8–18. Control diet (*n* = 8), G007-LK diet (*n* = 8), anti-PD-1 (*n* = 11), and anti-PD-1/G007-LK (*n* = 10). Two-tailed *t*-test is indicated by *(*P* < 0.05). **c** Kaplan-Meier plot showing survival of B16-F10-recipient (s.c.) C57BL/6 N mice treated with control diet (*n* = 19, blue) or anti-PD-1/G007-LK (*n* = 16, black) from day 10 to 38. Difference between the two graphs: One-tailed log rank (Mantel-Cox) test, *P* = 0.087, hazard ratio = 1.75, 95% CI: 0.78–3.93. **d** Representative images from H&E (left and middle panels) and F4/80 and Hoechst-stained (in green and blue, respectively, right panels) tumors from control (*n* = 5, upper panels) and tumor implant sites of anti-PD-1/G007-LK-treated surviving mice (*n* = 2, lower panels). Macrophages laden with melanin are highlighted with arrows. Scale bars: H&E = 100 µm (original magnification × 100) and H&E higher magnification and F4/80 Hoechst = 50 µm (original magnification × 400).

To test whether tankyrase inhibition can counteract resistance to immune checkpoint blockade, B16-F10 tumors were established subcutaneously in C57BL/6 N mice. Neither monotherapy with G007-LK, PD-L1 nor PD-1-blocking antibodies reduced tumor size. However, combined anti-PD-1/G007-LK treatment, but not the anti-PD-L1/G007-LK combination, reduced tumor volume and weight (Fig. 3a and Supplementary Fig. 10a–e). In addition, the combined anti-PD-1/G007-LK treatment also reduced WNT/β-catenin signaling and tumor volume of murine Clone M-3^Z1^ melanoma in immunocompetent DBA/2 N mice (Fig. 3b, Supplementary Figs. 11a, c-f and 29). No signs of toxicity, intestinal injury (Supplementary Fig. 10g) or change in body weights were observed in any of the mouse experiments (Supplementary Figs. 10f and 11b). To examine longer-term efficacy of combined anti-PD-1/G007-LK treatment, B16-F10-bearing C57BL/6 N mice were followed until the entire control group reached the endpoint criterion. In the three surviving anti-PD-1/G007-LK-treated mice (18.5%) (Fig. 3c and Supplementary Fig. 12a–d), histopathological evaluation of immunostained tumor sections detected no viable tumor cells. Instead, the tumor implant site was infiltrated by macrophages loaded with melanin, presumably derived from B16-F10 cells (Fig. 3d).

In summary, the tested murine melanoma models are resistant to single-agent anti-PD-1 or G007-LK treatment. In contrast, a synergistic anti-tumor effect and eradication of a subset of the tumors was observed upon combined anti-PD-1/G007-LK treatment.

### Tankyrase inhibition alters intratumoral cytokine composition

**Fig. 4:**
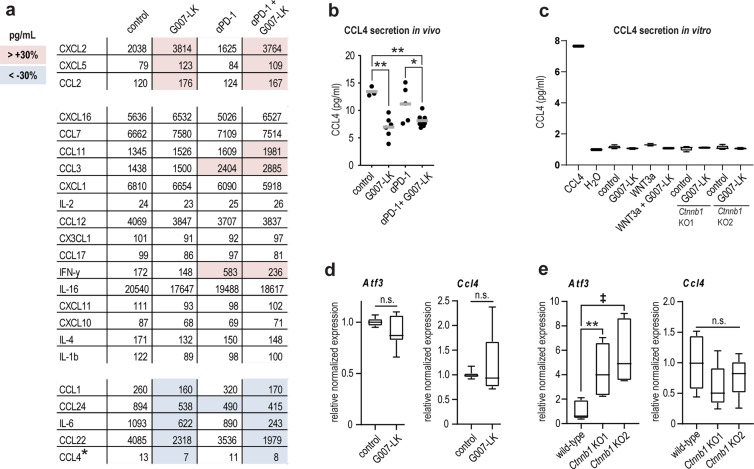
Tankyrase inhibition modulates cytokine release in B16-F10 tumors. **a** Mean values (pg/mL) for enhanced release of chemokines (by >30% over control, highlighted in pink) and downregulated cytokines and chemokines (by >30% below control, highlighted in blue) are shown. For **a**, **b** Conditioned supernatants from matrigel-embedded s.c. B16-F10 tumors screened using multiplex immunoassay upon treatment from day 6 through day 14. Treatments: Control diet (*n* = 3), G007-LK diet (*n* = 6), anti-PD-1 (*n* = 5), and anti-PD-1/G007-LK (*n* = 8). *indicates samples measured using ELISA in **b**. **b** CCL4 ELISA measurements using supernatants from B16-F10 tumors. Two-tailed *t*-tests are indicated by *(*P* < 0.05) and **(*P* < 0.01). Mean values from one representative experiment of three repeated measurements are indicated by grey lines. **c** CCL4 ELISA assay using supernatants from in vitro cultured (72 h) B16-F10 wild-type cells treated with vehicle control (DMSO, 0.01%), G007-LK (1 µM), WNT3a, WNT3a + G007-LK as well as B16-F10^*Ctnnb1*KO^ cells (KO1 and 2) treated with G007-LK (1 µM). Positive control, recombinant CCL4 protein. Negative control: H_2_O. For **c**–**e** Boxplots show median, first and third quartiles and maximum and minimum whiskers. One representative experiment of two repeated assays with three replicates is shown. **d** Real-time RT-qPCR analyses of *Atf3* and *Ccl4* from B16-F10 cell culture treated (24 h) with vehicle control (DMSO, 0.01%) or G007-LK (1 µM). For **d**, **e** Combined data from minimum three independent experiments with three replicates each are shown. Two-tailed *t*-test is indicated by **(*P* < 0.01), two-tailed Mann-Whitney rank sum test is indicated by ^‡^(*P* < 0.01) and n.s. not significant. **e** Real-time RT-qPCR analyses of *Atf3* and *Ccl4* from cultured B16-F10^*Ctnnb1*KO^ cells compared to wild-type B16-F10 cells.

We next pursued the mechanistic basis for the observed synergy of anti-PD-1 and G007-LK treatment. Previous work in a genetically modified mouse melanoma model have indicated that decreased WNT/β-catenin signaling in the tumor cells promoted adaptive immune responses within the tumor by enhanced secretion of the cytokine CCL4^7,11^. The subsequent chemotaxis of dendritic cells to the tumor site was reported to support infiltration and activation of tumor-reactive CD8^+^ T cells^11^. To identify alterations in cytokine secretion upon treatment, conditioned supernatants from matrigel-embedded B16-F10 tumors^39^ were screened using multiplex immunoassays while cell cultures were analyzed using ELISA assays. Few alterations were detectable in anti-PD-1-treated tumors, however, G007-LK treatment was associated with increased levels of three cytokines and decreased levels of five cytokines (>30% difference compared to control) (Fig. 4a and Supplementary Fig. 13). Similar changes in cytokine secretion were apparent upon anti-PD-1/G007-LK treatment (Fig. 4a and Supplementary Fig. 13). Notably, G007-LK treatment reduced CCL4 levels in B16-F10 tumors (Fig. 4b) but no reduction was detected in cultured B16-F10 cells (Fig. 4c). *Ccl4* transcript was not inversely correlated to its previously described negative regulator activating transcription factor 3 (*Atf3)*^11^ in either wild-type B16-F10 cells or B16-F10^*Ctnnb1*KO^ cells when compared to wild-type cells (Fig. 4d, e).

In conclusion, the results suggest that G007-LK treatment, but not anti-PD-1 treatment, mainly alters the intratumoral cytokine composition. The herein observed anti-PD-1/G007-LK-induced anti-tumor effect cannot be attributed to enhanced CCL4 secretion.

### The treatment effect depends on β-catenin in tumor, IFNγ, and CD8^+^ T cells

**Fig. 5:**
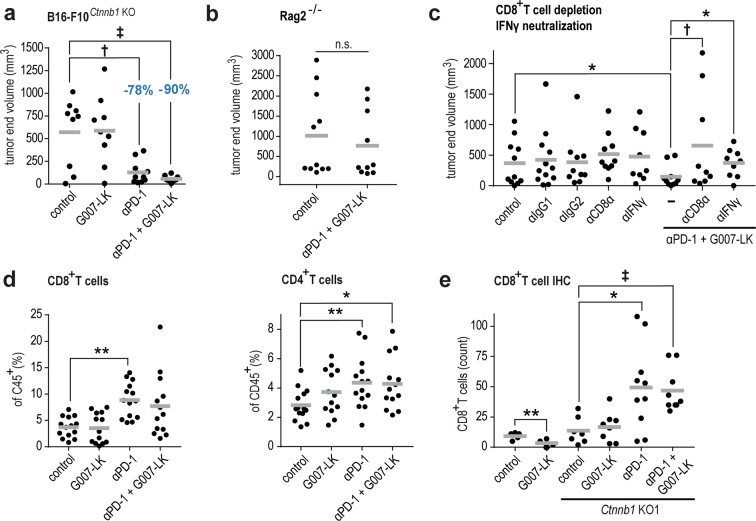
The synergistic anti-tumor effect of tankyrase and PD-1 inhibition is recapitulated by β-catenin depletion in tumor cells and is dependent on IFNγ and CD8^+^ T cells. **a**, B16-F10^*Ctnnb1*KO^ tumor (s.c.) end volume reduction upon anti-PD-1 (−78%) and combined anti-PD-1/G007-LK treatment (−90%) in C57BL/6 N mice treated from day 11 through day 25. Control diet (*n* = 9), G007-LK diet (*n* = 9), anti-PD-1 (*n* = 10), and anti-PD-1/G007-LK (*n* = 10). Mann-Whitney rank sum tests are indicated by ^†^(*P* < 0.05) and ^‡^(*P* < 0.01). For **a**–**e** Mean values are indicated by grey lines. **b** Tumor end volumes of B16-F10-challenged (s.c.) C57BL/6 Rag2^−/−^ mice treated for 12 days with control diet (*n* = 11) compared to combined anti-PD-1/G007-LK treatment (*n* = 10). n.s. not sig*n*ificant. **c** B16-F10 tumor (s.c.) end volume reduction upon anti-PD-1/G007-LK treatment in C57BL/6 N mice from day 10 through day 21. Control diet (*n* = 11), anti-IgG1 isotype control (*n* = 12), anti-IgG2 isotype control (*n* = 10), anti-CD8α (*n* = 10), anti-IFNγ (*n* = 9), anti-PD-1/G007-LK (*n* = 10), anti-PD-1/G007-LK /anti-CD8α (*n* = 9), and anti-PD-1/G007-LK /anti-IFNγ (*n* = 9). One-tailed *t*-tests are indicated by *(*P* ≤ 0.05) while one-tailed Mann-Whitney rank sum test is indicated by ^†^(*P* < 0.05). **d** Flow cytometry analysis of CD8^+^ and CD4^+^T cells (shown as % of CD45^+^ T cells) from single-cell suspensions of comparable-sized tumors collected from B16-F10-challenged (s.c.) C57BL/6 N mice treated for 7–17 days with control diet (*n* = 14), G007-LK diet (*n* = 13), anti-PD-1 (*n* = 14) or anti-PD-1/G007-LK (*n* = 13). Two-tailed *t*-tests are indicated by *(*P* < 0.05) and **(*P* < 0.01). **e** CD8^+^ T cell counts from IHC sections from s.c. wild-type B16-F10 tumors (control diet [*n* = 5], G007-LK diet [*n* = 4]), and B16-F10^*Ctnnb1*KO^ tumors (control diet [*n* = 7], G007-LK diet [*n* = 8]) anti-PD-1 [*n* = 10] and a*n*ti-PD-1/G007-LK [*n* = 9]). Two-tailed *t*-tests are indicated by *(*P* < 0.05) and one-tailed Mann-Whitney rank sum test is indicated by ^‡^(*P* < 0.01).

A knockout of β-catenin in B16-F10 cells can serve as a model to recapitulate G007-LK-mediated blockade of WNT signaling. To evaluate β-catenin-mediated immune evasion in the B16-F10 syngeneic mouse melanoma model, β-catenin was knocked out in B16-F10 cells (B16-F10^*Ctnnb1*KO^, Supplementary Fig. 5a, b) and one of the cell lines, B16-F10^*Ctnnb1*KO1^, was used to establish subcutaneous tumors in C57BL/6 N mice. Compared to vehicle control mice, anti-PD-1-treated mice displayed reduction in tumor size, indicating loss of anti-PD-1 resistance in β-catenin-deficient tumors (Fig. 5a and Supplementary Fig. 14a–d). The result suggests that the synergistic anti-PD-1/G007-LK treatment effect seen in wild-type B16-F10 tumors (Fig. 3a) is, to a considerable part, attributed to G007-LK-induced reduction of β-catenin levels in the melanoma cells of the tumors.

To evaluate if the observed effects of anti-PD-1/G007-LK treatment are mediated by an adaptive immune response, B16-F10 challenge experiments were repeated in recombinase-deficient (Rag2^−/−^) mice, which lack functional T and B cells, but possess functionally intact natural killer (NK) cells^40^. No significant effect of anti-PD-1/G007-LK treatment was observed in such mice (Fig. 5b and Supplementary Fig. 15a, b). Although, a contributory role of NK cells and the innate immune system cannot be entirely excluded, the result indicates that the anti-PD-1/G007-LK treatment effect is orchestrated by an adaptive immune response.

Next, we further evaluated the adaptive immune response by performing selective elimination of either CD8^+^ T cells or IFNγ. Antibody-mediated CD8^+^ T cell depletion abrogated the anti-tumor effect of anti-PD-1/G007-LK treatment (Fig. 5c and Supplementary Fig. 16a, b). Similarly, neutralization of IFNγ resulted in increased tumor growth comparable to anti-PD-1/G007-LK -treated mice (Fig. 5c and Supplementary Fig. 16a, b). In summary, these results confirm a role of CD8^+^ T cells and IFNγ as mediators of the synergistic effect of anti-PD-1/G007-LK treatment.

To assess immune cell infiltration upon treatment, we next performed flow cytometry analysis using tumors of comparable size collected on day 7–17 (Supplementary Figs. 17a and 18a, b). No increase in tumor leukocyte abundance was observed in any of the treatment groups when compared to the control group (Supplementary Fig. 17b). An increase in total T cell and CD8^+^ T cell infiltration was seen in both the anti-PD-1 and anti-PD-1/G007-LK groups, whereas CD4^+^ T cells were similarly increased across all treatment groups (Fig. 5d and Supplementary Fig. 17c). No differences in the abundance of CD4^+^ or CD8^+^ T cells expressing the memory marker CD44 were detected (Supplementary Fig. 17d). T_regs_ constituted approximately 1% of infiltrating CD45^+^ cells in all groups (Supplementary Fig. 17e). Myeloid DCs were decreased in the anti-PD-1 and anti-PD-1/G007-LK-treated groups while CD103^+^ DCs were equally present in all treatment groups (Supplementary Fig. 17f). Lymphoid DCs, myeloid-derived suppressor cells (M-MDSC) and neutrophils were present at comparable levels across all treatment groups (Supplementary Fig. 17g).

Subsequently, infiltration of CD8^+^ T cells in tumors sections was scored using treated wild-type and β-catenin knock-out B16-F10 tumors. Only anti-PD-1 and anti-PD-1/G007-LK-treated B16-F10^*Ctnnb1*KO^ tumors showed an increase in CD8^+^ T cell infiltration when compared with controls (Fig. 5e and Supplementary Fig. 19). The result suggests that only PD-1 inhibition, and not loss of β-catenin in the tumor cells, contributes to increased chemotaxis of CD8^+^ T cells.

To evaluate direct effects of G007-LK on T cell effector functions, we assayed the in vitro activation of MHC class I (H2-K^b^)-restricted and ovalbumin-specific CD8^+^ T cells. The presence of G007-LK moderately enhanced T cell proliferation following cognate interaction with antigen-presenting cells, but did not affect proliferation following polyclonal activation by immobilized anti-CD3/anti-CD28 antibodies (Supplementary Fig. 20a). The presence of G007-LK did not affect the expression of T cell activation markers (CD26L, CD44, CD25, and CD69) or secretion of interleukin 2 (IL2) or IFNγ (Supplementary Fig. 20b–d). The addition of G007-LK induced a slight increase in intracellular granzyme B expression in CD8^+^ T cells following cognate or polyclonal activation, possibly indicating increased effector function (Supplementary Fig. 20e).

In summary, combined anti-PD-1/G007-LK treatment of B16-F10 tumors is dependent on tankyrase inhibitor-mediated loss of β-catenin in the tumor and induces an IFNγ and CD8^+^ T cell-dependent growth-inhibitory effect. Changes in myeloid DC and T cell infiltration are likely attributable to anti-PD-1 treatment, but do not alone seem to cause anti-tumor activity.

### RNA sequencing reveals a subpopulation transcriptional response profile

The efficiency of tankyrase inhibitor-mediated inhibition of WNT/β-catenin and YAP signaling is known to be cell type and context-dependent. The beneficial synergistic anti-PD-1/G007-LK treatment effect seen on immunosurveillance in B16-F10 tumors may therefore vary between melanomas based on differences in cell signaling pathway activities and genetic background. In addition, the here utilized B16-F10 murine melanoma model, lacking the BRAF^V600E^ mutation^41,42^, only partially recapitulates the genetic features of human melanoma. Thus, murine B16-F10 cells and a panel of 18 human melanoma cell lines, were exposed to G007-LK treatment followed by RNA sequencing and bioinformatic analyses.

**Fig. 6:**
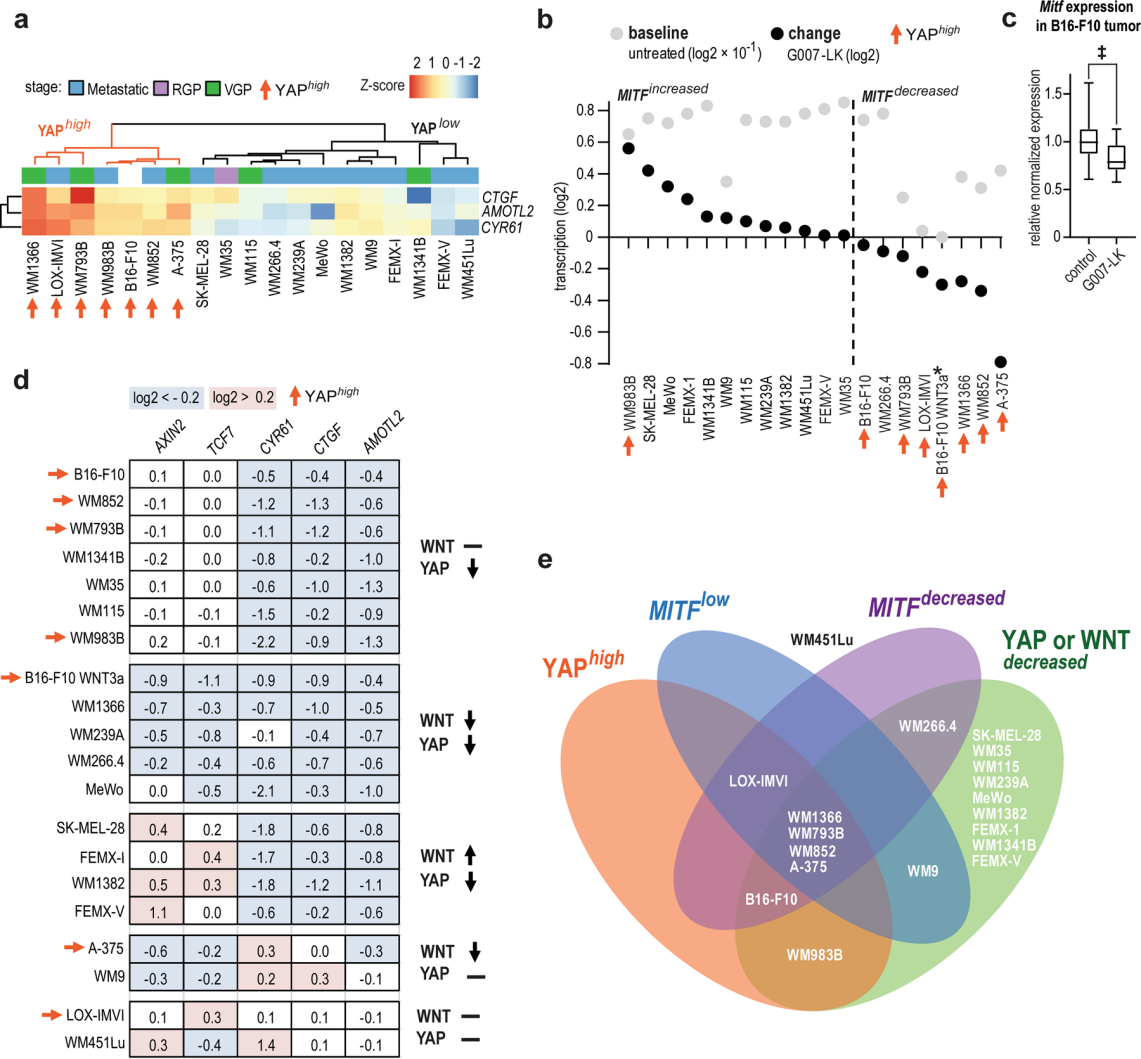
High activity of YAP signaling correlates with low baseline *MITF* expression and potential for decreased *MITF* transcription upon tankyrase inhibition. **a** Expression of YAP signaling target transcripts (*Ccn1*, *Ccn2*, and *Amotl2*) for untreated human and murine B16-F10 melanoma cell lines. Seven of 19 samples displayed high relative transcription of YAP signaling target genes (YAP^high^) when compared to samples with less transcription (YAP^low^). YAP^high^ is highlighted by orange branches in the dendrogram and arrows. Scale bar indicates differences in Z-score (standard deviations < or > mean) values for log2 transcripts per millions (TPMs) within each row. Cancer stages: Metastatic in blue, radial growth phase (RGP) in pink and vertical growth phase (VGP) in green. **b** Baseline *MITF* expression in untreated samples (grey bars, log2-transformed TPMs × 10^−1^) and change upon treatment with G007-LK (1 µM) for 24 h (black dots sorted descending from left to right, log2 values from treated versus untreated TPMs). Samples with increased (*MITF* ^increased^, left) or decreased (*MITF* ^decreased^, right) expression of *MITF* upon tankyrase inhibitor treatment are separated by scattered vertical line. *Indicates that no value for untreated sample is inserted. For **b**, **d** B16-F10 WNT3a = WNT3a + G007-LK relative to WNT3a-stimulated control. YAP^high^ cell lines are highlighted by orange arrows. **c** Real-time RT-qPCR analysis of *Mitf* expression in G007-LK-treated B16-F10 s.c. tumors versus control tumors (*n* = 8). Two-tailed Mann-Whitney rank sum test is indicated by ^‡^(*P* < 0.01). Boxplot shows median, first and third quartiles and maximum and minimum whiskers for data from two repeated measurements. **d** Changes in gene expression for WNT/β-catenin (*Axin2* and *Tcf7*) and YAP (*Ccn1*, *Ccn2*, and *Amotl2*) signaling target genes for 18 G007-LK-treated (1 µM) human and murine B16-F10 melanoma cell lines. Downregulated signaling activity is indicated by ↓ (log2 < −0.2, highlighted in blue), upregulated activity is indicated by ↑ (log2 > 0.2, highlighted in pink), and inconclusive or lack of regulation is indicated by − . **e** Venn diagram depicting the cell lines intersecting profiles for pre-treatment (YAP^high^ and *MITF* ^low^) and response to G007-LK treatment (*MITF* ^decreased^ and YAP or WNT^decreased^).

In the untreated group, no clear correlation was found between baseline transcription of WNT/β-catenin signaling target genes (Supplementary Fig. 21a) or the mutation load (Supplementary Fig. 22) against overall gene expression (Supplementary Fig. 21b, c). However, when the cell lines were classified into two groups based on baseline YAP signaling target gene transcription, YAP^high^ and YAP^low^ (Fig. 6a), the YAP^high^ group coincided with clustering of overall gene expression and against a panel of markers for β-catenin-controlled melanoma cell fate and proliferation^43^ (Supplementary Fig. 21d–f). One of these markers in the YAP^high^ group, melanocyte inducing transcription factor (*MITF*), had an overall low relative transcription profile (*MITF*^low^) (Fig. 6b). MITF is a lineage-restricted regulator in melanocytes that is positively associated with melanoma proliferation, and also suppressed invasion and metastasis^44–46^. Indeed, when using untreated samples, ingenuity pathway analysis (IPA) core analysis of the YAP^high^ versus the YAP^low^ group identified *MITF* as the most statistically significant key upstream transcriptional regulator separating the two groups (Supplementary Fig. 23a, b and Supplementary Table 2).

Upon treatment with G007-LK, samples in the *MITF*^low^ group were predisposed for decreased *MITF* expression (*MITF*^decreased^), while the *MITF*^high^ group was oppositely predisposed for increased *MITF* expression (*MITF*^increased^)(Fig. 6b and Supplementary Fig. 24a). Notably, in B16-F10 tumors, transcription of *Mitf* was moderately reduced upon G007-LK-treatment (Fig. 6c). Attenuated WNT/β-catenin and/or YAP signaling activity was observed in nearly all samples upon G007-LK treatment (Fig. 6d). These changes in signaling pathway activities did not correlate with changes in *MITF* expression, nor act as a predictive marker for *MITF* regulation (Fig. 6e and Supplementary Figs. 24b–e and 25a, b). In conclusion, YAP^high^ is a marker for a melanoma subgroup that includes B16-F10 (Fig. 6e) and tracks with tankyrase inhibitor-induced reduction in *MITF* expression (Supplementary Fig. 26 and Supplementary Table 3).

## Discussion

Here we show, for the first time to our knowledge, proof-of-concept results for a previously unreported therapeutic strategy using tankyrase inhibitor-mediated blockade of WNT/β-catenin signaling to counteract β-catenin-supported immune evasion and resistance to checkpoint inhibition in syngeneic murine melanoma models.

The G007-LK treatment effect could be recapitulated in vivo by knockout of β-catenin in B16-F10 tumor cells. Although not further supported by a rescue experiment re-introducing N-terminal mutant β-catenin, the result suggests that loss of WNT/β-catenin signaling in tumor cells is sufficient to trigger a gain of susceptibility and a synergetic relationship to checkpoint inhibition. Nevertheless, we cannot exclude the possibilities that tankyrase inhibition impacts additional biological mechanisms in the tumor cells, in the tumor microenvironment, systemically or on immune cell subpopulations that can contribute to the treatment effect^7,19,24,25^. In addition to its effect on WNT/β-catenin signaling, G007-LK treatment stabilized AMOT proteins and reduced YAP signaling-mediated gene expression in B16-F10 cell culture and tumors. An absence of reduced nuclear YAP and TAZ protein levels, as also seen in HEK293 cells, along with the here observed aggregation of colocalized YAP, AMOT proteins and TNKS1/2-containing puncta, is inconsistent with previous reports indicating that further evaluation of tankyrase inhibitor-induced interference with YAP signaling is necessary^27,37,38^.

Our results provide evidence that the anti-tumor effect of combined tankyrase and checkpoint inhibitor treatment against B16-F10 tumors is dependent on both IFNγ and CD8^+^ T cells and occurs, at least in part, through direct suppression of WNT/β-catenin signaling within the tumor cells. The precise mechanistic basis of this synergistic effect remains to be investigated. Previous work performed in genetically modified models indicate that lowered WNT/β-catenin signaling^11^ resulted in increased intratumoral CCL4 cytokine release, which lead to enhanced influx of CD103^+^/BATF3^+^ pDCs and subsequent activation of CD8^+^ T cell anti-tumor activity. Our results indicate that G007-LK treatment indeed could change the intratumoral cytokine composition; however, we found no evidence for increased CCL4 cytokine levels. In addition, assessment of the intratumoral immune response following G007-LK monotherapy did not yield significant quantitative alterations in T cell or antigen-presenting cell (APC) subsets in treated mice. While it cannot be excluded that qualitative or more subtle quantitative changes in particular tumor-specific CD8^+^ T cell subsets might be responsible for the observed therapeutic effects, an increase in overall CD8^+^ T cell infiltration was not observed. Only a moderate enhancement of proliferation and granzyme B expression was observed in G007-LK-treated MHC class I (H2-K^b^)-restricted and ovalbumin-specific CD8^+^ T cells, which could indicate enhanced effector function. In depth follow-up studies in the framework of tankyrase inhibition are required, including assessment of the effects of alterations in individual cytokine and chemokine levels on the T cell response^12^. The use of model systems with defined and traceable anti-tumor CD8^+^ T cell specificities would also be of value in determining the effects of tankyrase inhibition on the kinetics and qualitative characteristics of the ensuing immune response.

The RNA sequencing of 18 tankyrase inhibitor-treated human melanoma cell lines and B16-F10 cells shows that tankyrase inhibition context-dependently can influence WNT/β-catenin and YAP signaling, not only in murine B16-F10 cells, but importantly also in a subset of 18 human melanoma cell lines. The results indicate that tankyrase inhibition may have a potential for treatment of a subset of patients with the human disease in combination with checkpoint inhibition. Analysis of the RNA sequencing data revealed a subgroup-specific transcriptional response profile. Upon tankyrase inhibition, the subgroup displaying elevated baseline YAP signaling activity was susceptible to reduced *MITF* expression. Presently, neither the meeting points between MITF, its regulation by YAP/ TEA domain transcription factor (TEAD), activator protein 1 (AP-1) and tankyrase, nor the function in immune regulation and control of susceptibility to checkpoint‐inhibitor therapy are well characterized^46–51^. Further molecular and functional evaluations are required for establishing a precise set of rules for context-dependent treatment efficiency using combined tankyrase and checkpoint inhibitor treatment in human melanoma. Translation of the combinatorial therapeutical strategy, using tankyrase inhibition to counteract β-catenin-induced resistance to immune checkpoint blockade, should also be evaluated for treatment efficacy against other cancers^6,7,9^.

Tankyrase inhibitors have been suspected to cause intestinal toxicity^23^ and bone loss^52^ in certain mouse models. In this study, and similar to previous reports^22,36,53^, we observed no signs of toxicity, intestinal injury or body weight changes in G007-LK-treated mice. G007-LK is a preclinical stage tankyrase inhibitor without the properties needed to reach approval for clinical testing^35^. Currently, extensive research to identify and further develop safe drugs directed towards multiple biotargets in the WNT signaling pathway, including tankyrase, are ongoing^8,14–16^.

Collectively, the results presented here suggest that G007-LK-induced blockade of WNT/β-catenin signaling leads to improved efficacy of PD-1 immune checkpoint blockade, and in addition induction of an IFNγ and CD8^+^ T cell-dependent anti-tumor-immune response against B16-F10 tumors. The findings warrant a further in-depth preclinical and clinical evaluation of combining checkpoint inhibitors with tankyrase inhibition for the treatment of melanoma.

## Methods

### Cell culture

The mouse melanoma cell line B16-F10 (ATCC^®^ CRL-6475™) and human HEK293 cells (ATCC^®^ CRL-1573™) were obtained from the American Type Culture Collection. Clone M-3^Z1^ (ProQinase) cells were derived from Clone M-3 cells (ATCC^®^ CCL-53.1^™^) after implantation in DBA/2 NCrl mice (DBA/2N, Charles River). The cell cultures were generally kept below 20 passages (~10 weeks) and the melanoma cells were grown in RPMI-1640 medium (R8758, Sigma Aldrich) while HEK293 cells were grown in DMEM medium (D6429, both Sigma Aldrich). The cell culture medium was supplemented with 1% penicillin-streptomycin (P4333, Sigma Aldrich) and 5% fetal bovine serum (FBS, 10270-106, Gibco) and grown at 37 °C in humidified incubators with 5% CO_2_. The cells were routinely monitored (upon thawing and monthly) for mycoplasma using MycoAlert mycoplasma detection kit (Lonza). B16-F10 cells were authenticated by short tandem repeat profiling and subsequent analysis confirming murine C57BL/6 origin (Leibniz-Institute DSMZ). General protocol for treatment of cultured B16-F10 cells: Cells were seeded one day before treatment to reach ~20 or 80% confluence for 72 or 24 h treatments, respectively. The cell culture medium was changed for medium containing 0.01% DMSO (D8418, Sigma Aldrich), 1 µM or various doses of G007-LK (Mercachem or ChemRoyal), recombinant mouse WNT3a (0.5 µg/mL, 1324-WN-010, R&D Systems), or a combination of G007-LK (1 µM) and WNT3a.

### Proliferation assays

1000 cells/well were seeded in 96-well plates in at least 6 replicates for each treatment tested. The day after, cell culture medium was changed to contain various doses of G007-LK or vehicle (DMSO, Sigma Aldrich) and the plates were incubated in an IncuCyte (FLR30140, Essen BioScience) at 37 °C for real-time monitoring of cell confluency. At experiment endpoint (80–100% confluency after 5–7 days of cell growth), the cells were incubated for 1 h at 37 °C with CellTiter 96^®^ AQueous Non-Radioactive Cell Proliferation Assay (MTS, Promega) according to the supplier’s recommendations. Abs_490_ was measured spectrophotometrically (Wallac 1420 Victor2 Microplate Reader, Perkin Elmer) and compared to Abs_490_ (t0) using the following formula to determine single well values relative to the DMSO vehicle control: (sample A_490_ − average A_490 t0_)×100/ (average A_490_ [for 0.01% DMSO controls] − average A_490 t0_).

### Western blot analysis

Treated cells were washed in PBS and lysed in NP40 Cell lysis buffer (Invitrogen) containing protease inhibitors. The nuclei were pelleted and separated from the cytoplasmic/cell membrane supernatant fractions. RIPA lysis buffer (89901, Thermo Fisher Scientific) containing phosphatase (4906845001) and protease inhibitors (4693116001, both Sigma Aldrich) was added to the nuclei followed by sonication (Bioruptor®Plus, Diagenode). Tumor samples were prepared using TissueRuptor II (9002755, Qiagen), RIPA buffer and sonication. Protein concentrations were measured using Pierce™ BCA Protein Assay Kit (Pierce Biotechnology). The protein samples were separated by SDS PAGE (Invitrogen) and immunoblotted (Immobilon-PSQ PVDF Membrane, Millipore) using the following primary antibodies: Tankyrase-1/2 (TNKS1/2, H-350, sc-8337, Santa Cruz Biotechnology), AXIN1 (C7B12, 3323, Cell Signaling Technology), non-phospho (active) β-catenin (D13A1, 8814, Cell Signaling Technology), β-catenin (610153,1:500, BD Biosciences), YAP (sc-101199, Santa Cruz Biotechnology), TAZ (HPA007415, Sigma Aldrich), AMOT (sc-166924, Santa Cruz Biotechnology), AMOTL1 (PA5-42267, Thermo Fisher Scientific), AMOTL2 (PA5-78770, Thermo Fisher Scientific), GSK3β (12456, Cell Signaling Technology), phospho-GSK3β (Serine [Ser]9) (9323, Cell Signaling Technology). GAPDH (sc-32233, Santa Cruz Biotechnology), β-tubulin III (T2200, Sigma Aldrich), actin (A2066, Sigma Aldrich), and lamin B1 (ab16048, Abcam) were used as loading controls. Primary antibodies were visualized with HRP-conjugated secondary antibodies (mouse anti-rabbit IgG, sc-2357, Santa Cruz Biotechnology or donkey anti-rabbit IgG, 711-035-152, Jackson ImmunoResearch Inc.) and enhanced with chemiluminescent substrate (ECL^™^ Prime Western Blotting Detection Reagent, RPN2236, GE Healthcare) and ChemiDoc™ Touch Imaging System (Bio-Rad). Band quantifications (band of interest versus loading control) was performed using Image Lab Sofware 5.2.1 (Bio-Rad).

### RNA isolation and real-time qRT-PCR

Total RNA was isolated from cell lines and tumor samples using GenEluteTM Mammalian Total RNA Miniprep Kit (Sigma Aldrich). The RNA concentration was measured using Nanodrop 2000c spectrophotometer (Thermo Scientific). cDNA was synthesized from the purified RNA using SuperScript™ VILO cDNA Synthesis Kit (Invitrogen). Real-time qRT-PCR (TaqMan®Gene Expression system, Applied Biosystems) was performed using Viia7 (Applied Biosystems). The following probes were used (all from Applied Biosystems): *Axin2* (Mm00443610_m1), *Tcf7* (Mm00493445_m1), *Atf3* (Mm00476033_m1), *Ccl4* (Mm00443111_m1), *Amotl2* (Mm00502287_m1), *Ctgf* (Mm01192933_g1), *Cyr61* (Mm00487498_m1), *AMOTL2* (Hs01048101_m1), *CTGF* (Hs01026927_g1), *CYR61* (Hs00998500_g1), and *GAPDH* (Hs02758991_g1).

### Luciferase reporter assays

The following plasmids were used: SuperTOP-luciferase (WNT/β-catenin signaling pathway reporter with 7X TCF binding sites: ST-Luc, gift from V. Korinek), SFF-Luc (negative control reporter with mutated TCF binding sites: SuperFOPflash-luciferase, gift from V. Korinek), 8xGTIIC-luciferase (Hippo and YAP signaling pathway reporter: 34615, Addgene, provided by Dr. Stefano Piccolo^54^), β-catenin with deleted (del-β-catenin, gift from R. Kemler) or point mutated (dominant active [DA] β-catenin [S33, 37, 41, 45 A], gift from R. Kemler) N-terminal domain containing GSK3β phosphorylation sites and *Renilla* luciferase (pRL-TK, Promega). On day 1, B16-F10 cells were seeded to reach 50-60% confluency on day 2 for co-transfections (For reporter assays using 10-cm dishes: 16.5 µg luciferase reporter and 3 µg *Renilla* luciferase. For overexpression assays using 6-well plates: 2.2 µg luciferase reporter, 0.1 µg del- β-catenin, DA β-catenin or pCI-neo (empty vector, Promega) and 0.2 µg *Renilla* luciferase for reporter assays) using FuGENE*®* HD (Promega). On day 3, the cells were trypsinized and seeded in 96-well plates and treatment was added on day 4. On day 5, the cells were lysed and the firefly and *Renilla* luciferase activities were measured using Dual-Luciferase Reporter Assay (Promega) protocol and GloMax®-Multi Detection System (Promega). XLfit (Idbs) was used to calculate the half maximal inhibitory concentration (IC_50_) using the Langmuir Binding Isotherm formula: fit = ((A + (B × x)) + (((C-B)×(1-exp ((−1 × D)×x)))/D)), res = (y-fit).

### CRISPR/Cas9-based knockout

RNA sequences targeting exon 4 of mouse catenin (cadherin associated protein), beta 1 (*Ctnnb1*) were designed using the web-based CHOPCHOP platform^55^: *Ctnnb1*_gRNA1: 5′- GATTAACTATCAGGATGACG-3′. The gRNA sequence was inserted into the pSPCas9(BB)-2A-GFP vector (PX458, 48138, Addgene^56^) for transfection into tumor cells using polyethylenimine (408727, Sigma Aldrich). After 24 h, GFP-expressing cells were single-cell sorted (BD FACSAria II, BD Biosciences). Genomic DNA was isolated from individual clones, the relevant gene fragment was amplified by PCR (Forward primer: 5′-GTTCCCTGAGACGCTAGATGAG-3′. Reverse primer: 5′-ACATCACTGCTTACCTGGTCCT-3′.) and screened by Sanger sequencing for non-sense mutations in *Ctnnb1*. Additional verification of gene knockout was performed by immunofluorescence staining (primary antibody β-catenin [610153, 1:500, BD Biosciences] and secondary antibody goat anti-mouse IgG Alexa 488 [A28175, 1:500, Thermo Fisher Scientific]) and western blot analysis (β-catenin, 1:500).

### Immunofluorescence, SIM and confocal microscopy

Cells grown on coverslips pre-coated with poly-L-lysine (sc-286689, Santa Cruz Biotechnology) were fixed in 4% paraformaldehyde (P6148, Sigma Aldrich) for 15 min at room temperature and permeabilized with 0.1% Triton-X100/PBS (T8787, Sigma Aldrich, 15 min at room temperature) followed by 1 hour (at room temperature for SIM images) or 24 h (at 4 °C for confocal images) incubations with primary and secondary (antibodies 1 h at room temperature) diluted in PBS with 4% bovine serum albumin. Nuclear counterstaining was performed with DAPI (D9542, Sigma Aldrich, 1 μg/mL, 5 min at room temperature) and coverslips were mounted in ProLong Diamond Antifade Mountant (Thermo Fisher Scientific). The following primary antibodies were used: β-catenin (610153, 1:500, BD Biosciences), Tankyrase-1/2 (H-350, sc-8337, 1:50, Santa Cruz Biotechnology [for SIM imaging]), Tankyrase-1/2 (E10, sc-365897, 1:50, Santa Cruz Biotechnology [for confocal imaging]), YAP (sc-101199, 1:100, Santa Cruz Biotechnology), AMOTL1 (PA5-42267, 1:50, Thermo Fisher Scientific) and AMOTL2 (PA5-78770, 1:50, Thermo Fisher Scientific). Secondary antibodies used (both from Thermo Fisher Scientific, 1:500): Anti-rabbit IgG Alexa488 (A-21206) and anti-Mouse IgG Alexa594 (A-11005).

SIM images were acquired on a Zeiss Elyra PS1 microscope system using standard filters sets and laser lines with a Plan-APOCHROMAT 63 × 1.4 NA oil objective. SIM imaging was performed using 5 grid rotations with the 0.51 µm grid for 20 Z planes with 0.184 nm spacing between planes. SIM images were reconstructed with the following “Method” parameters in the ZEN black software (MicroImaging, Carl Zeiss): Processing: Manual, Noise Filter: -5, SR Frequency Weighting: 1, Baseline Cut, Sectioning: 100/83/83, Output: SR-SIM, PSF: Theoretical. The SIM images are displayed as maximum intensity projections rendered from all Z planes.

Confocal microscopy was performed on a Zeiss Meta 700 laser scanning confocal microscope using standard filters sets and laser lines with a 63 × oil immersion objective, images were acquired using Zen software (Zeiss). The confocal images were analyzed using Fiji software^57^. Confocal images are displayed as maximum intensity projections rendered from two Z-stacks at the nucleous region with 0.56 µm spacing between stacks.

### Tumor initiation, treatment and analysis of mouse melanoma in vivo

General procedure: Subcutaneous B16-F10 and B16-F10^*Ctnnb1*KO^ tumors (using *Ctnnb1* KO clone 1) were implanted (left flank, 0.2 × 10^6^ tumor cells in 20 µl PBS) in female B6N-Tyr^c-Brd^/BrdCrlCrl albino mice (C57BL/6N, Charles River) or female RAGN12F; B6.129S6-Rag2^tm1Fwa^ N12 mice (Rag2 [C57BL/6 background], Taconic Biosciences). 1.0 × 10^6^ Clone M-3(Z1) cells (ProQinase) in 100 µl were subcutaneously implanted in female DBA/2 NCrl mice (DBA/2N, Charles River). All mice were 3–8 weeks old at experiment startup. Randomization of mice to create treatment groups with comparable tumor sizes was performed by random number generation within individual blocks (MS-Excel 2016). Treatments used: (i) Control (Purina 5001 diet, Research Diets Inc), (ii) G007-LK (250 mg/kg in diet [ab libitum] that delivers 43-53 mg/kg/day, depending on measured food intake [3.2–4.4 g/mouse/day] and body weight), (iii) anti-PD-1 (10 mg/kg, intraperitoneally, i.p. [RMP1-14, BE0146, batch 614616A2, Bio X Cell]), (iv) anti-PD-L1 (10 mg/kg, i.p. [10 F.9G2, BE0101, batch 6154598816S1, Bio X Cell]), (v) combinations of G007-LK and anti-PD-1 or vi) G007-LK and anti-PD-L1. Previous analyses have documented the pharmacokinetics of G007-LK in female mice using oral gavage delivery (t_1/2_ = 2.6 hours and bioavailability = 76%) and in diet containing 250 mg/kg G007-LK (t_1/2_ = 4.2 h)^35,53^. Compound delivery was monitored via food consumption. The chow was weighed thrice weekly to calculate G007-LK delivery per animal and day. Several animals had to be euthanized before experiment endpoint in all animal experiments performed, primarily due to skin ulcerations in the tumor area seen in all treatment groups, and less often due to anemia or excessive tumor size (tumor diameter ≥ 2 cm). The experiment endpoints were defined to a minimum of 8 or 9 surviving animals in any given treatment group.

For experiments using B16-F10 *Ctnnb1* knock-out cells in C57BL/6 N mice: Intraperitoneal injections of anti-PD-1 were administered on day 11, 15, and 18. For experiments using B16-F10 cells in C57BL/6 N mice: Intraperitoneal injections of anti-PD-1 or anti-PD-L1 were administered on day 10, 13, 17 and every 3–4 days from day 21 until the end for the survival analysis. For experiments in Rag2 mice or for Clone M-3(Z1) in DBA/2 N mice: Intraperitoneal injections of anti-PD-1 were administered on day 8, 11, and 15. Primary tumors were measured by a caliper (OMC Fontana) and tumor sizes calculated according to the formula W^2^ × L/2 (L = length and W = width).

Assay for the efficacy of G007-LK-mediated inhibition of tankyrase and WNT/β-catenin and YAP signaling pathways using B16-F10 cells in C57BL/6 N mice: Mice with primary tumor volumes of 20-40 mm^3^ were randomized into two groups (control or G007-LK for four nights, *n* = 8). Whole tumor protein extracts were prepared by sonication in an ultrasound bath (Bioruptor® Plus, Diagenode) in RIPA buffer (Thermo Fisher Scientific) containing phosphatase and protease inhibitors (PhosStop, 4906837001 and cOmplete™ Protease Inhibitor Cocktail, 4693116001, both from Roche). Tumor tissues were homogenized using MagNA Lyser Green Beads (Roche) and total mRNA isolated using GenEluteTM Mammalian Total RNA Miniprep Kit (Sigma Aldrich).

Tumor growth assay using B16-F10 cells in C57BL/6 N mice: On day 10, 72 tumor-bearing animals (primary tumors reaching 20–100 mm^3^) were randomized into six groups (*n* = 12), and treatments (i)–(vi) were administered to the mice. On day 21, the experiment was terminated, and tumors from euthanized mice were dissected, weighed and volumes re-measured with caliper. For 6 euthanized animals from treatment groups (i), (ii), (iii), and (v): small intestinal was dissected, formalin-fixed (10%), paraffin-embedded in a Swiss roll configuration and sections were stained with H&E (115938 and 115935, Merck Millipore). 1, 2, 1, 4, 3, and 2 mice from treatment groups (i), (ii), (iii), (iv), and (v), respectively, were found dead or euthanized for ethical reasons during the experiment due to anemia or skin ulcerations in the tumor area.

Clone M-3(Z1) tumor growth assay: On day 8, 48 tumor-bearing animals (with primary tumors reaching ~26 mm^3^) were randomized into 4 groups (*n* = 12) and treatments (i), (ii), (iii), and (v) were administered to the respective mice. On day 18, the animals were euthanized and the tumors were dissected, weighed and volumes re-measured with caliper. 4, 1, 3, and 1 mice from treatment groups (i), (ii), (iii), and (v), respectively, were euthanized for ethical reasons during the experiment due to skin ulcerations in the tumor area.

Survival assay using B16-F10 cells in C57BL/6 N mice: 60 animals with primary tumors reaching 20–100 mm^3^ were randomized into 2 groups (*n* = 30, control or G007-LK and anti-PD-1). The survival assay endpoint criterion was set to tumor volume >1000 mm^3^. Eleven mice in the control group and 14 mice in the treatment group were euthanized (for ethical reasons) or found dead due to anemia or skin ulcerations in the tumor area before reaching the endpoint criterion. Tumors were collected from surviving and treated animals at endpoint on day 38, and from control animals euthanized on day 26. The tumors were dissected, fixed (10% formalin), embedded in paraffin, sectioned and stained with H&E (115938 and 115935, Merck Millipore). For immunostaining, 2.5 µm sections were boiled for 20 min in 10 mM citrate buffer pH 6.0 and incubated with primary antibody anti-F4/80 (2 µg/mL, clone CI: A3, ab6640, Abcam) diluted in PBS with 1.25% BSA overnight at 4 °C, and then incubated with fluorescently labeled secondary antibody (donkey anti-rat IgG Alexa Fluor 488, 5 µg/mL, A-21208, Molecular Probes) for 60 min at 37 °C. Hoechst 33258 nuclear dye (0.5 µg/mL, Sigma Aldrich) was added to the final washing solution. Pictures were captured using a Nikon Eclipse model N *i*-U microscope (Nikon) equipped with Nikon Plan-Fluor objective lenses and an Infinity 2 digital camera (Lumenera Corporation).

Tumor growth assay using B16-F10 *Ctnnb1* knock-out cells in C57BL/6 N mice: On day 11, 48 tumor-bearing animals (primary tumors reaching 20–100 mm^3^) were randomized into 4 groups (*n* = 12), each treated with (i), (ii), (iii), or (v). On day 25, mice were euthanized, and the tumors were dissected, weighed and volumes re-measured with caliper. Three mice from treatment group (i) and two mice from treatment groups (ii), (iii) and (v) were euthanized for ethical reasons before experiment termination, due to skin ulcerations in the tumor area or excessive tumor size. For immunostaining, 2.5 µm frozen sections were incubated with primary antibody anti-CD8 (5 µg/mL, clone 4SM15, eBioscience) diluted in PBS with 1.25% BSA for 60 min at 37 °C and then incubated with secondary antibody (donkey anti-rat IgG Alexa Fluor 488, 5 µg/mL, Thermo Fisher Scientific) for 60 min at 37 °C. Hoechst 33258 nuclear dye (0.5 µg/mL, Sigma Aldrich) was added to the final washing solution. For semi-quantitative analysis, the three areas (high power fields) from each tumor section that contained the highest number of CD8^+^ T cells were selected and the number of T cells were counted in each area. The mean value of the three scored areas in each tumor was calculated and next used in statistical analyses.

Tumor growth assay using B16-F10 cells in B6.129S6-Rag2tm1Fwa N12 mice (Rag2^−/−^)^40^ mice: On day 8, 24 tumor-bearing animals (with primary tumors reaching 18-61mm^3^) were randomized into two groups (*n* = 12), and treatments (i) and (v) were administered to the respective mice. On day 20, the animals were euthanized. One mouse from treatment group (i) and two mice from treatment group (v) were euthanized for ethical reasons before experiment termination, due to skin ulcerations in the tumor area or excessive tumor size.

Tumor growth assay using B16-F10 cells, CD8α depletion and IFNγ neutralization in C57BL/6 N mice: On day 7, 120 tumor-bearing animals (primary tumors reaching 14-45 mm^3^) were randomized into eight groups (*n* = 15): (i) Control diet, (ii) anti-IgG1 (BE0088, Bio X Cell, isotype control for IFNγ), (iii) anti-IgG2 (BE0090, Bio X Cell), isotype control for anti-CD8α), (iv) anti-CD8α (BE0061, Bio X Cell), (v) anti-IFNγ (BE0055, Bio X Cell), (vi) anti-PD-1/G007-LK, (vii) anti-PD-1/G007-LK/anti-CD8α, and (viii) anti-PD-1/G007-LK/anti-IFNγ. 200 µg/mouse of isotype control, depleting and neutralizing antibodies were delivered intraperitoneally on day 7, 10, 13, 17, and 20. Anti-PD-1/G007-LK treatment was initiated on day 10. On day 21, the experiment was terminated, and tumors from euthanized mice were dissected, weighed and volumes re-measured with caliper. 4, 3, 5, 5, 6, 5, 6, and 6 mice from treatment groups (i), (ii), (iii), (iv), (v), (vi), (vii), and (viii), respectively, were found dead or euthanized for ethical reasons during the experiment due to anemia or skin ulcerations in the tumor area. At experiment end, spleens from two animals in group (i), (iii), (iv), and (vii) were dissected and single cells were collected by squeezing the spleen through a cell strainer. Erythrocytes were removed with the Red Blood Cell Lysis Solution (Miltenyi Biotec) and 5 × 10^5^ cells/well were dispensed into 96-well plates. The cells were washed with PBS and stained for living cells for 30 min (FVS780, BD Biosciences). After washing and centrifugation (400 × *g*), the samples were incubated with 50 µl/well of Fc block (anti-mouse CD16/CD32, 1:50, 14-0161-85, clone 93, eBioscience) for 15 min in FACS buffer (PBS with 2% FCS and 0.2% EDTA, 03690, Sigma Aldrich). Thereafter, 50 µl of the following 2X concentrated antibody master mix was added to each well and incubated for 30 min in the dark: BD Horizon™ BUV395 Rat Anti-Mouse CD45 (565967), BV786 Hamster Anti-Mouse CD3e (564379) and BD Horizon™ APC-R700 Rat Anti-Mouse CD8a (564983) (all from BD Biosciences). The cells were fixed and analyzed by flow cytometry using a LSR Fortessa (BD Biosciences) with the gating shown in Supplementary Fig. 16b.

All animal experiment described in this section were performed by ProQinase GmbH, following approval by local animal experiment authorities (Freiburg, Germany) and in compliance with FELASA guidelines and recommendations.

### Multiplex and ELISA immunoassay

Subcutaneous B16-F10 tumors were implanted (left flank, 0.2 × 10^6^ B16-F10 tumor cells suspended in 200 µl [2:1] PBS:matrigel mixture) in 6–8 weeks old female C57BL/6 mice (Taconic). On day 6, animals were distributed into four groups and treatments (i) (*n* = 3), (ii) (*n* = 6), (iii) (*n* = 5), and (v) (*n* = 8) were administered to the mice (see paragraph describing tumor initiation, treatment and analysis of mouse melanoma in vivo). Intraperitoneal injections of anti-PD-1 were administered on day 6, 9, and 13. On day 14, the animals were euthanized and the matrigel-implanted tumors were dissected, treated with 1 mg/mL type IV collagenase and 0.3 mg/mL DNase I (both from Sigma Aldrich) in RPMI and incubated for 45 min at 37 °C. The samples were next centrifuged (1500 × *g*) and the conditioned supernatants collected^39^. The animal experiment was approved by local animal experiment authorities (Norwegian Food Safety Authority, Norway) and in compliance with FELASA guidelines and recommendations. Bio-Plex Pro Mouse Chemokine Panel 33-plex (12002231, Bio-Rad) was used to screen the in vivo tumor conditioned supernatants for cytokine and chemokine expression according to the manufacturer’s protocol. A Bio-Plex handheld magnetic washer (Bio-Rad) was used for the wash steps, and a Luminex-100 instrument with Bio-Plex Manager 4.1 software (Bio-Rad) was used for analysis. Cell supernatants from treated cells and conditioned B16-F10 tumor supernatants (see description for multiplex immunoassay) were assayed using Mouse CCL4/MIP-1 β Quantikine ELISA Kit (MMB00, R&D Systems) according to the manufacturer's protocol.

### Tumor flow cytometry analysis

Primary tumor from treatment groups (i), (ii), (iii), and (v) was collected and processed for flow cytometry analysis to determine the presence of subpopulations of T cells and myeloid-derived suppressor cells (carried out by ProQinase^58,59^). For analysis of T cells and myeloid-derived suppressor cells, animals were treated for 7–17 days to obtain similarly distributed primary tumor volumes ranging from 80–240 mm^3^ (Supplementary Fig. 17a). The animal experiment was performed by ProQinase GmbH, following approval by local animal experiment authorities (Freiburg, Germany) and in compliance with FELASA guidelines and recommendations. Tumors were disrupted using gentleMACS Tubes (Miltenyi Biotec) containing the enzyme mix of the Tumor Dissociation Kit according to the manufacturer’s instructions (Miltenyi Biotech). Erythrocytes were removed with the Red Blood Cell Lysis Solution (Miltenyi Biotech). Single-cell suspensions were counted, and up to 3 × 10^6^ cells/well were dispensed into 96-well plates. The single cells were washed with PBS and stained for living cells (eBioscience^TM^ Fixable Viability Dye eFluor™ 455UV [65-0868-14, eBioscience]). After washing and centrifugation (400 × *g*), the samples were incubated with 50 µl/well of Fc block (anti-mouse CD16/CD32, 1:50, 14-0161-85, clone 93, eBioscience) for 30 min in FACS buffer (PBS with 2% FCS and 0.2% EDTA, 03690, Sigma Aldrich). Thereafter, 50 µl of the following 2X concentrated antibody master mixes were added to each well and incubated for 30 minutes in the dark. For T cells, the following antibodies against murine targets were used: CD45 (CD45-PacBlue [30-F11], 48-0451-82, eBioscience), CD3 (CD3-Violet 605 [17A2], BioLegend), CD4 (CD4-APC-Cy7 [GK1.5], 47-0041-82, eBioscience), CD8a (CD8-PerCP [53-6.7], eBioscience), CD25 (CD25-APC [PC61.5], 17-0251-82, eBioscience) and CD44 (CD44-FITC [IM7], 11-0441-82, eBioscience). For myeloid cells, the following antibodies against murine targets were used: CD45 (CD45-PacBlue [30-F11], 48-0451-82, eBioscience), CD11b (CD11b-FITC [M1/70], 11-0112-82, eBioscience), CD11c (CD11c-APC-Cy7 [N418], 47-0114-80, eBioscience), lymphocyte antigen 6 complex, locus G (Ly6G)(Ly6G-APC [RB6-8C5], 17-5931-81, eBioscience), lymphocyte antigen 6 complex, locus C1 (Ly6C)(Ly6C-PE [HK1.4], 12-5932-82, eBioscience) and CD103 (CD103 [Integrin alpha E], PerCP-Cy5.5 [2E7], 121415, BioLegend). The cells were washed and fixed. Cells stained with the T cell panel markers were prepared for intracellular staining by adding 50 µl fixation/permeabilization (1:3) buffer (00-5523-00, eBioscience) for 30 min. Thereafter, 100 µl 1X permeabilization buffer (00-5523-00, eBioscience) was added and the cells were centrifuged at 400 × *g*. The cell pellet was resuspended in 1X permeabilization buffer containing anti- forkhead box P3 (FoxP3) antibody (FoxP3-PE [FJK-16s], 12-5773-82, eBioscience) and incubated for 30 minutes in the dark. After washing twice with permeabilization buffer, the cells were washed with FACS buffer and kept at 4 °C in the dark until analysis. The samples were analyzed by flow cytometry using an LSR Fortessa (BD Biosciences) and the gating strategy is shown in Supplementary Fig. 18a, b.

### In vitro T-cell assays

Eight weeks old, female and wild-type C57BL/6 and C57BL/6-Tg(TcraTcrb)1100Mjb/J (OT-I) mice, harboring major histocompatibility complex (MHC) class I (H2-K^b^)-restricted and ovalbumin-specific CD8^+^ T cells, were obtained from Jackson Laboratories. For the T cell proliferation and cytokine release-assays, spleen and lymph nodes were harvested from OT-1 mice and CD8^+^ T cells were sorted by using the Miltenyi CD8a^+^ T Cell Isolation Kit (130-104-075). Splenocytes from wild-type C57BL/6 mice were irradiated (25 Gy) and used as antigen-presenting cells. Antigen-presenting cells and CD8^+^ T cells were mixed at a 4:1 ratio with 100,000 APCs and 25,000 T cells per well in 96-well plates. For activation of OT-1 CD8^+^ T cells, 4 µg/ml of synthetic SIINFEKL peptide (AnaSpec) was added. For polyclonal T cell activation, Concanavalin A (5 µg/ml, Sigma Aldrich) or immobilized CD3 (0.5 µg/ml)/CD28 (5 µg/ml) antibodies (clones 145-2c11 and 37.51, BioXCell) were used. To evaluate the effect of G007-LK on T cell proliferation and cytokine production, 1 µM G007-LK or vehicle control (0.01% DMSO) was added to all conditions. For proliferation assays, ^3^H-Thymidine (Montebello Diagnostics, Oslo, Norway) was added after 48 or 72 h of treatment. Cells were harvested after 24 additional hours and radiolabeling was counted with a MicroBeta plate reader. Supernatant was harvested from the plates after 72 h of treatment. IL2 and IFNγ quantitation in culture supernatants was determined using ELISA Max Deluxe kits (431004 and 430804, Biolegend). For flow cytometric analysis of surface markers on APC/ SIINFEKL or ConA-activated CD8^+^ T cells, the cells were pooled from 8–12 wells from the setup described previously and the following antibodies were used: Anti-CD62L (1705-09 L, Southern biotech), anti-CD69 (1715-02, Southern biotech), anti-CD8a (553036, BD Biosciences), anti-CD25 (17-0251-82, eBioscience), anti-CD44 (103049, Biolegend), and anti-CD3e (35-0031, TONBO Biosciences). For intracellular staining, the cells were incubated for 4 h in protein-transport inhibitor before permeabilization and fixation, according to the manufactures’ protocol (Fix/Perm kit with Golgistop 554715, BD Biosciences) and then stained with anti-granzyme B (12-8898-82, eBioscience). All antibodies were used at a working concentration of 2 µg/ml. The samples were analyzed using the Attune NxT flow cytometer (Thermo Fisher Scientific) and Flow Jo software (BD Biosciences).

### RNA sequencing

The following samples were used for RNA sequencing experiments and RNA was isolated using GenEluteTM Mammalian Total RNA Miniprep Kit (Sigma Aldrich): Three biological replicates of cultured B16-F10 cells, treated with DMSO (0.01%), G007-LK (1 µM), WNT3a (0.5 µg/mL) or G007-LK and WNT3a for 24 h. And in addition, three pooled technical replicates from a panel of 18 human melanoma cell lines treated with DMSO (0.01%) or G007-LK (1 µM) for 24 hours: The cell lines SK-MEL-28, MeWo, and A-375 were obtained from the American Type Culture Collection (ATCC). WM35, WM115, WM1341B, WM1366, WM983B, WM451Lu, WM239A, WM266.4, WM852, WM1382, WM9, WM793B were obtained from the Wistar Institute. LOX-IMVI, FEMX-I and FEMX-V were established at the Norwegian Radium Hospital (Oslo, Norway). Cell line authentication was performed by short tandem repeat profiling and subsequent analysis at the Norwegian Radium Hospital. RNA sequencing (TruSeq Stranded mRNA kit for library prep and NextSeq500 v2 chemistry used for sequencing, both Illumina Inc.) was performed at the Genomics Core Facility Oslo (Oslo University Hospital, Norway).

### DNA sequencing

Kinome targeted re-sequencing of the 18 human melanoma cell lines was performed using the SureSelect Human Kinome kit (Agilent Technologies), with capture probes targeting 3.2 Mb of the human genome, including exons and untranslated regions (UTRs) of all known kinases and selected cancer‐related genes (to a total of 612 genes). Library construction and in solution capturing was performed following Agilent’s SureSelectXT library construction kit and SureSelect Target enrichment protocol, respectively. Sequencing was performed on an Illumina HiSeq2500 using the TruSeq SBS Kit v5 generating paired‐end reads of 75 bp in length. Base calling, de-multiplexing and quality filtering was performed using Illumina’s software packages SCS2.8/RTA1.8 and Off‐line Basecaller‐v1.8.

### Bioinformatics

Transcripts were quantified with kallisto (v0.44)^60^ using ensembl transcriptome release 91 for human (GRCh38) and release 92 for mouse (GRCm38)^61^. Ensemble BioMart was used to map human orthologs in mouse^60^. Differentially expressed genes (DEGs) were identified with sleuth (v0.29)^62^, limma (3.34.9)^63^ (did not result in any comparisons with adjusted *P* values < 0.05) and DESeq2^64^ in the R programming environment (The R Project for Statistical Computing). The R-package NMF (0.23.6)^65^ was used to make hierarchical clusters with TPM values as input. For detection of probable driver mutations, the RNAseq data was aligned with HISAT2 (v2.1.0)^66^ before VarDict (v1.2)^67^ restricted to SNVs reported more than once in COSMIC (v82)^68^ was applied. Due to the variable coverage in RNAseq data, additional SNVs found in an external unpublished gene panel sequencing experiment for the same cell lines, were included. Variants found in both datasets were annotated using ANNOVAR (2017-07-17)^69^. The data analysis was performed by the Bioinformatics Core Facility (Oslo University Hospital, Norway). DEGs analysis data, including log2-fold change and adjusted p-values, were uploaded into Ingenuity Pathway Analysis (IPA) version 01-10 (Qiagen). The DEGs analysis data were analyzed using the core analysis function with the Ingenuity Knowledge Base (genes only) reference set and direct relationships, with no filters set for node types, data sources, confidence, species, tissues, and cell lines and mutations. For the IPA core analyses, the log2-fold and or adjusted p-value cutoffs are specified in the figure legends.

### Statistics and reproducibility

No sample size calculation was performed. Sample sizes for both in vivo and in vitro experiments were determined based on experiment experience, pilots and preliminary experiments as well as what was reported in the literature. Samples sizes for each experiment and numbers of independent repeats are indicated in figure legends. All in vivo experiments included contain ≥8 independent biological replicates, except for Fig. 4a, b (≥3) and Fig. 5e (≥3). For all in vitro assays, all attempts at replication were successful through repeated experiments (two or more replications). Sigma Plot^®^ 12.5 (Systat Software Inc.) was used to perform statistical tests: Student’s *t*-test for comparisons with homogeneous variances (Shapiro-Wilk test, *P* > 0.05) and Mann-Whitney rank sum tests for comparisons where the normality assumption was violated (Shapiro-Wilk test, *P* < 0.05). GrapPad Prism 7 was used for Kaplan-Meyer estimations and statistical analysis. Single outlier detections were identified by Dixon’s and/or Grubb’s tests (threshold, *P* < 0.05) using ControlFreak (Contchart software).

### Reporting Summary

Further information on research design is available in the Nature Research Reporting Summary linked to this article.

## Supplementary information

Supplementary Information

Description of Additional Supplementary Files

Supplementary Data 1

Reporting Summary

## Data Availability

The RNA sequencing data for this study are available from ArrayExpress with accession numbers E-MTAB-8438 (human cell lines) and E-MTAB-8101 (B16-F10 mouse cell line). For the mouse experiment, the data are deposited both as raw fastq files and processed as RNA abundance counts. For the human RNA experiment only abundance counts are deposited. Somatic mutations from DNA sequencing are available at https://github.com/ous-uio-bioinfo-core/waaler-et-al-2020; 10.5281/zenodo.3703045. Fastq files for the human sequencing experiments and additional data generated and analyzed in this study are available from the corresponding author upon request. The source data underlying plots shown in main figures are provided in Supplementary Data 1. Additional data generated and analyzed in this study are available from the corresponding author upon request.

## Code availability

Custom scripts used to process and analyze the sequencing data and to make most of the related figures and tables are available at 10.5281/zenodo.3703045.
