# Supplementary material for: Tankyrase inhibition sensitizes melanoma to PD-1 immune checkpoint blockade in syngeneic mouse models
Source: Commun Biol. 2020 Apr 24;3:196. doi: 10.1038/s42003-020-0916-2 (PMC7181813; doi:10.1038/s42003-020-0916-2)
Supplement: Supplementary file 1 — Supplementary Information [file 42003_2020_916_MOESM1_ESM.pdf]

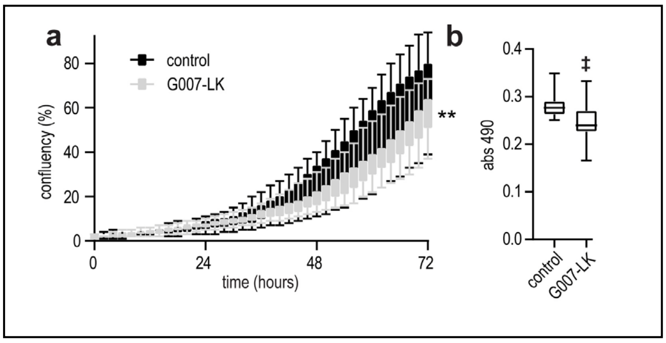

Supplementary Fig. 1 G007-LK-mediated tankyrase inhibition moderately decreases cell growth of B16-F10 cells *in vitro*. a, Real-time confluency (%) measurements of proliferating B16-F10 cells treated with vehicle (DMSO, 0.01%, black) or G007-LK (1 μM, grey). Boxplot graph show first and third quartiles and maximum and minimum whiskers. For **a** and **b**: One representative experiment of three repeated assays with 30 replicates for each treatment group is shown. Two-tailed t-tests are indicated by ** (*P* < 0.01) and Mann-Whitney rank sum test is indicated by ‡ (*P* < 0.01). **b**, End point MTS assay (Abs_{490}). Boxplot shows median, first and third quartiles and maximum and minimum whiskers.

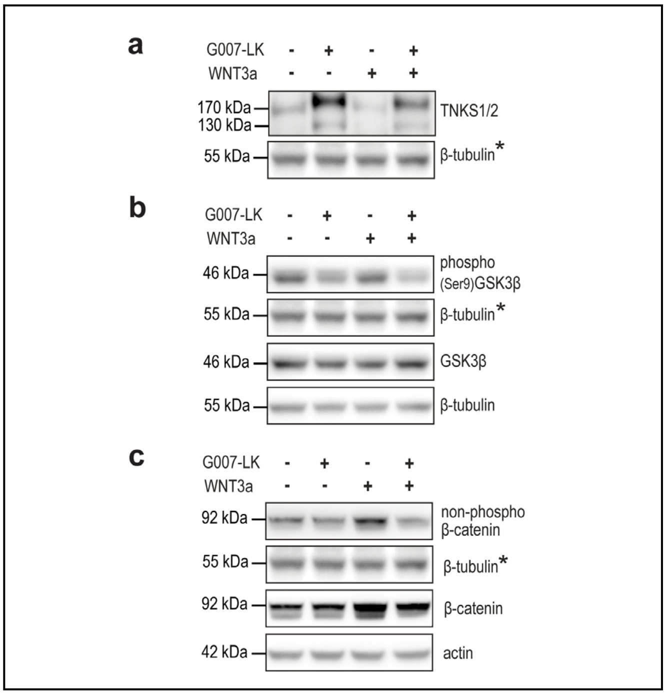

Supplementary Fig. 2 G007-LK can reduce WNT/β-catenin signaling in B16-F10 cells *in vitro*. a, Representative immunoblots of cytoplasmic TNKS1/2. Treatments used for cultured B16-F10 cells in **a**-**c**: Vehicle (DMSO, 0.01%), G007-LK (1 μM), recombinant WNT3a or WNT3a + G007-LK for 24 hours. For **a**-**c**: Actin or β-tubulin document equal protein loading and * indicates that the same β-tubulin immunoblot is used as loading control for both TNKS1/2, (phospho[ser9]GSK3β) and β-catenin (non-phospho, Ser33/37/Thr41). **b**, Representative immunoblots cytoplasmic of inactive form of GSK3β (phospho[ser9]GSK3β) and total GSK3β. **c**, Representative immunoblots cytoplasmic active form of β-catenin (non-phospho Ser33/37/Thr41) and total β-catenin.

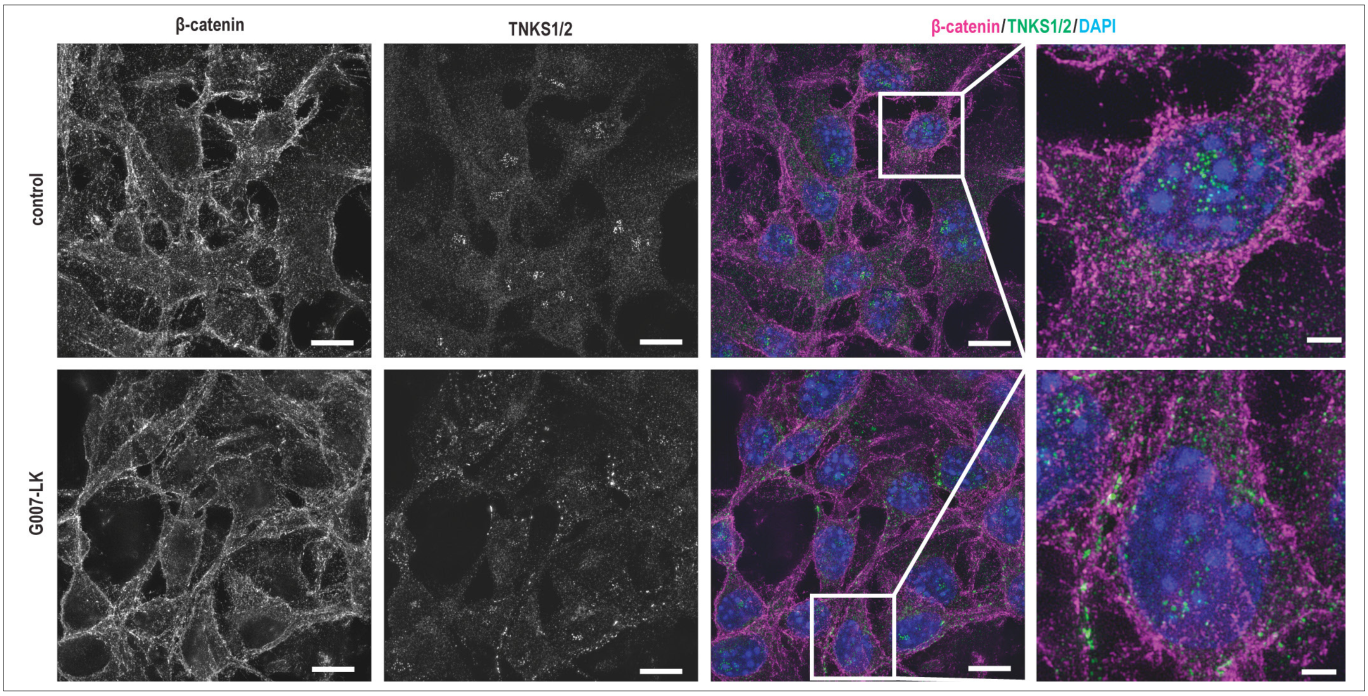

Supplementary Fig. 3 G007-LK treatment induces the formation of TNKS1/2-containing puncta in B16-F10 cells *in vitro*. Co-staining using antibodies against β-catenin (pink) and TNKS1/2 (green) along with nuclear DAPI staining (blue) upon vehicle control (0.01% DMSO) and G007-LK (1 µM) treatment (24 hours) of B16-F10 cells (right panels). Structured illumination microscopy (SIM) images of G007-LK-treated cells show unchanged intensity of membrane β-catenin but formation of cytoplasmic TNKS1/2-containing puncta indicating accumulation of β-catenin destruction complexes. Scale bar = 10 µm. Zoom scale bar = 2 µm.

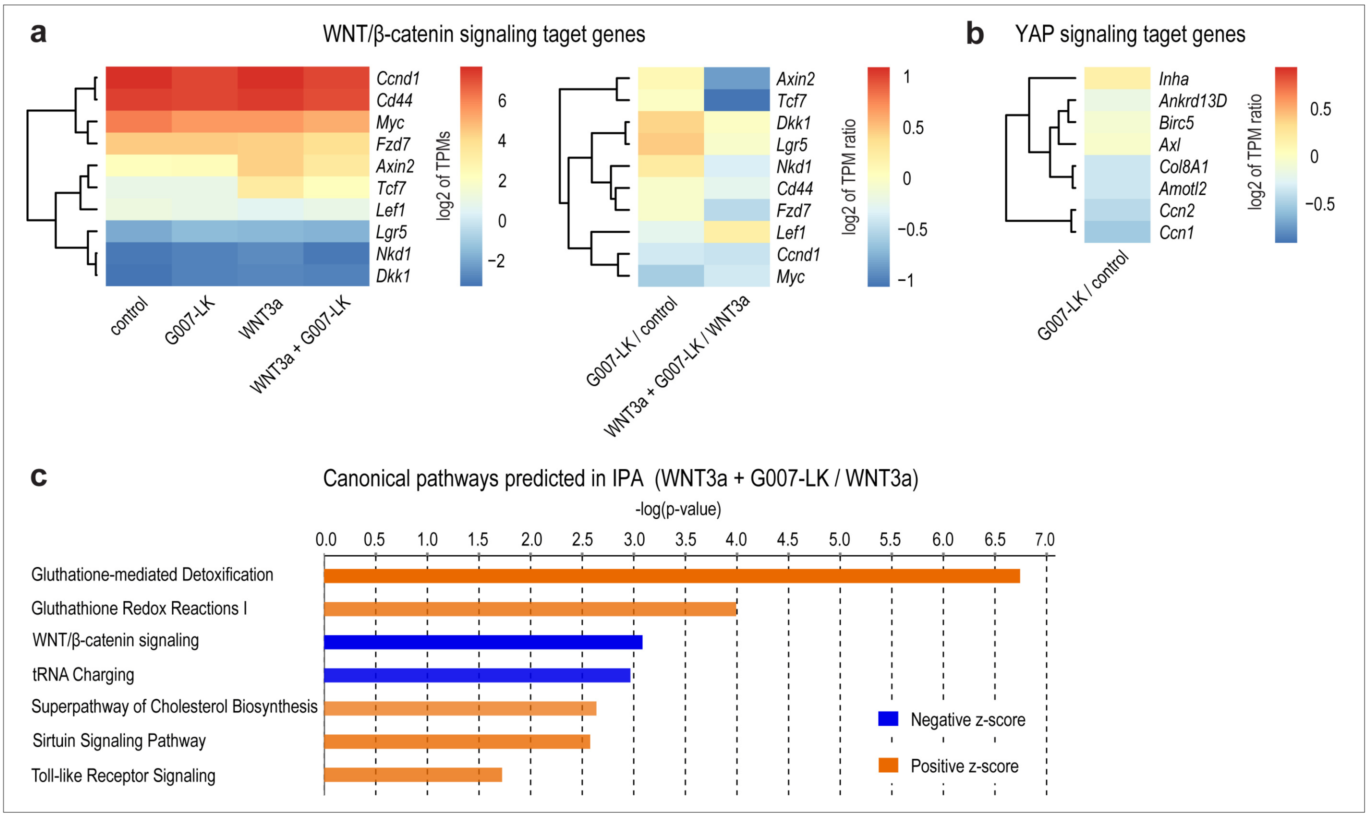

Supplementary Fig. 4 G007-LK treatment antagonizes WNT/β-catenin and YAP signaling in B16-F10 cells *in vitro*. **a**, Heatmap and clustering of transcribed WNT/β-catenin signaling target genes. Left panel shows log2-transformed transcripts per millions (TPM). Right panel shows the TPM ratios (log2) for G007-LK versus vehicle control (0.01% DMSO) and G007-LK + WNT3a versus WNT3a treatments. The induction of *Axin2* and *Tcf7* was in particular responding to WNT3a-mediated pathway activation and subsequently counteracted by G007-LK treatment. For **a**-**c**: RNA sequencing data obtained from 3 independent experiments. Treatments used for 24 hours: Vehicle control (DMSO, 0.01%) and G007-LK (1 μM) in **a** and **b**, recombinant WNT3a and WNT3a + G007-LK in **a**. **b**, Heatmap and clustering of transcribed YAP signaling target genes: TPM ratios (log2) for G007-LK versus control (DMSO, 0.01%). **c**, IPA core analysis of canonical pathways affected when comparing transcription in the G007-LK + WNT3a versus WNT3a treatments. Differentially expressed genes with a corrected *P* value of <0.1 were analyzed for direct relationships. The plot displays the seven top canonical pathways, including WNT/β-catenin signaling, identified with core analysis with a –log10(*P* value) >1.3 and a z-score of >2 or <-2. Positive z-score (orange) indicates pathway activation whereas negative z-score (blue bars) indicates pathway inhibition.

a

Ctnnb1 exon 4

Coding sequence
ATATTGACGGGCAGTATGCAATGACTAGGGCTCAGAGGGTCCGAGCTGCCATGTTCCCT
GAGACGCTAGATGAGGGCATGCAGATCCCATCCACGCAGTTTGACGCTGCTCATCCCAC
TAATGTCCAGCGCTTGGCTGAACCATCACAGATGTTGAAACATGCAGTTGTCAATTTGAT
TAACTATCAGGATGACGCGGAACTTGCCACACGTGCAATTCCTGAGCTGACAAAACTGC
TAAACGATGAGGACCAG

Amino acid sequence
IDGQYAMTRAQRVRAAMFPETLDEGMQIPSTQFDAAHPTNVQRLAEPSQMLKHAVVNLINY
QD**DAELATRAIPELTKLLNDEDQ**

Ctnnb1 KO1

Allele #1, single base loss > frameshift and premature stop:
TATCAGGAT-ACGCGGAACTTGCCACACGTGCAATTCCT
Amino acid sequence
IDGQYAMTRAQRVRAAMFPETLDEGMQIPSTQFDAAHPTNVQRLAEPSQMLKHAVVNLINY
QD**TRNLPHVQFLS***

Allele #2, Insertion G - frameshift and premature stop:
TCAATTTGATTAACTATCAGGATggACGCGGA
Amino acid sequence
IDGQYAMTRAQRVRAAMFPETLDEGMQIPSTQFDAAHPTNVQRLAEPSQMLKHAVVNLINY
QD**GRGTCHTCNS***

Ctnnb1 KO2

Allele #1
tg > c- > frameshift and premature stop:
TCAGGAc-ACGCGGAACTTGC
Amino acid sequence
IDGQYAMTRAQRVRAAMFPETLDEGMQIPSTQFDAAHPTNVQRLAEPSQMLKHAVVNLINY
QD**TRNLPHVQFLS***

Allele #2
tg > a- frameshift and premature stop:
TAACTATCAGGAA-AC
Amino acid sequence
IDGQYAMTRAQRVRAAMFPETLDEGMQIPSTQFDAAHPTNVQRLAEPSQMLKHAVVNLINY
Q**ETRNLPHVQFLS***

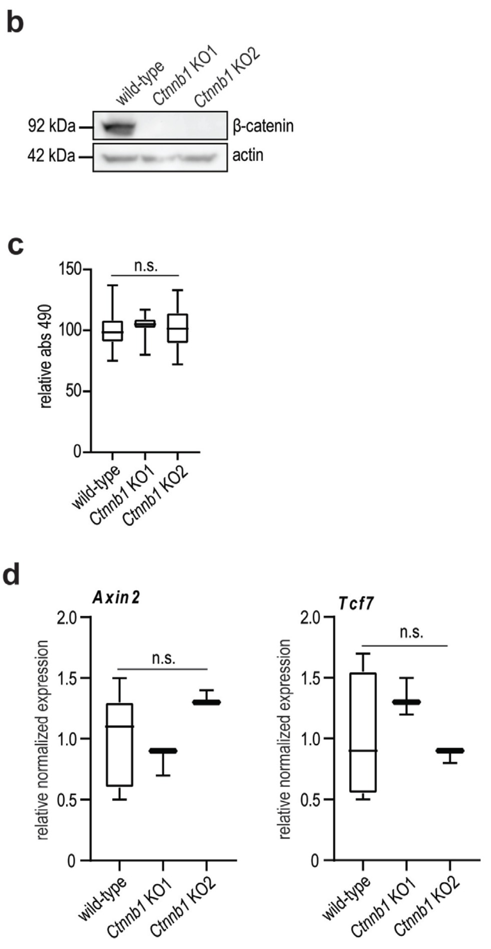

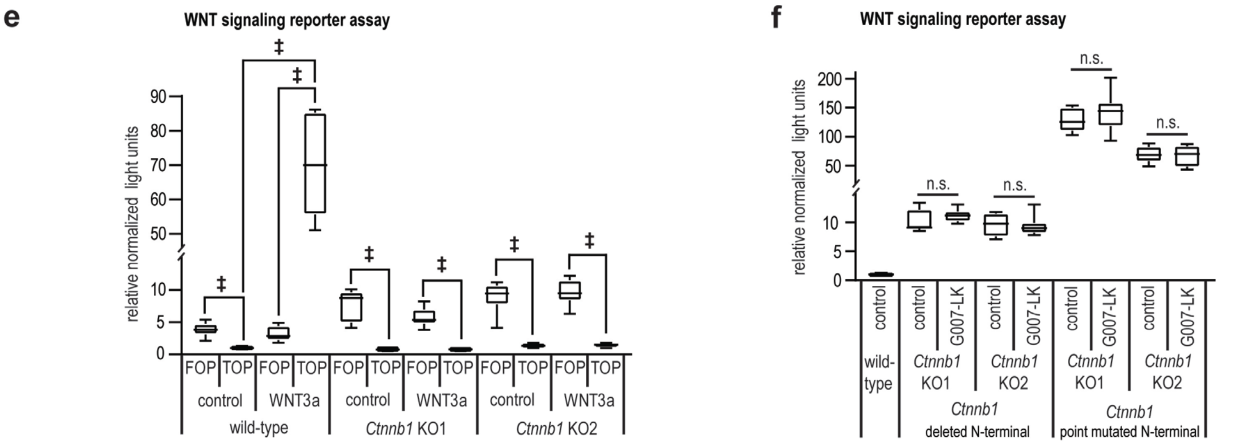

Supplementary Fig. 5 CRISPR/Cas9-based knock-out of *Ctnnb1* in B16-F10 cells. a, Coding (upper) and amino acid (lower) sequences for wild-type *Ctnnb1* exon 4. Alterations in coding and amino acid (highlighted in blue) sequences in allele 1 and 2 upon CRISPR-Cas9-based gene editing are shown for B16-F10$^{Ctnnb1\text{KO}}$ clone 1 and 2 (*Ctnnb1* KO1 and 2). **b**, Immunoblots from total lysates showing loss of β-catenin in B16-F10$^{Ctnnb1\text{KO}}$ cell lines compared to wild-type B16-F10 cells. Actin is used as loading control. **c**, End point MTS assay (Abs_{490}) for proliferating B16-F10 wild-type and B16-F10$^{Ctnnb1\text{KO}}$ cell lines. One representative experiment from two repeated assays with 9 replicates is shown. n.s. = not significant. For **c**-**f**: Boxplots show median, first and third quartiles and maximum and minimum whiskers. **d**, Real-time RT-qPCR analyses of B16-F10$^{Ctnnb1\text{KO}}$ cell lines show comparable expression of WNT/β-catenin signaling target genes (*Axin2* and *Tcf7*) compared to wild-type B16-F10 cells. Combined data from two independent experiments with three replicates each are shown. n.s. = not significant. **e**, Luciferase-based reporter assay for measuring WNT/β-catenin signaling activity. B16-F10 and B16-F10$^{Ctnnb1\text{KO}}$ cell lines transiently transfected with superTOPflash (vector with TCF promoter binding sites) or FOPflash (control vector with mutated TCF binding sites) along with *Renilla* luciferase (for normalization). All samples are normalized to superTOPflash signal of wild-type control. WNT3a induced superTOPflash reporter activity only in wild-type B16-F10 cells. The result shows that absence of β-catenin disables WNT signaling reporter activation in B16-F10$^{Ctnnb1\text{KO}}$ cell lines. Background FOPflash activities were significantly higher than SuperTOPflash activities for all treatments indicating low basal WNT/β-catenin signaling activity in cultured B16-F10 cells. Mann-Whitney rank sum tests are indicated by ‡ ($P < 0.01$). Representative experiment with 8 replicates for each treatment group is shown. **f**, Luciferase-based reporter assay for measuring WNT/β-catenin signaling activity in B16-F10$^{Ctnnb1\text{KO}}$ cell lines transiently transfected with superTOPflash, *Renilla* luciferase and vectors expressing either β-catenin with deleted or point mutated N-terminal domain containing GSK3β and casein kinase 1 alpha (CK1α) phosphorylation sites. Co-transfected cells were treated with G007-LK or vehicle control (0.01% DMSO) for 24 hours. All samples are normalized to superTOPflash signal of wild-type control. n.s. = not significant. Representative experiment with 5-8 replicates for each treatment group is shown. G007-LK treatment was unable to counteract reporter activity induced by overexpression of mutated versions of β-catenin.

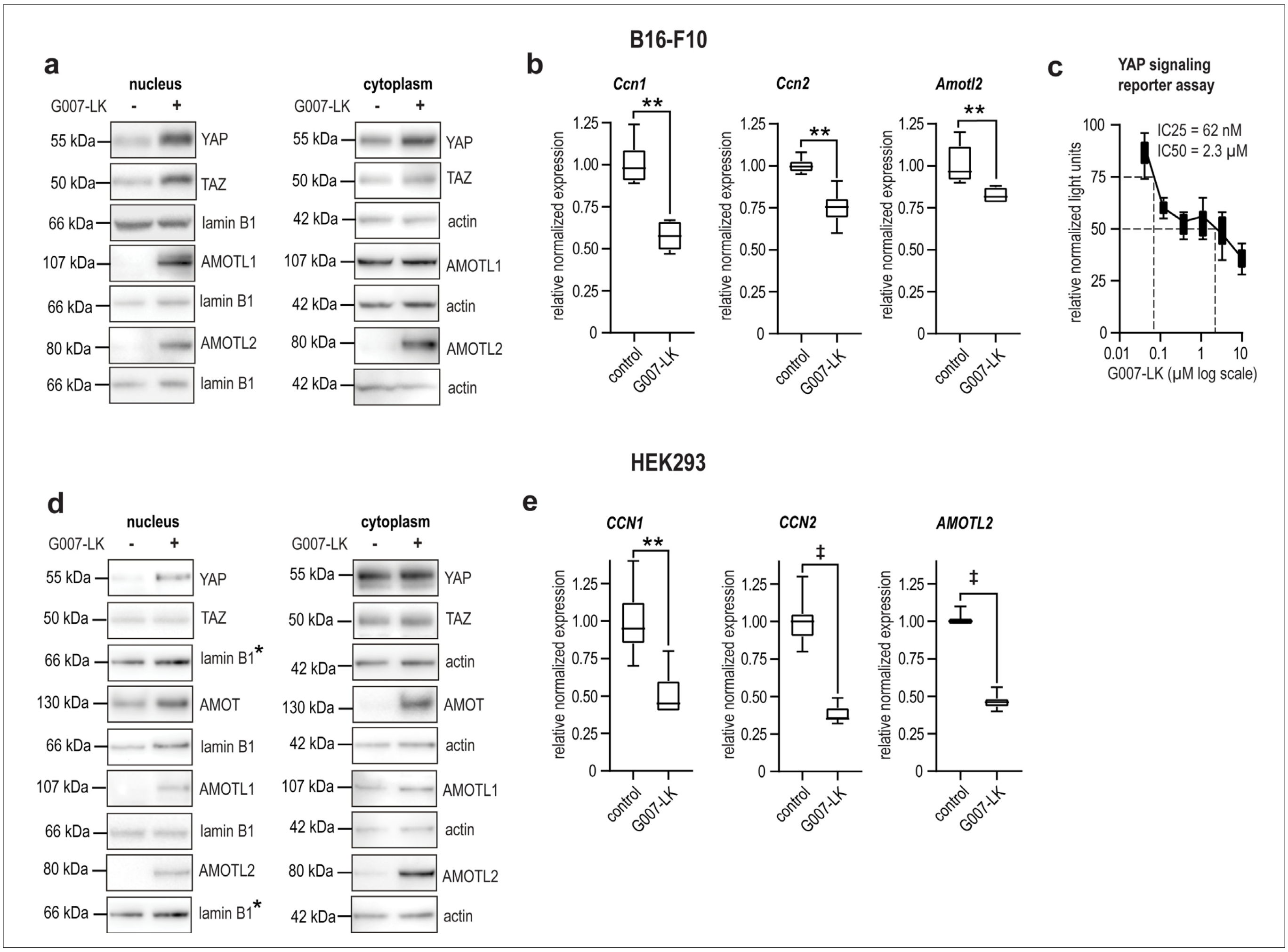

Supplementary Fig. 6 G007-LK can stabilize AMOT proteins and reduce YAP signaling transcriptional activity in B16-F10 cells *in vitro*. a, Representative immunoblots from cultured B16-F10 cells. For **a** and **d**: Nuclear (left panel) and cytoplasmic (right panel) YAP, TAZ, AMOTL1 and AMOTL2. Lamin B1 and actin document equal protein loading. Treatments used in **a**, **b**, **d** and **e**: Vehicle (DMSO, 0.01%) or G007-LK (1 μM) for 24 hours. **b**, Real-time RT-qPCR analyses from cultured B16-F10 cells. For **b** and **e**: YAP signaling target genes (*Ccn1*, *Ccn2* and *Amotl2*). One-tailed Mann-Whitney rank sum tests are indicated by ‡ (*P* < 0.01) and one-tailed t-tests are indicated by ** (*P* < 0.01). Boxplots show median, first and third quartiles and maximum and minimum whiskers. Combined data from three independent experiments with three replicates each are shown. **c**, B16-F10 cells transiently transfected with a luciferase-based reporter assay to measure YAP signaling activity (8XGTIIC-luciferase) and *Renilla* luciferase followed by treatment for 24 hours with vehicle control (DMSO, 0.01%) or various doses of G007-LK. Boxplot graph show first and third quartiles and maximum and minimum whiskers of combined data from two independent experiments with four replicates each. The result shows a dose-dependent reduction in reporter activity upon G007-LK treatment. IC_{25}-value = 62 nM. IC_{50}-value = 2.3 μM. **d**, Representative immunoblots from cultured HEK293 cells. * indicates that the same lamin B1 immunoblot is used as loading control for YAP, TAZ and AMOTL2. **e**, Real-time RT-qPCR analyses from cultured HEK293 cells.

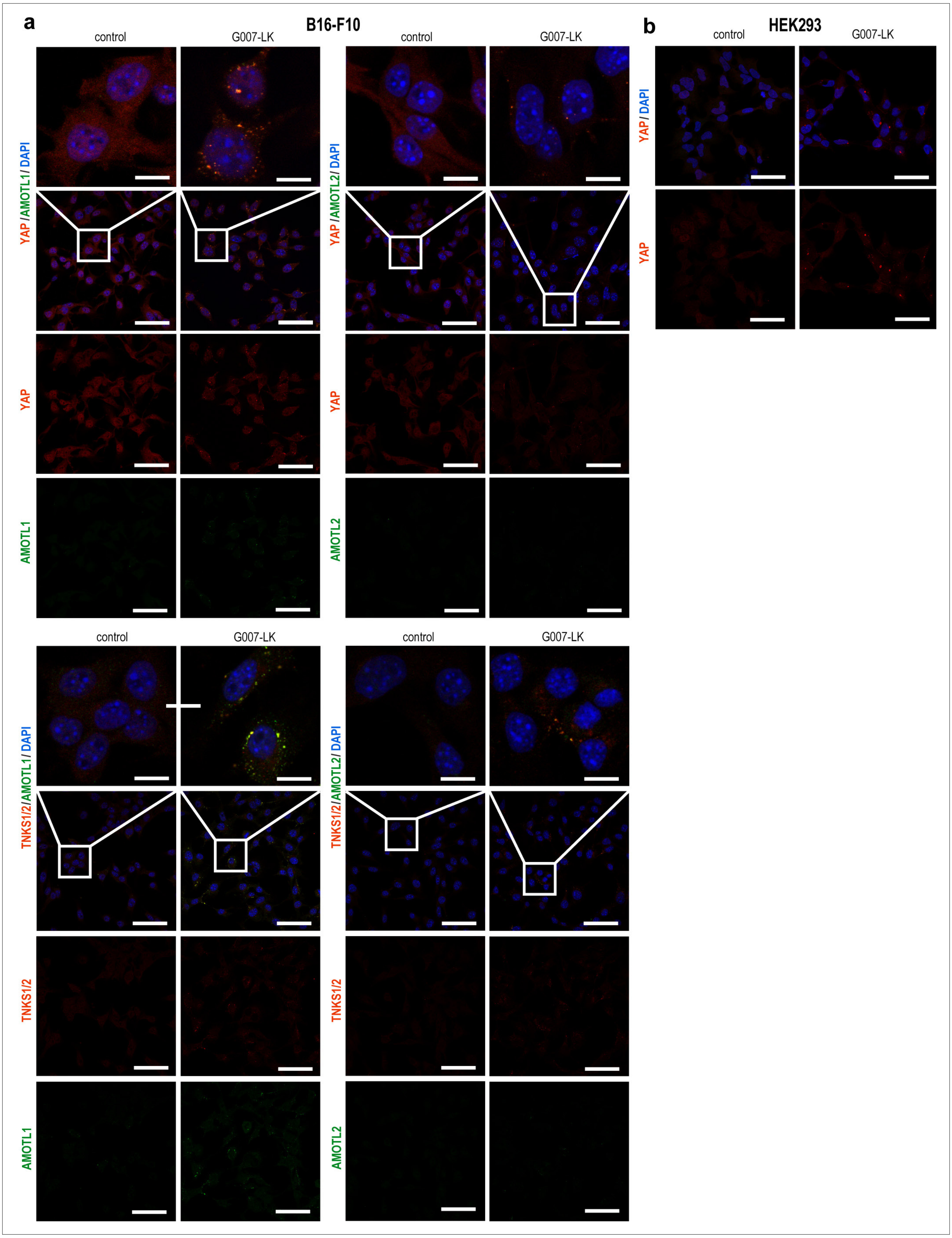
a
B16-F10
control
G007-LK
control
G007-LK
YAP/AMOTL1/DAPI
YAP
AMOTL1
YAP/AMOTL2/DAPI
YAP
AMOTL2
TNKS1/2/AMOTL1/DAPI
TNKS1/2
AMOTL1
TNKS1/2/AMOTL2/DAPI
TNKS1/2
AMOTL2
b
HEK293
control
G007-LK
YAP/DAPI
YAP

Supplementary Fig. 7 G007-LK treatment induces the formation of puncta containing YAP, AMOT proteins and TNKS1/2 in B16-F10 cells *in vitro.* a, Co-staining using antibodies against AMOTL1 (green, left panels), AMOTL2 (green, right panels), YAP (red, upper panels) and TNKS1/2 (red, lower panels) along with nuclear DAPI staining (blue) upon treatment of B16-F10 cells. The confocal images of G007-LK-treated cells show the formation of puncta, predominantly in the cytoplasma, with co-localized AMOTL1-YAP, AMOTL2-YAP (upper panels), AMOTL1-TNKS1/2 and AMOTL2-TNKS1/2 (lower panels). For **a** and **b**: Vehicle control (0.01% DMSO) and G007-LK (1 μM) were used as treatment (24 hours). Scale bar = 50 μm. Zoom scale bar = 10 μm. **b,** Co-staining using antibodies against YAP (red) and nuclear DAPI staining (blue) upon treatment of HEK293 cells. Large YAP-containing puncta are forming primarily adjacent to the nuclei upon G007-LK treatment.

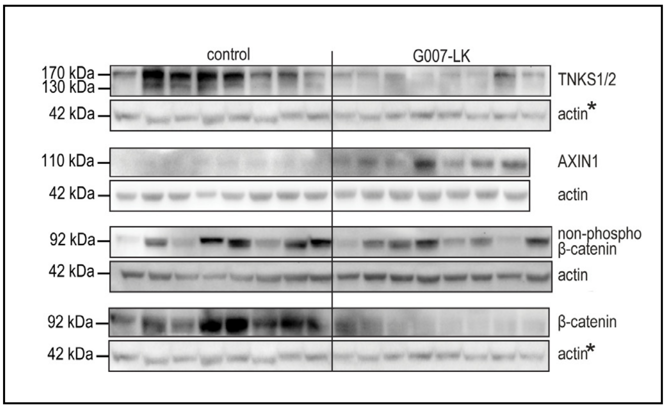

Supplementary Fig. 8 G007-LK can affect markers for WNT/β-catenin signaling in B16-F10 tumors in C57BL/6N mice. Representative immunoblots of extracts from whole s.c. B16-F10 tumors showing altered expression of TNKS1/2, AXIN1, active form of β-catenin (non-phospho, Ser33/37/Thr41) and β-catenin (total) after mice were treated for 4 days with G007-LK diet (*n* = 8, *n* = 7 for AXIN1) or a control diet (*n* = 8). Actin documents equal protein loading and * indicates that the same actin immunoblot is used as loading control for both TNKS1/2 and β-catenin.

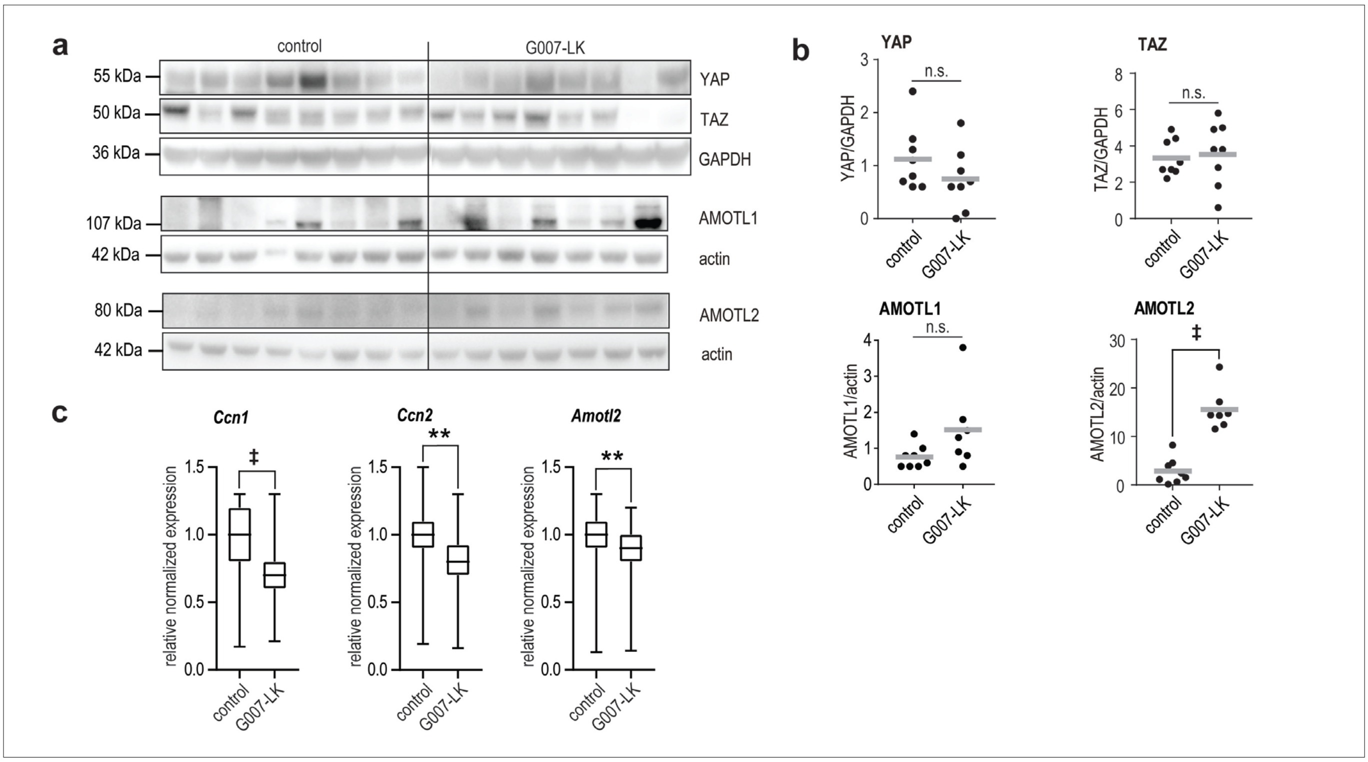

Supplementary Fig. 9 G007-LK can reduce YAP signaling transcriptional activity in B16-F10 tumors in C57BL/6N mice. a, Representative immunoblots from whole (s.c.) B16-F10 tumors showing altered expression of YAP, TAZ, AMOTL1 and AMOTL2. Actin documents equal protein loading. For **a**, **b** and **c**: Upon 4 days of treatment with G007-LK diet (*n* = 8, *n* = 7 for AMOTL1 and AMOTL2) compared to controls (*n* = 8). One-tailed Mann-Whitney rank sum tests are indicated by ‡ (*P* < 0.01) and one-tailed t-tests are indicated by ** (*P* < 0.01) and n.s. = not statistically significant. **b**, Quantified ratios (protein vs. actin loading control) from **a**. Mean values are indicated by grey lines. **c**, Real-time RT-qPCR analyses of hippo signaling target genes (*Ccn1*,*Ccn2* and *Amotl2*). Boxplots show median, first and third quartiles and maximum and minimum whiskers for combined data from 3 independent experiments with three replicates.

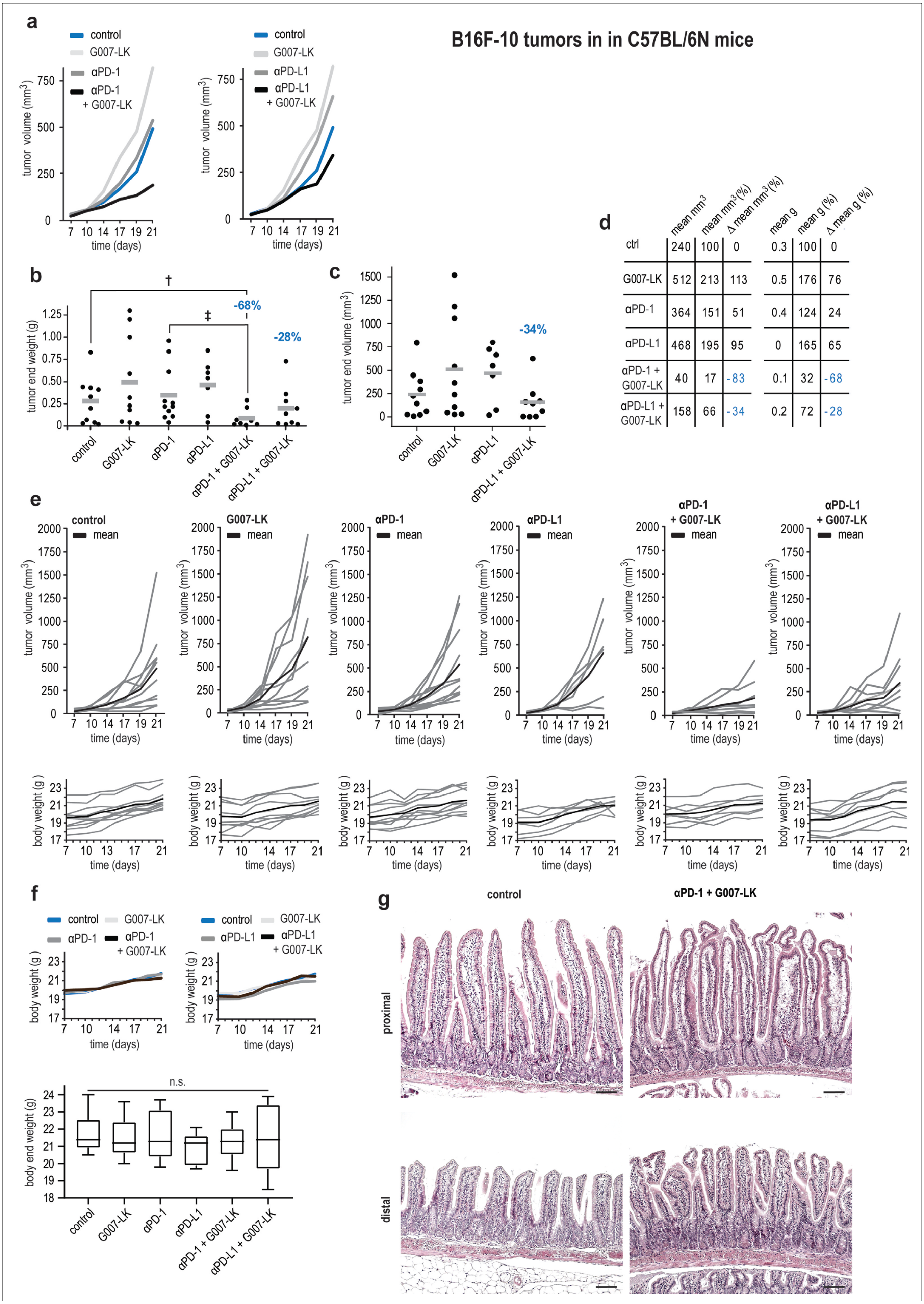

	mean mm³	mean mm³ (%)	Δ mean mm³ (%)	mean g	mean g (%)	Δ mean g (%)
ctrl	240	100	0	0.3	100	0
G007-LK	512	213	113	0.5	176	76
αPD-1	364	151	51	0.4	124	24
αPD-L1	468	195	95	0	165	65
αPD-1 + G007-LK	40	17	-83	0.1	32	-68
αPD-L1 + G007-LK	158	66	-34	0.2	72	-28

Supplementary Fig. 10 Dual inhibition of tankyrase and PD-1 confers synergistic anti-tumor efficacy in B16-F10 tumors in C57BL/6N mice. a, Mean s.c. B16-F10 tumor volumes (mm^3). For **a** and **f**: Control diet (blue), G007-LK diet (light grey, left panel), anti-PD-1 (grey, left panel) and anti-PD-1/G007-LK (black, left panel), anti-PD-L1 (grey, right panel) and anti-PD-L1/G007-LK (black, right panel). For **a**-**f**: Mice treated from day 10 until day 21. Treatments: Control diet (*n* = 10), G007-LK diet (*n* = 10), anti-PD-1 (*n* = 11), anti-PD-L1 (*n* = 7), anti-PD-1/G007-LK (*n* = 8) and anti-PD-L1/G007-LK (*n* = 9). **b**, Tumor end weight reduction upon anti-PD-1/G007-LK (-68%) or anti-PD-L1/G007-LK (-28%) treatment compared to control. Mann-Whitney rank sum tests are indicated by † ($P < 0.05$) and ‡ ($P < 0.01$). **c**, Tumor end volume reduction upon anti-PD-L1/G007-LK (-34%) treatment compared to control. **d**, Left table depicts mean tumor end volumes (mm^3), mean relative tumor end volumes (mm^3 [%]) and relative differences from control (Δmm^3 [%]). Right table depicts mean tumor end weights (g), mean relative tumor end weights (g [%]) and relative differences from control (Δg [%]). **e**, Single s.c. B16-F10 tumor volumes (upper panels) and body weights (lower panels). Mean values are shown in black. **f**, No differences in mean body weights in mice treated from day 10 until day 21 (upper panels) and body end weight (lower panel). Boxplot shows median, first and third quartiles and maximum and minimum whiskers. n.s = not significant. **g**, A histopathological examination performed at experiment termination documented no abnormalities in morphology, proliferation or differentiation in the small intestinal mucosa upon combined anti-PD-1/G007-LK treatment. Representative pictures from multiple sections of H&E-stained proximal (upper panels) and distal (lower panels) parts of the small intestine from mice treated with control diet (left panels, *n* = 6) or anti-PD-1/G007-LK treatment (right panels, *n* = 6). Scale bars: 100 μm (original magnification ×100).

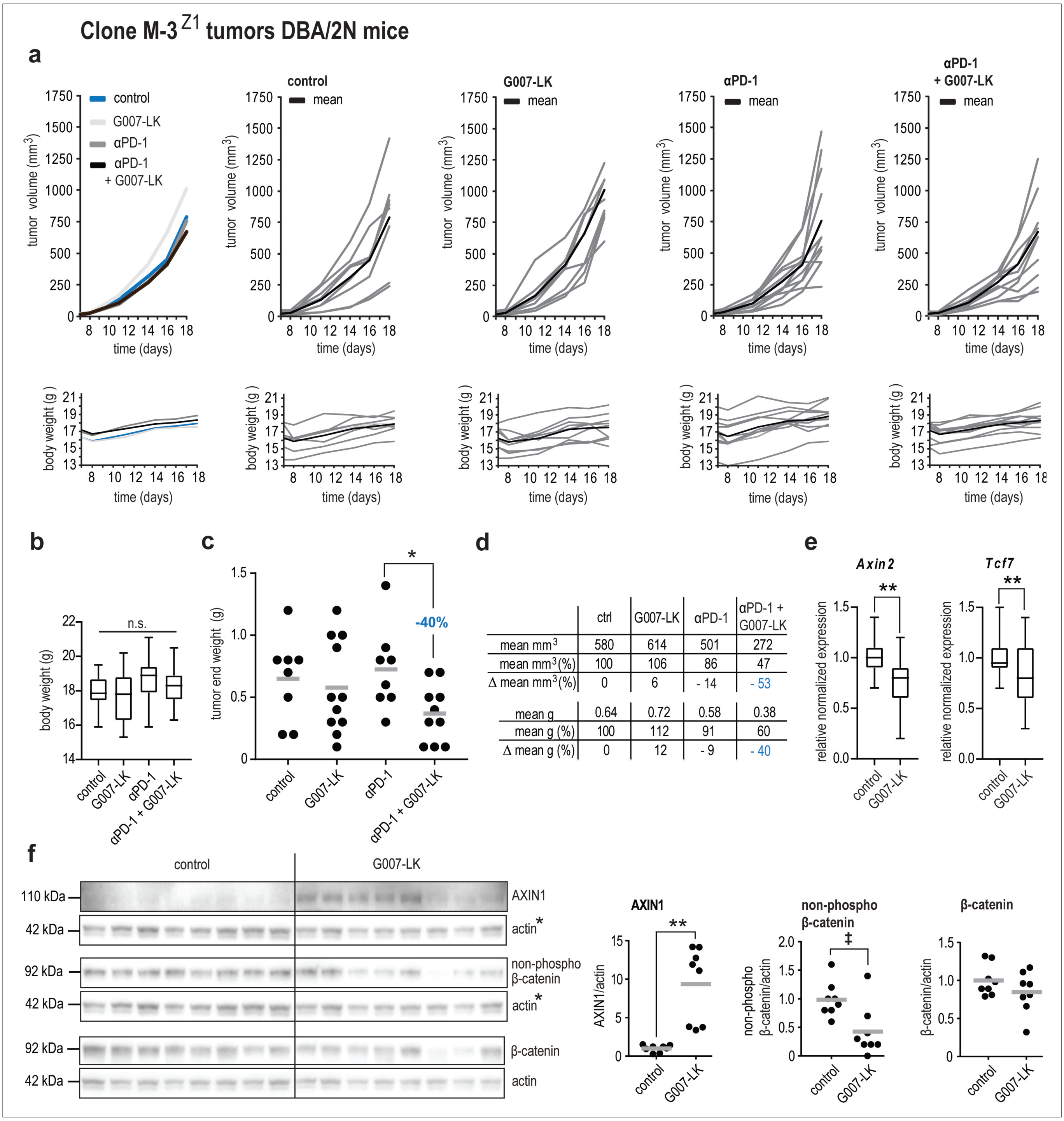
Clone M-3 Z1 tumors DBA/2N mice
a
control
G007-LK
αPD-1
αPD-1 + G007-LK
mean
tumor volume (mm3)
time (days)
body weight (g)
b
n.s.
body weight (g)
c
*
-40%
tumor end weight (g)
d
ctrl | G007-LK | αPD-1 | αPD-1 + G007-LK
mean mm3 | 580 | 614 | 501 | 272
mean mm3(%) | 100 | 106 | 86 | 47
Δ mean mm3(%) | 0 | 6 | - 14 | - 53
mean g | 0.64 | 0.72 | 0.58 | 0.38
mean g (%) | 100 | 112 | 91 | 60
Δ mean g (%) | 0 | 12 | - 9 | - 40
e
Axin2
Tcf7
**
relative normalized expression
f
110 kDa
42 kDa
92 kDa
AXIN1
actin*
non-phospho β-catenin
β-catenin
actin
AXIN1/actin
non-phospho β-catenin/actin
β-catenin/actin
‡

Supplementary Fig. 11 Dual inhibition of tankyrase and PD-1 confers synergistic anti-tumor efficacy in Clone M-3^{Z1} tumors in DBA/2N mice. a, Clone M-3^{Z1} mean (leftmost panels) and single (right panels) tumor (s.c.) volumes (upper panels) and body weights (lower panels) from mice treated from day 8 until day 18 with control diet (*n* = 8, in blue), G007-LK diet (*n* = 9, in light grey), anti-PD-1 (*n* = 11, in dark grey) and combinations of anti-PD-1/G007-LK (*n* = 11, in black). Mean values are indicated by black lines. **b**, No statistically significant (n.s.) differences in mean body end weight. For **b** and **e**: Boxplots show median, first and third quartiles and maximum and minimum whiskers. **c**, Clone M-3^{Z1} tumor end weight reduction upon anti-PD-1/G007-LK treatment (-40%) compared to control (two-tailed t-test = 0.085). Two-tailed t-test is indicated by * ($P < 0.05$). Mean values are indicated by grey lines ± SD.. **d**, Upper table depicts mean tumor end volumes (mm^3), mean relative tumor end volumes (mm^3 [%]) and relative differences when compared to control (Δmm^3 [%]). Lower table depicts mean tumor end weights (g), mean relative tumor end weights (g [%]) and relative differences when compared to control (Δg [%]). **e**, Real-time RT-qPCR analyses of WNT/β-catenin signaling target genes (*Axin2* and *Tcf7*). Combined data from 2 independent measurements with three replicates each is shown. For **e** and **f**: Clone M-3^{Z1} tumors treated from day 8 until day 18 with control diet (*n* = 8) or G007-LK diet (*n* = 8). One-tailed t-tests are indicated by ** ($P < 0.01$) and one-tailed Mann-Whitney rank sum test is indicated by ‡ ($P < 0.01$). **f**, Representative (left panel) and quantified (right panels) immunoblots of tumor extracts showing altered expression of AXIN1, active form of β-catenin (non-phospho, Ser33/37/Thr41) and β-catenin (total). Actin documents equal protein loading and * indicates that the same actin immunoblot is used a loading control for both AXIN1 and non-phospho β-catenin.

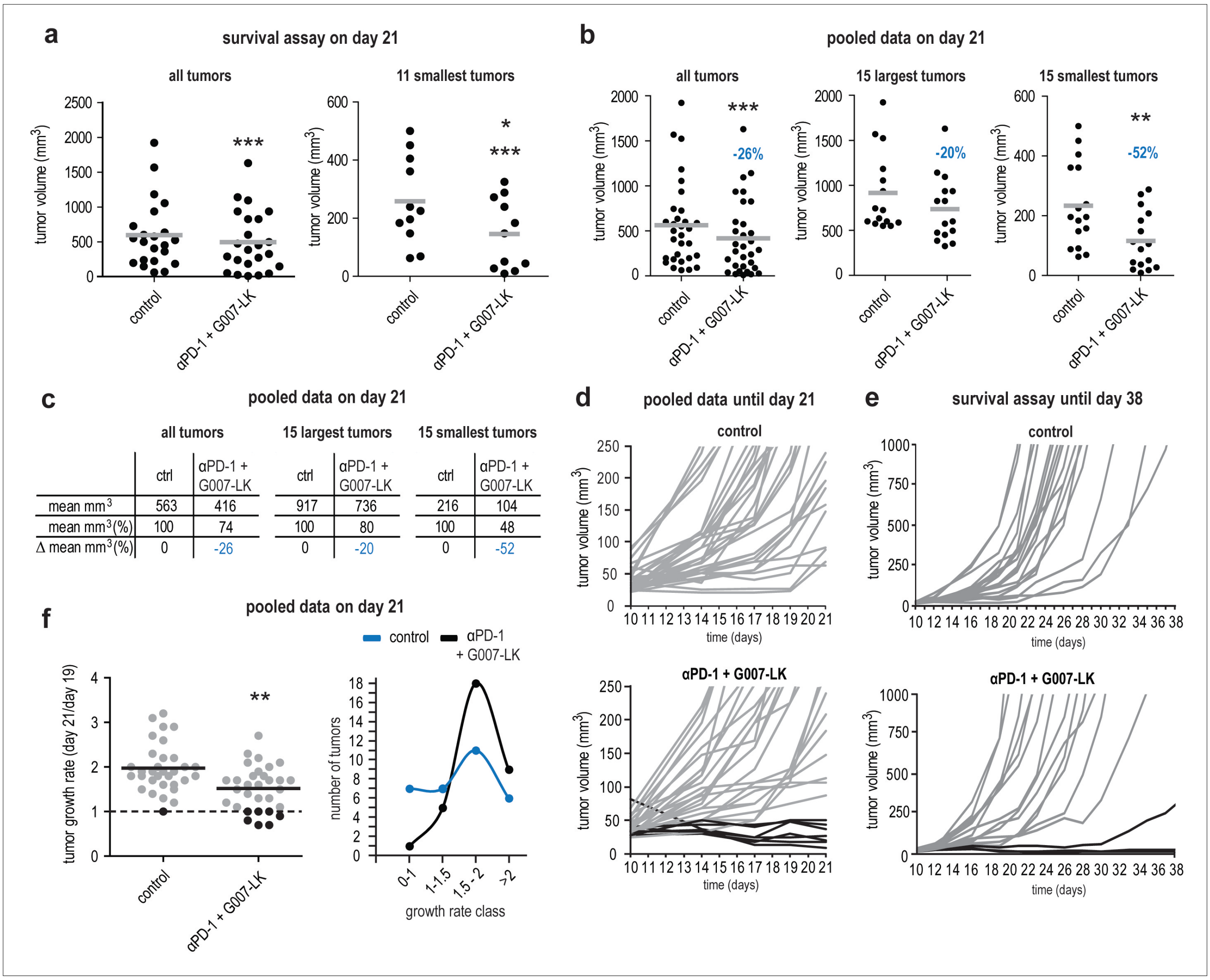

Supplementary Fig. 12 Dual inhibition of tankyrase and PD-1 confers synergistic anti-tumor efficacy in B16-F10 tumors in C57BL/6N mice in a survival assay. **a**, B16-F10 tumor (s.c.) volume reduction for the survival assay on day 21 upon combined anti-PD-1/G007-LK treatment (n = 23) compared to control (n = 22). For all tumors (left panel): Paired t-test is indicated by *** (P < 0.001). For the 11 smallest tumors in each group (right panel): Paired t-test is indicated by *** (P < 0.001) and one-tailed t-test is indicated by * (P < 0.05). Mean values are indicated by grey lines ± SD.. **b**, Plots showing pooled data for tumor volume reduction from the tumor growth and survival assays on day 21 upon combined anti-PD-1/G007-LK treatment (n = 32) compared to control (n = 31). For all tumors (left panel, 26% reduction, paired t-test is indicated by *** [P < 0.001]), the 15 largest tumors in each group (20% reduction, mid panel) and the 15 smallest tumors in each group (52% reduction, one-tailed t-test is indicated by ** [P < 0.01]). **c**, Tables depicting pooled mean tumor end volumes (mm^3), relative mean tumor end volumes (mm^3 [%]) and relative differences when compared to control (Δmm^3 [%]) for all tumors (left panel) along with the 15 largest (mid panel) and smallest (right panel) tumors in each group. **d**, Caliper-based measurements of single s.c. tumor volumes for pooled data from the tumor growth and survival assays until day 21. Black lines depict tumors with volumes scoring below 50 mm^3 (7 out of 31 = 22.5%). **e**, Caliper-based measurements of single s.c. tumor volumes for the survival assay until day 38 with black lines depicting tumors from animals alive on day 38 (3 out of 16 = 19%). **f**, Decrease in tumor growth rate (day 21/day19 = growth rate) for pooled anti-PD-1/G007-LK treatment (growth rate = 1.5, n = 23) compared to control (growth rate = 2.0, n = 22). Plot in left panel: One-tailed t-test is indicated by ** (P < 0.01) and black color indicates tumors with growth rates ≤ 1. Graphs in right panel: Number of tumors in each growth rate class: 0-1, 1-1.5, 1.5-2 and >2. Control in blue and anti-PD-1/G007-LK treatment in black.

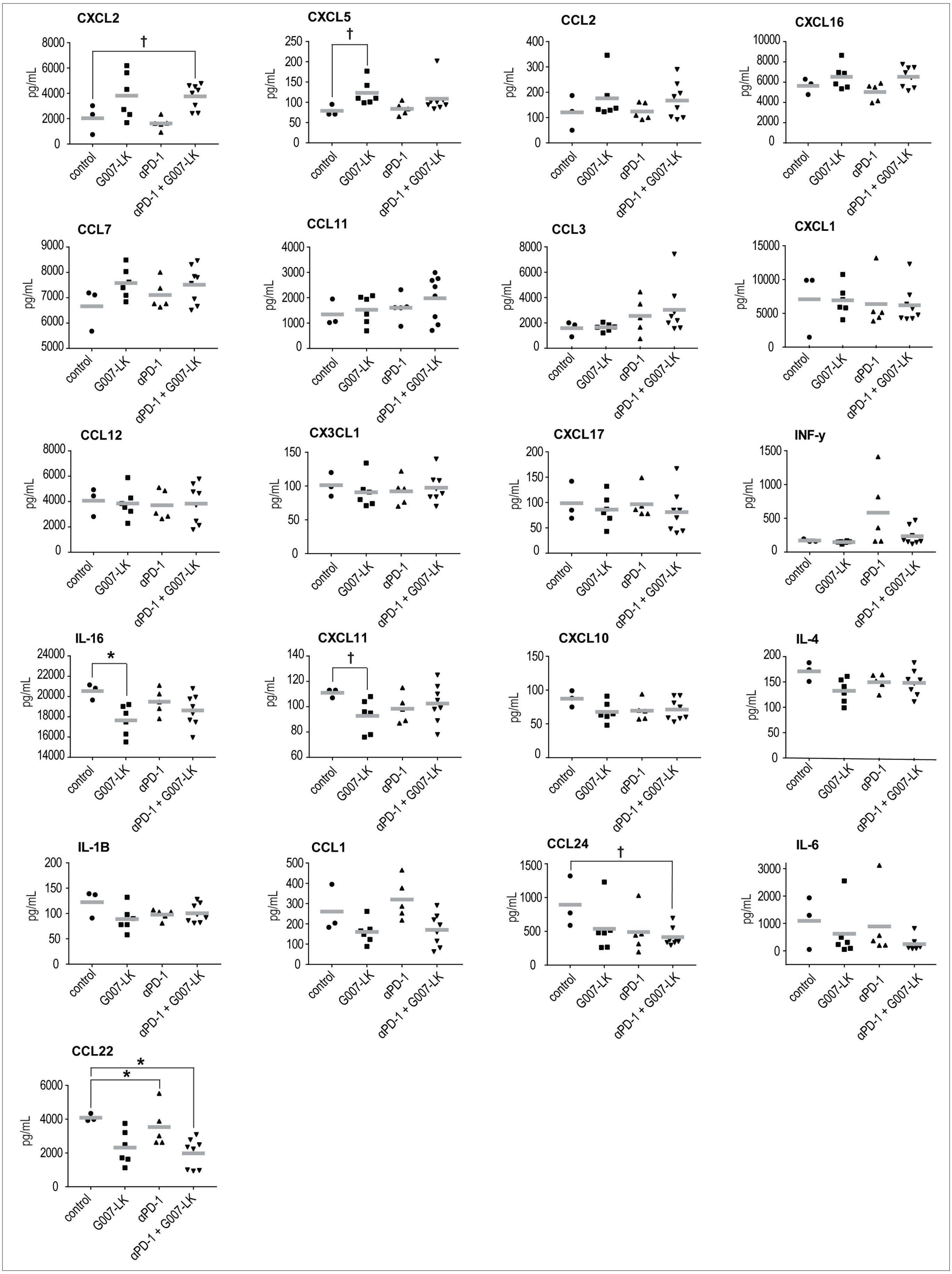
CXCL2
CXCL5
CCL2
CXCL16
CCL7
CCL11
CCL3
CXCL1
CCL12
CX3CL1
CXCL17
INF-y
IL-16
CXCL11
CXCL10
IL-4
IL-1B
CCL1
CCL24
IL-6
CCL22
pg/mL
control
G007-LK
αPD-1
αPD-1 + G007-LK
†
*

Supplementary Fig. 13 Multiplex immunoassay screen of conditioned supernatants from G007-LK-treated B16-F10 tumors reveals alterations in cytokine and chemokine secretion. Conditioned supernatants from matrigel-embedded s.c. B16-F10 tumors screened using multiplex immunoassay upon treatment from day 6 until day 14. Treatments: Control diet (n = 3), G007-LK diet (n = 6), anti-PD-1 (n = 5) and anti-PD-1/G007-LK (n = 8). Mean values are indicated by grey lines. 21 of 33 analyzed cytokines above assay detection threshold. Two-tailed t-tests are indicated by * ($P < 0.05$) and two-tailed Mann-Whitney rank sum tests are indicated by † ($P < 0.05$).

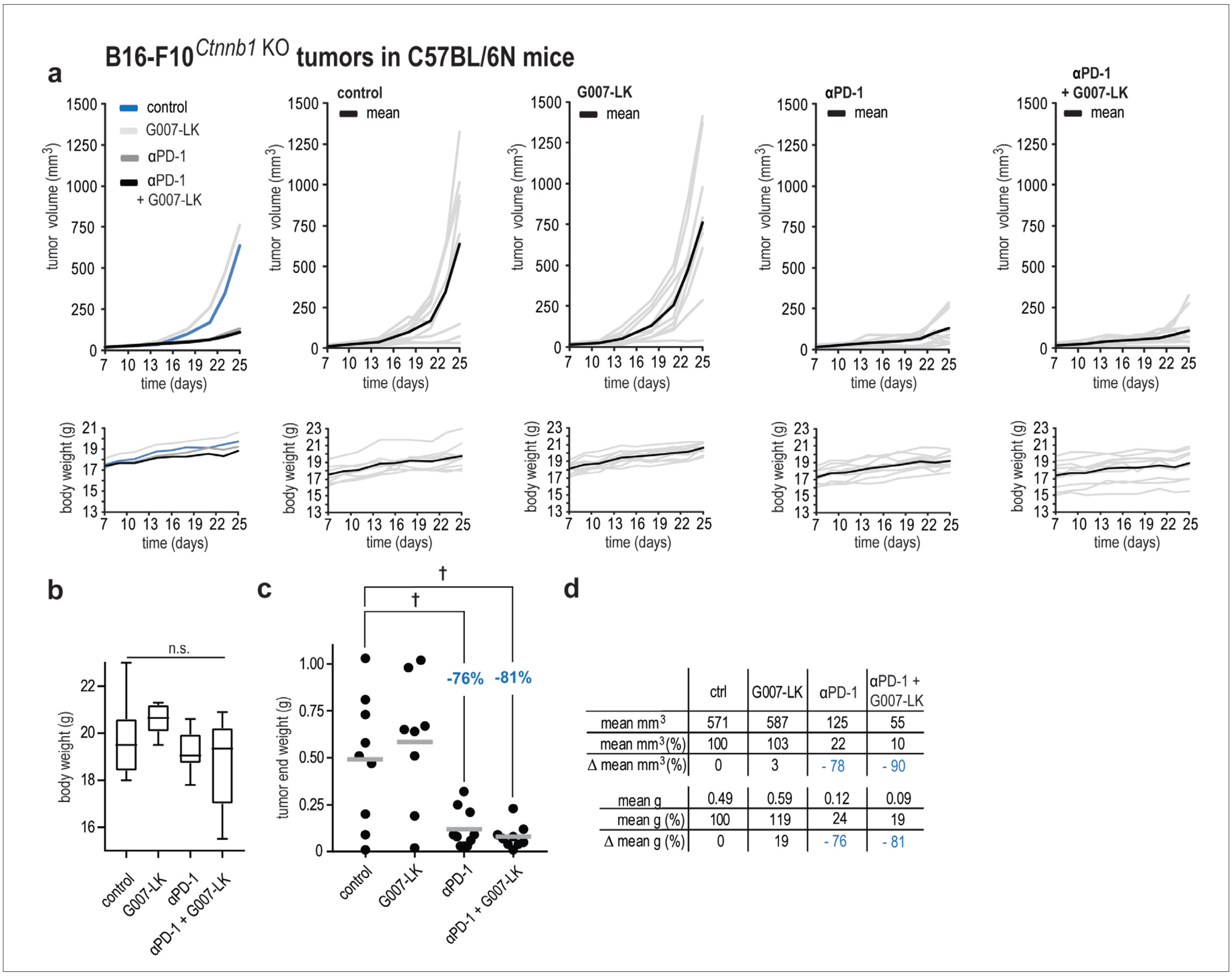

Supplementary Fig. 14 B16-F10$^{Ctnnb1\mathrm{KO}}$ tumors are sensitive to PD-1 inhibition in C57BL/6N mice. a, B16-F10$^{Ctnnb1\mathrm{KO}}$ mean (leftmost panels) and single (right panels) tumor (s.c.) volumes (upper panels) and body weights (lower panels) from mice treated from day 11 until day 25 with control diet (n = 9, in blue), G007-LK diet (n = 9, in light grey), anti-PD-1 (n = 10, in dark grey) and anti-PD-1/G007-LK (n = 10, in black). Mean values are indicated by black lines (right panels). **b**, No statistically significant (n.s.) differences in mean body end weight. Boxplot shows median, first and third quartiles and maximum and minimum whiskers. **c**, B16-F10$^{Ctnnb1\mathrm{KO}}$ tumor (s.c.) end weight reduction upon anti-PD-1 (-76%) and combined anti-PD-1/G007-LK treatment (-81%). Mann-Whitney rank sum tests are indicated by † ($P < 0.05$). Mean values are indicated by grey lines. **d**, Upper table depicts mean tumor end volumes (mm^3), mean relative tumor end volumes (mm^3 [%]) and relative differences when compared to control (Δmm^3 [%]). Lower table depicts mean tumor end weights (g), mean relative tumor end weights (g [%]) and relative differences when compared to control (Δg [%]).

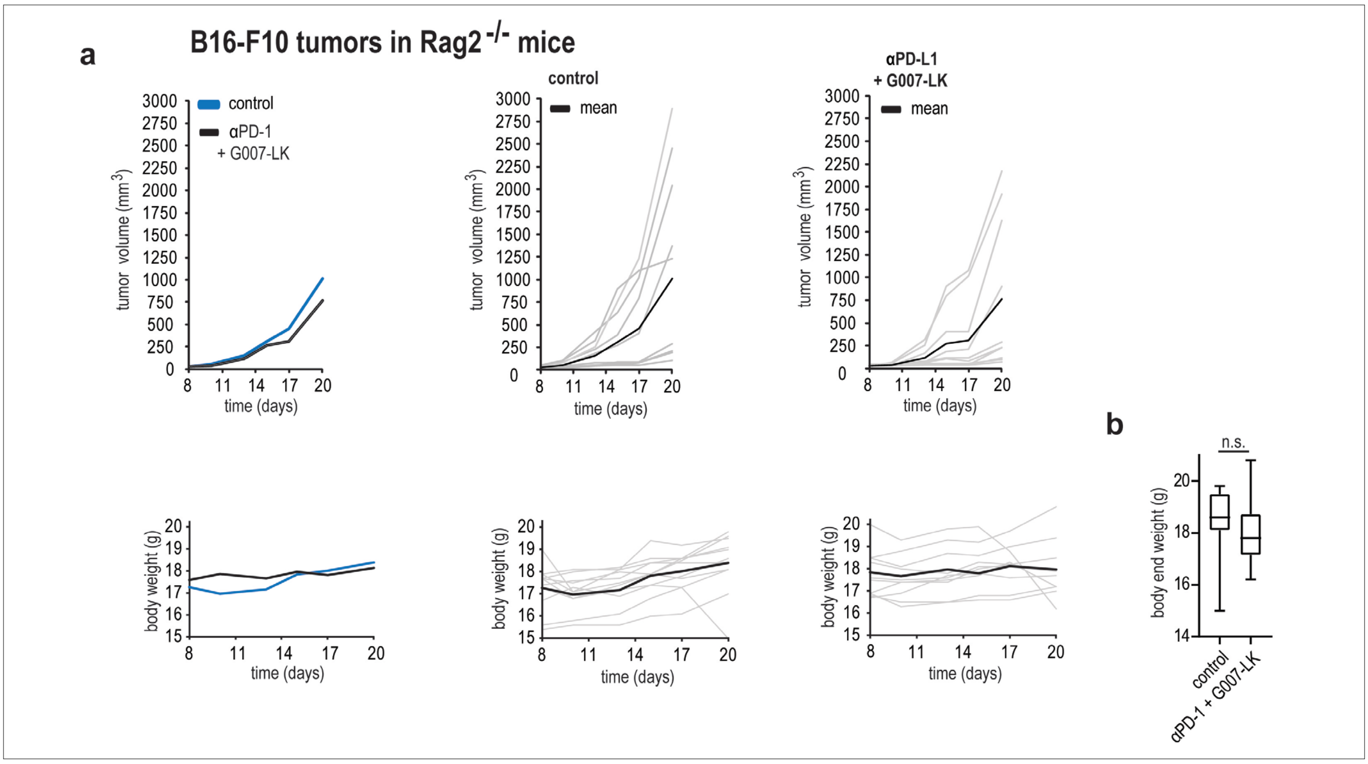

Supplementary Fig. 15 No anti-tumor efficacy of combined tankyrase and checkpoint inhibitor treatment of B16-F10 tumors in Rag2$^{-/-}$ mice. a, Mean B16-F10 (s.c. in Rag2$^{-/-}$ mice)(leftmost panels) and single (right panels) tumor volumes (upper panels) and in addition body weights (lower panels) from mice treated from day 8 until day 20 with control diet (*n* = 11, in blue) or anti-PD-1/G007-LK (*n* = 10, in black). Mean values are indicated by black lines (right panels). **b,** No statistically significant difference in mean body end weight. n.s. = not significant. Boxplot shows median, first and third quartiles and maximum and minimum whiskers.

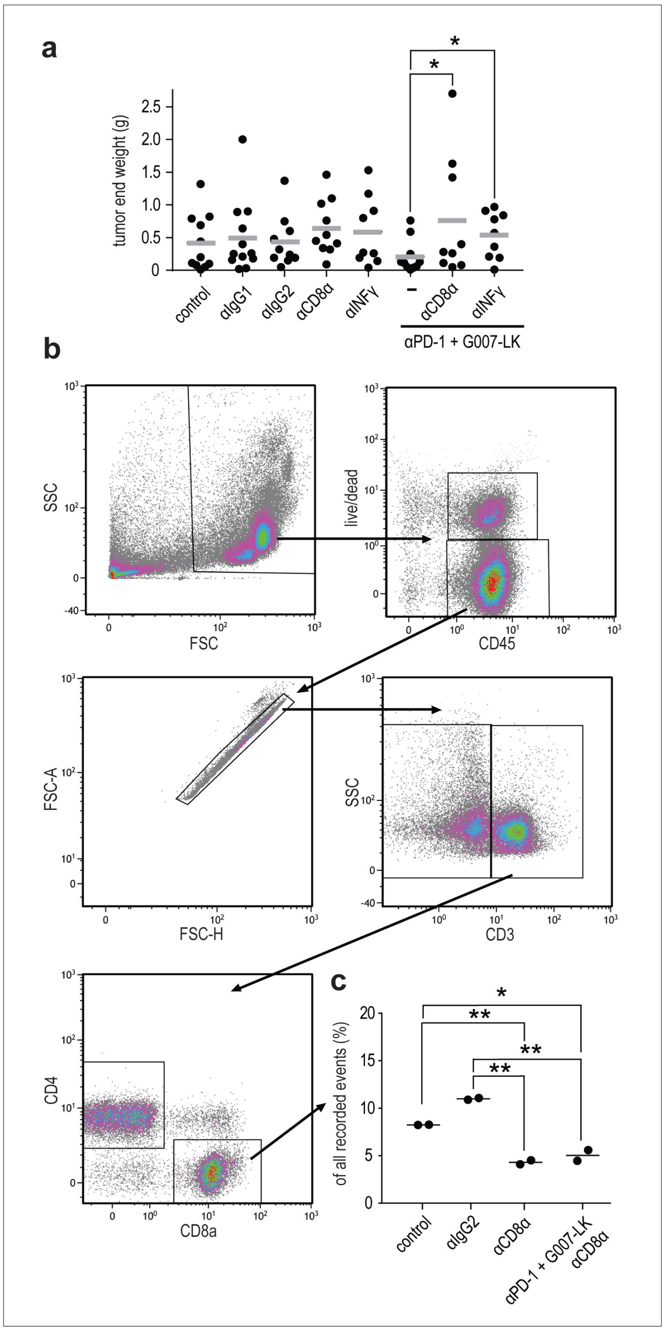

Supplementary Fig. 16 The synergistic anti-tumor effect of combined tankyrase anti-PD-1 inhibition is dependent on IFNγ and CD8$^+$ T cells. a, B16-F10 tumor (s.c.) end weight reduction upon anti-PD-1/G007-LK treatment in C57BL/6N mice from day 10 through day 21. Control diet (*n* = 11), anti-IgG1 isotype control (*n* = 12), anti-IgG2 isotype control (*n* = 10), anti-CD8α (*n* = 10), anti-INFγ (*n* = 9), anti-PD-1/G007-LK (*n* = 10), anti-PD-1/G007-LK /anti-CD8α (*n* = 9) and anti-PD-1/G007-LK /anti-INFγ (*n* = 9). One-tailed t-tests are indicated by * (*P* < 0.05). **b**, Flow cytometry gating strategy for quantification of CD8+ T cells showing representative example using control tumor. **c**, Flow cytometry analysis of CD8$^+$T cells (shown as % of all recorded events) from single cell suspensions derived from spleens (n = 2). Two-tailed t-tests are indicated by * (*P* < 0.05) and ** (*P* < 0.01).

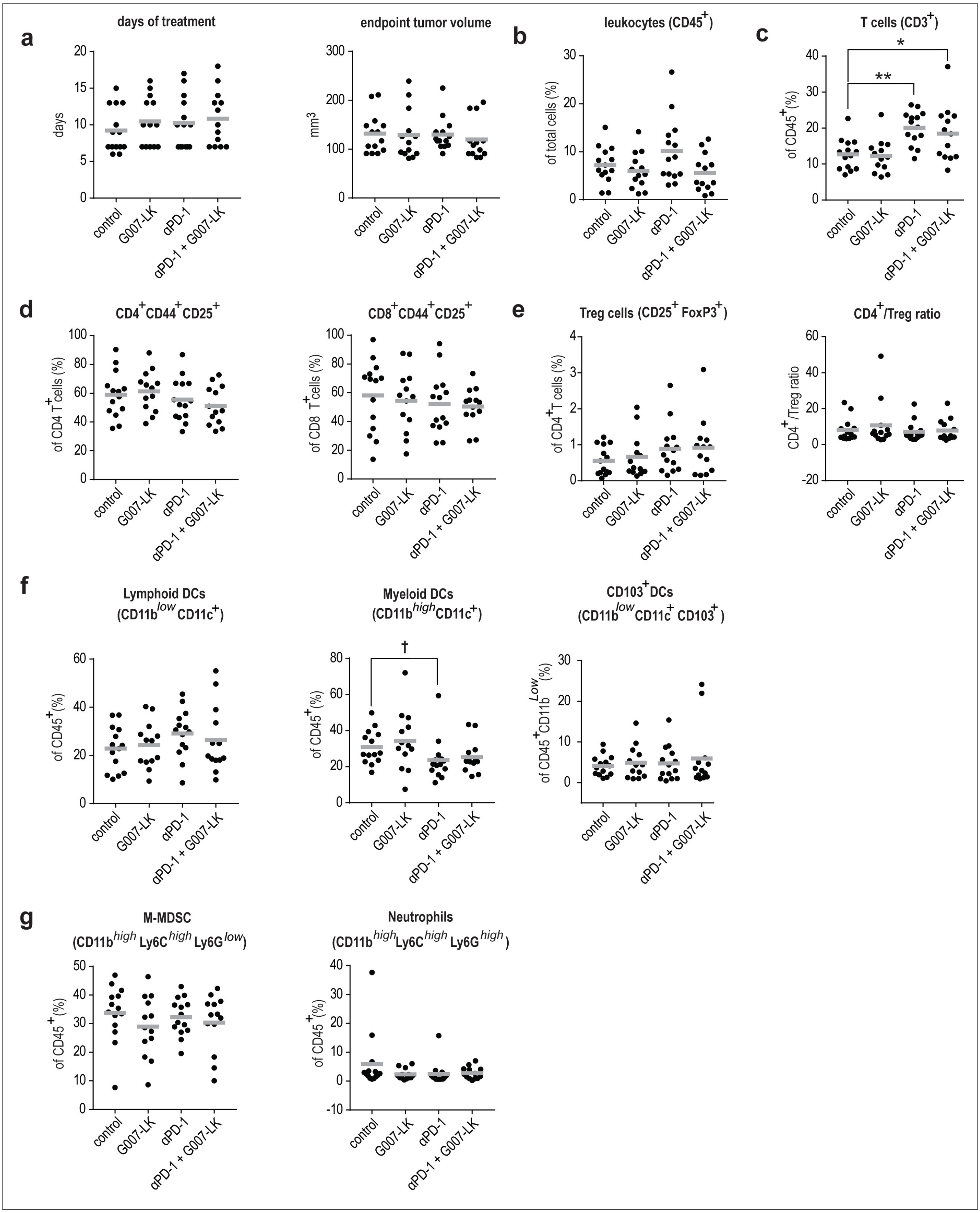
a
days of treatment
days
endpoint tumor volume
mm3
b
leukocytes (CD45+)
of total cells (%)
c
T cells (CD3+)
of CD45+(%)
*
**
d
CD4+CD44+CD25+
of CD4+T cells (%)
CD8+CD44+CD25+
of CD8+T cells (%)
e
Treg cells (CD25+ FoxP3+)
of CD4+T cells (%)
CD4+/Treg ratio
CD4+/Treg ratio
f
Lymphoid DCs
(CD11b low CD11c+)
of CD45+(%)
Myeloid DCs
(CD11b high CD11c+)
†
of CD45+(%)
CD103+DCs
(CD11b low CD11c+ CD103+)
of CD45+CD11b Low (%)
g
M-MDSC
(CD11b high Ly6C high Ly6G low)
of CD45+(%)
Neutrophils
(CD11b high Ly6C high Ly6G high)
of CD45+(%)
control
G007-LK
αPD-1
αPD-1 + G007-LK

Supplementary Fig. 17 Anti-PD-1 treatment alters leukocyte and lymphocyte subsets within B16-F10 tumors. a, Collection time-points (after 7-17 days of treatment, left panel) and tumor volumes (right panel) for s.c. tumors isolated for flow cytometry analysis (shown in **b**-**g**) from B16-F10-challenged C57BL/6N mice treated with control diet (*n* =14), G007-LK diet (*n* = 13), anti-PD-1 (*n* = 14) or anti-PD-1/G007-LK (*n* = 13). For **a**-**g**: Two-tailed t-tests are indicated by * ($P < 0.05$) and ** ($P < 0.01$) while Mann-Whitney rank sum test is indicated by † ($P <$ 0.05). Absence of depicted statistical comparisons indicates lack of statistical significance. Mean values are indicated by grey lines. **b**, Total leukocytes ($CD45^{+}$) shown as % of total cell number. **c**, T cells ($CD3^{+}$) shown as % of leukocytes ($CD45^{+}$). **d**, Quantitation of T-cell subsets:$CD4^{+}$ $CD44^{+}$ $CD25^{+}$ T-cells shown as % of $CD4^{+}$ T-cells, $CD8^{+}$ $CD44^{+}$ $CD25^{+}$ T-cells shown as % of $CD8^{+}$ T-cells. **e**, T_{reg} ($CD25^{+}$ $FoxP3^{+}$) cells plotted as % of $CD4^{+}$ T-cells and ratio of $CD4^{+}$ T-cells to T_{reg} ($CD25^{+}FoxP3^{+}$) cells, (CD4+/ T_{reg} ratios). **f**, Lymphoid DCs ($CD45^{+}$ $CD11b^{low}$ $CD11c^{+}$) and myeloid DCs ($CD45^{+}$ $CD11b^{high}$ $CD11c^{+}$) shown as % of leukocytes ($CD45^{+}$). $CD103^{+}$ DCs ($CD45^{+}$ $CD11b^{\mathit{low}}$ $CD11c^{+}$ $CD103^{+}$) shown as % of $CD11b^{\mathit{low}}$ leukocytes. **g**, Quantitation of sub-populations of myeloid-derived suppressor cells: M-MDSCs ($CD11b^{high}$ $Ly6C^{\mathit{high}}$ $Ly6G^{\mathit{low}}$) and neutrophils ($CD11b^{high}$ $Ly6C^{\mathit{high}}$ $Ly6G^{\mathit{high}}$) shown as % of leukocytes ($CD45^{+}$).

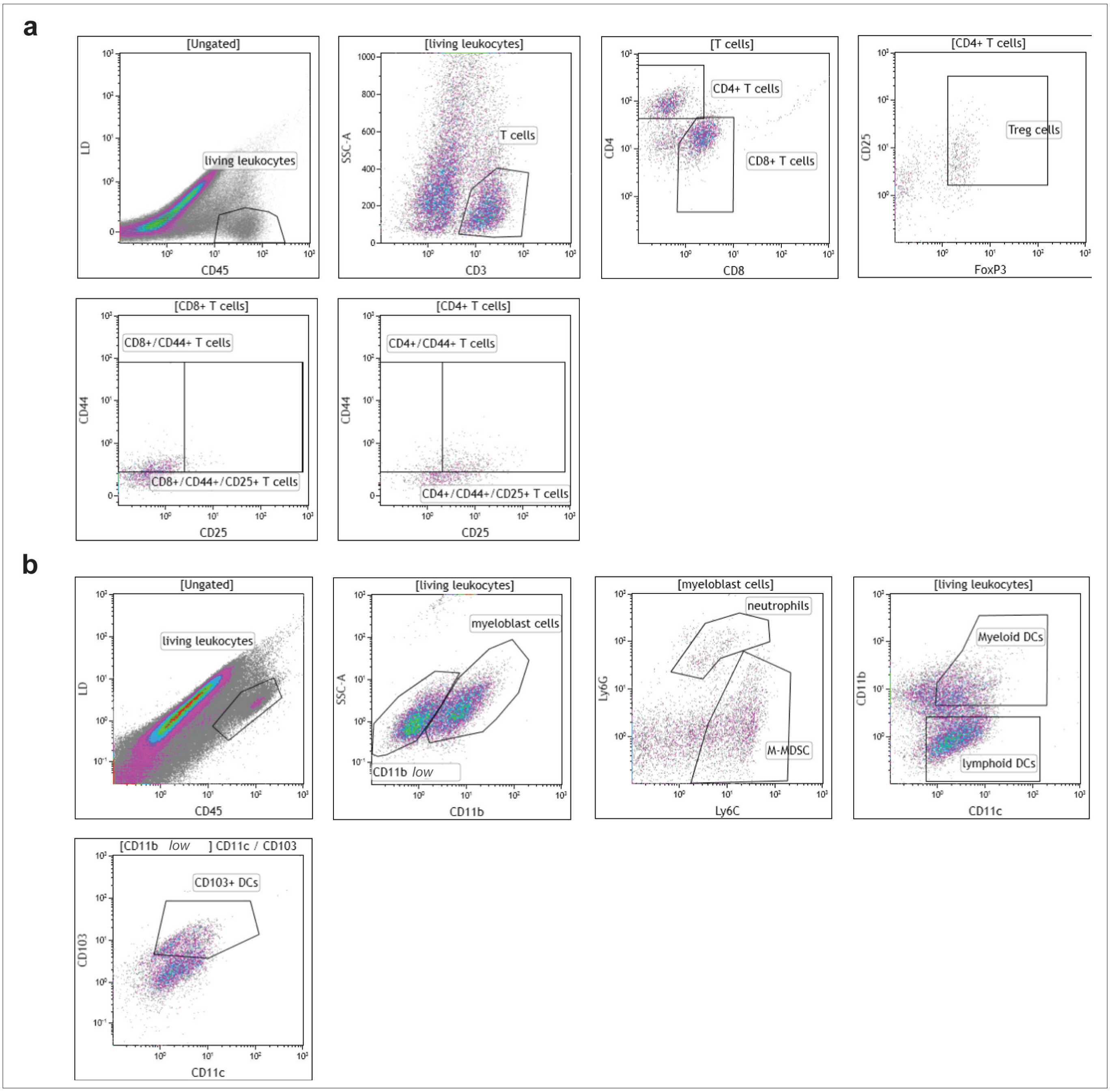

Supplementary Fig. 18 Flow cytometry gating strategy for quantification of intratumoral leukocyte and lymphocyte subsets in treated B16-F10 tumors. Representative examples of flow cytometry gating strategy using primary material from s.c. B16-F10 tumors treated with DMSO, G007-LK, anti-PD-1 or anti-PD-1/G007-LK for determining sub-populations of T cells and myeloid-derived suppressor cells (see Fig. 5 and Supplementary Fig. 17)**.** The gating strategies were defined by ProQinase (Germany). **a**, Gating strategy for T cells: Living leukocytes (CD45^{+}), T cells (CD3^{+}), CD4^{+} T cells (CD3^{+} CD4^{+}), CD8+ T cells (CD3^{+} CD8^{+}), Treg cells (CD3^{+} CD4^{+} CD25^{+} FoxP3^{+}), activated CD4^{+} T cells (CD4^{+} CD44^{+} CD25^{+}) and activated CD8^{+} T cells (CD8^{+} CD44^{+} CD25^{+}). **b**, Gating strategy for myeloid-derived suppressor cells: Living leukocytes (CD45^{+}), M-MDSCs (CD45^{+} CD11b^{high} Ly6C^{high} Ly6G^{low}), neutrophils (CD45^{+} CD11b^{high} Ly6C^{high} Ly6C^{high}), myeloid DCs (CD45^{+} CD11b^{high} CD11c^{+}), lymphoid DCs (CD45^{+} CD11b^{low} CD11c^{+}), CD103^{+} DCs (CD45^{+} CD11b^{low} CD11c^{+} CD103^{+}).

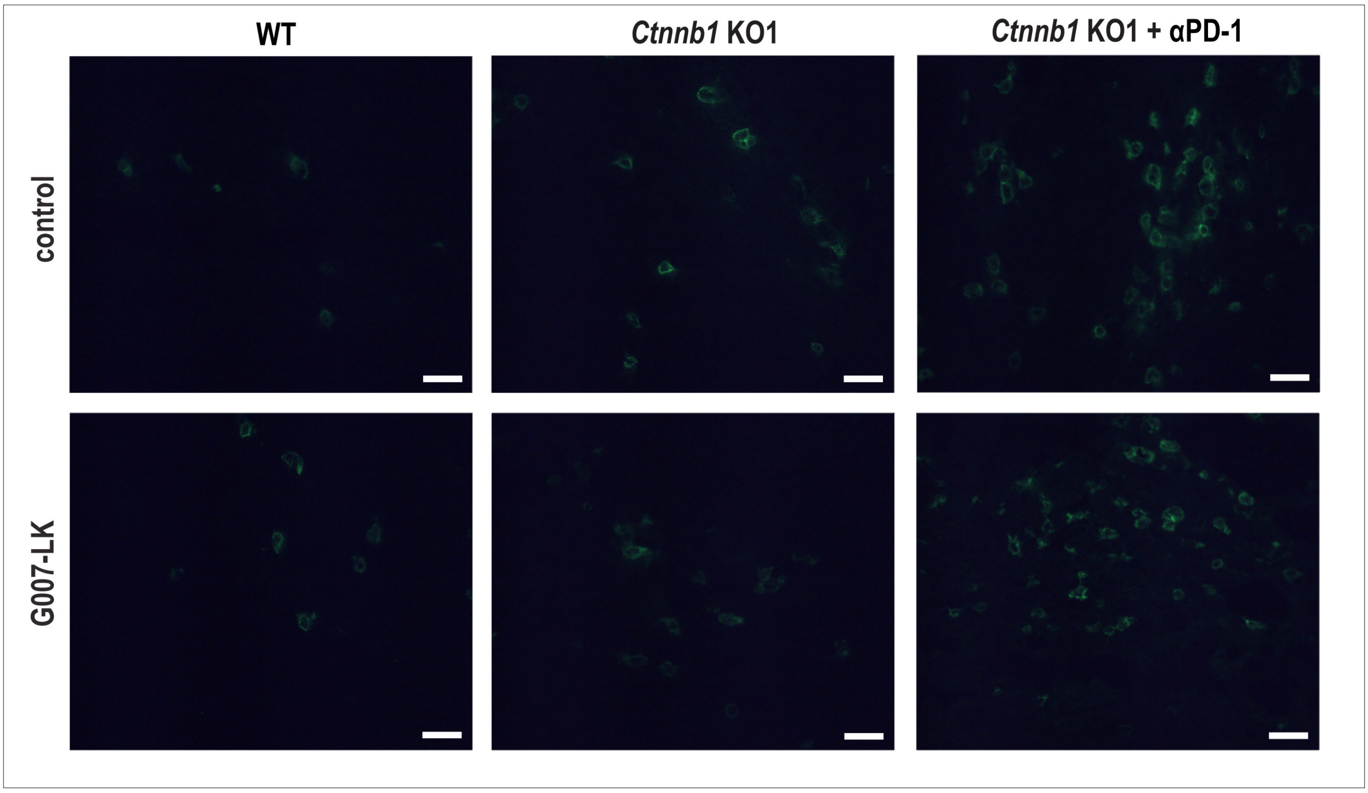

Supplementary Fig. 19 Only PD-1 inhibition, and not loss of β-catenin in the tumors cells, contributes to increased chemotaxis of CD8$^+$ T cells in B16-F10 tumors. Representative images of CD8$^+$ T cells (green) taken from s.c. B16-F10 tumor IHC sections: Wild-type B16-F10 tumors (control diet [*n* = 5], G007-LK diet [*n* = 4]) and B16-F10$^{Ctnnb1\mathrm{KO}}$ tumors (control diet [*n* = 7], G007-LK diet [*n* = 8]) anti-PD-1 [*n* = 10] and anti-PD-1/G007-LK [*n* = 9]). Scale bars: 25 µm (original magnification ×400).

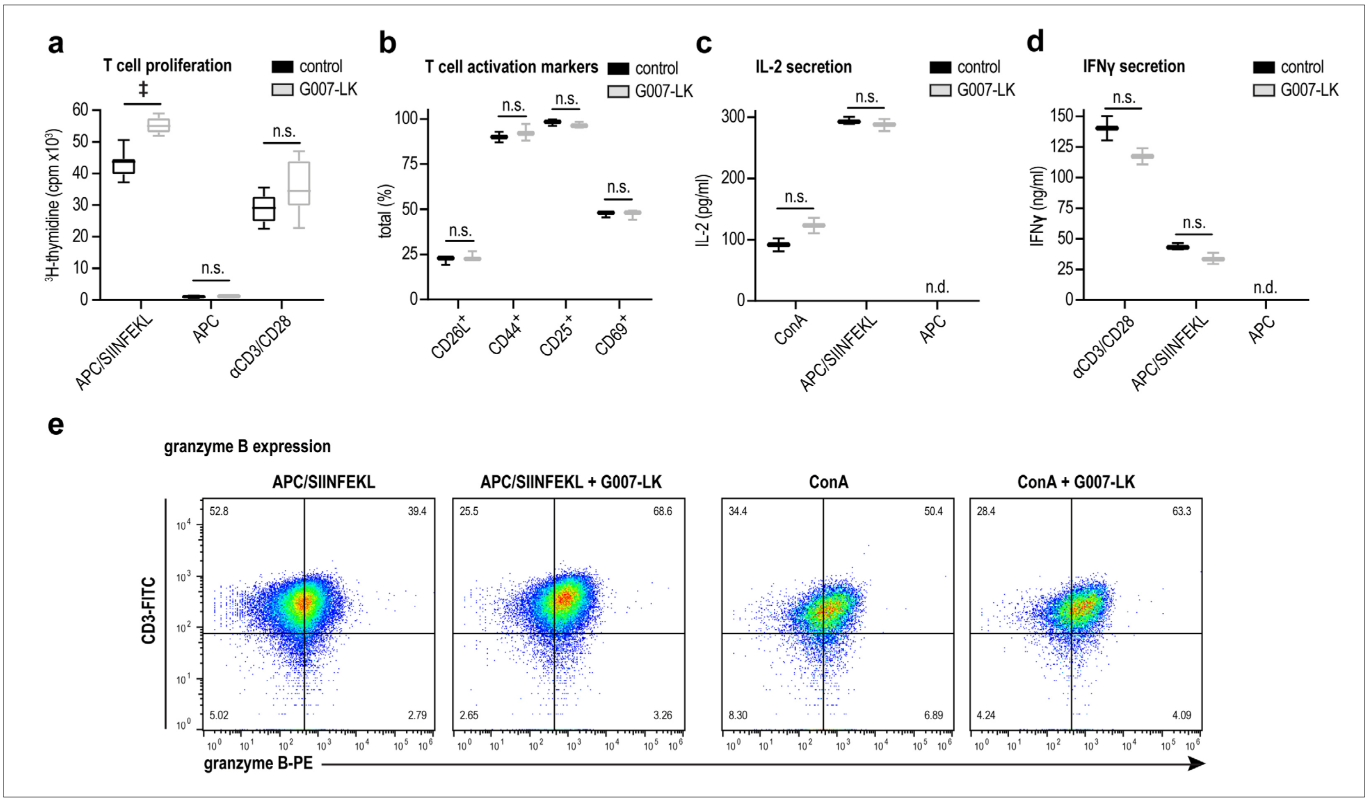

Supplementary Fig. 20 Impact of G007-LK treatment on proliferation and phenotype of ovalbumin-specific CD8$^+$ T cells following antigen-driven or polyclonal activation. a, T cell proliferation upon 72 hours of treatment (96 hours for the anti-CD3/anti-CD28 control). Growth is expressed as counts per minute (cpm) 24 hours after pulsing with ^{3}H-thymidine. Two-tailed Mann-Whitney rank sum test is indicated by ‡ (P < 0.01). For **a**-**d**: Boxplots show median, first and third quartiles and maximum and minimum whiskers. For **a**-**e:** Treatments used: G007-LK (1 µM) or vehicle control (0.01% DMSO). Ovalbumin-specific CD8$^+$ T cells were co-incubated with antigen-presenting cells (APC) in the presence (APC/SIINFEKL) or absence (APC) of the class I (Kb)-restricted peptide epitope SIINFEKL, or with Concanavalin A (ConA) or immobilized anti-CD3/anti-CD28 antibodies for antigen-independent activation. n.s = not significant. n.d = below detection limit. Representative experiments with 8-12 replicates are shown. **b**, Percentage of APC/SIINFEKL-activated CD8$^+$ T cells with positive surface staining for activation markers CD62L, CD44, CD25 and CD69 after 5 days of treatment as determined by flow cytometry. **c**, Secretion of IL-2 in culture supernatant upon 72 hours of treatment following T cell priming, as determined by ELISA. **d**, Secretion of IFNγ in culture supernatant upon 72 hours of treatment following T cell priming, as determined by ELISA. **e**, Representative flow cytometry data showing intracellular expression of granzyme B after 5 days of treatment and ovalbumin-specific CD8$^+$ T-cell activation with APC/SIINFEKL or Concanavalin A. Distribution of cells in percentage of total is given for each quadrant.

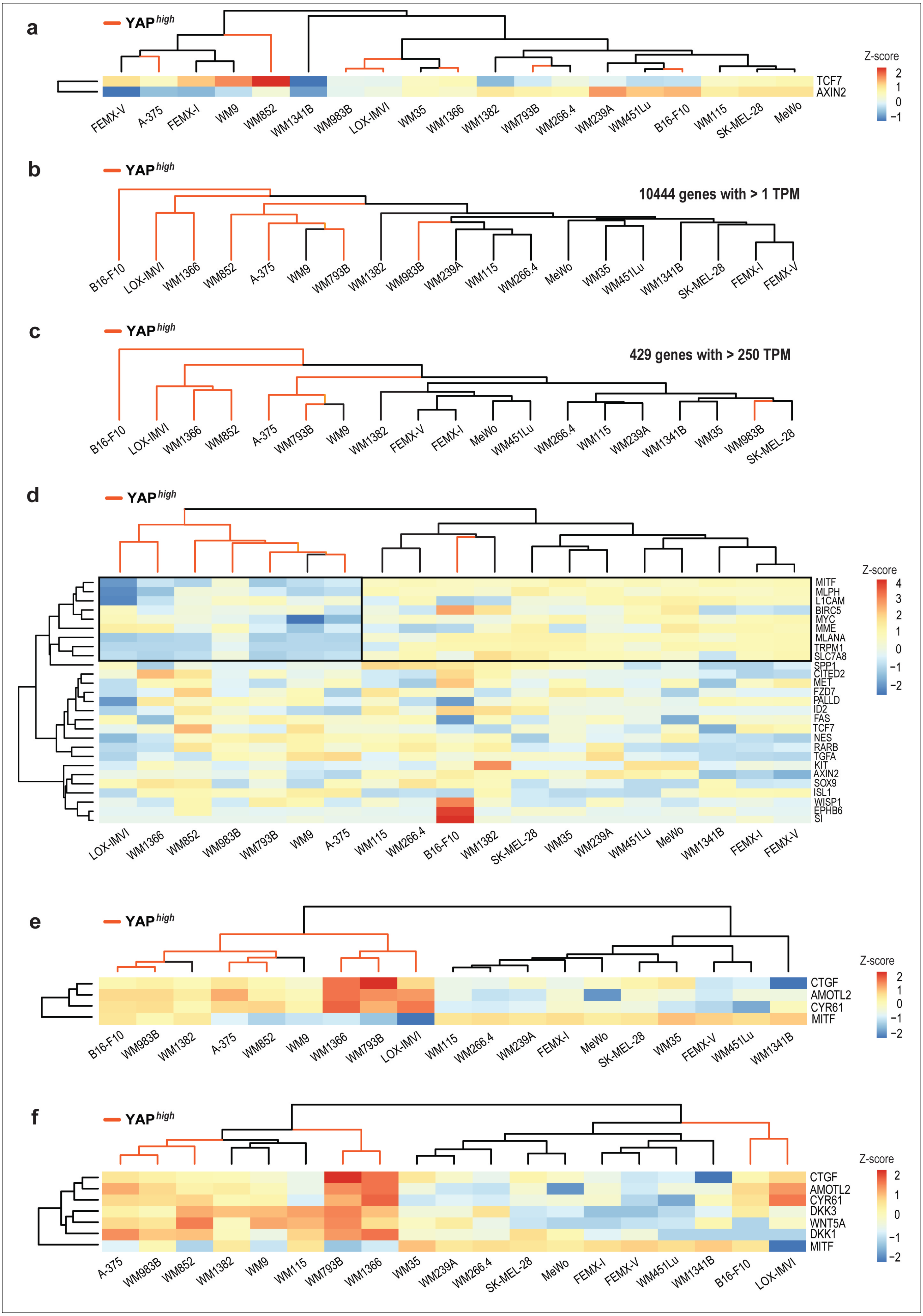
a
YAP high
TCF7
AXIN2
Z-score
FEMX-V A-375 FEMX-I WM9 WM852 WM1341B WM983B LOX-IMVI WM35 WM1366 WM1382 WM793B WM266.4 WM239A WM451Lu B16-F10 WM115 SK-MEL-28 MeWo
b
YAP high
10444 genes with > 1 TPM
B16-F10 LOX-IMVI WM1366 WM852 A-375 WM9 WM793B WM1382 WM983B WM239A WM115 WM266.4 MeWo WM35 WM451Lu WM1341B SK-MEL-28 FEMX-I FEMX-V
c
YAP high
429 genes with > 250 TPM
B16-F10 LOX-IMVI WM1366 WM852 A-375 WM793B WM9 WM1382 FEMX-V FEMX-I MeWo WM451Lu WM266.4 WM115 WM239A WM1341B WM35 WM983B SK-MEL-28
d
YAP high
Z-score
MITF MLPH L1CAM BIRC5 MYC MME MLANA TRPM1 SLC7A8 SPP1 CITED2 MET FZD7 PALLD ID2 FAS TCF7 NES RARB TGFA KIT AXIN2 SOX9 ISL1 WISP1 EPHB6 SI
LOX-IMVI WM1366 WM852 WM983B WM793B WM9 A-375 WM115 WM266.4 B16-F10 WM1382 SK-MEL-28 WM35 WM239A WM451Lu MeWo WM1341B FEMX-I FEMX-V
e
YAP high
Z-score
CTGF AMOTL2 CYR61 MITF
B16-F10 WM983B WM1382 A-375 WM852 WM9 WM1366 WM793B LOX-IMVI WM115 WM266.4 WM239A FEMX-I MeWo SK-MEL-28 WM35 FEMX-V WM451Lu WM1341B
f
YAP high
Z-score
CTGF AMOTL2 CYR61 DKK3 WNT5A DKK1 MITF
A-375 WM983B WM852 WM1382 WM9 WM115 WM793B WM1366 WM35 WM239A WM266.4 SK-MEL-28 MeWo FEMX-I FEMX-V WM451Lu WM1341B B16-F10 LOX-IMVI

Supplementary Fig. 21 High relative YAP signaling activity correlates with low baseline *MITF* expression. **a**, Heatmap and clustering of transcribed WNT/β-catenin signaling target genes (*Axin2* and *Tcf7*) for 18 untreated human melanoma cell lines (pooled triplicates used for RNA sequencing) along with murine B16-F10 melanoma (biological triplicates used for RNA sequencing). For **a**-**f**: Scale bar indicates relative differences in Z-score values for log2 TPMs within each row, all values are from untreated samples. Samples displaying high relative transcription of YAP signaling target genes (YAP^{high}, see Fig. 6a) are highlighted by orange branches in the dendrogram. **b**, Clustering of 10444 transcribed genes with >1 TPMs. **c**, Clustering of 429 transcribed genes with >250 TPMs. Murine B16-F10 does not display cross-species clustering with human samples. **d**, Heatmap and clustering of a panel of 27 markers for β-catenin-controlled melanoma cell fate and proliferation[43]. 6 of 7 samples in the YAP^{high} subset cluster together, - and they cluster particularly together caused by relative lower expression of *MITF* as well as *MLPH*, *MLANA*, *TRPM1* and *SLC7A8* (highlighted by black box). **e**, Heatmap and clustering of transcribed YAP signaling target genes (*Ccn1*, *Ccn2* and *Amotl2*) versus *MITF*. YAP^{high} correlates with relative low *MITF* transcription ($MITF^{low}$). **f**, Heatmap and clustering showing that high relative levels of YAP signaling activity coincides with high expression of DKK3, WNT5a and DKK1 as well as low *MITF* transcription.

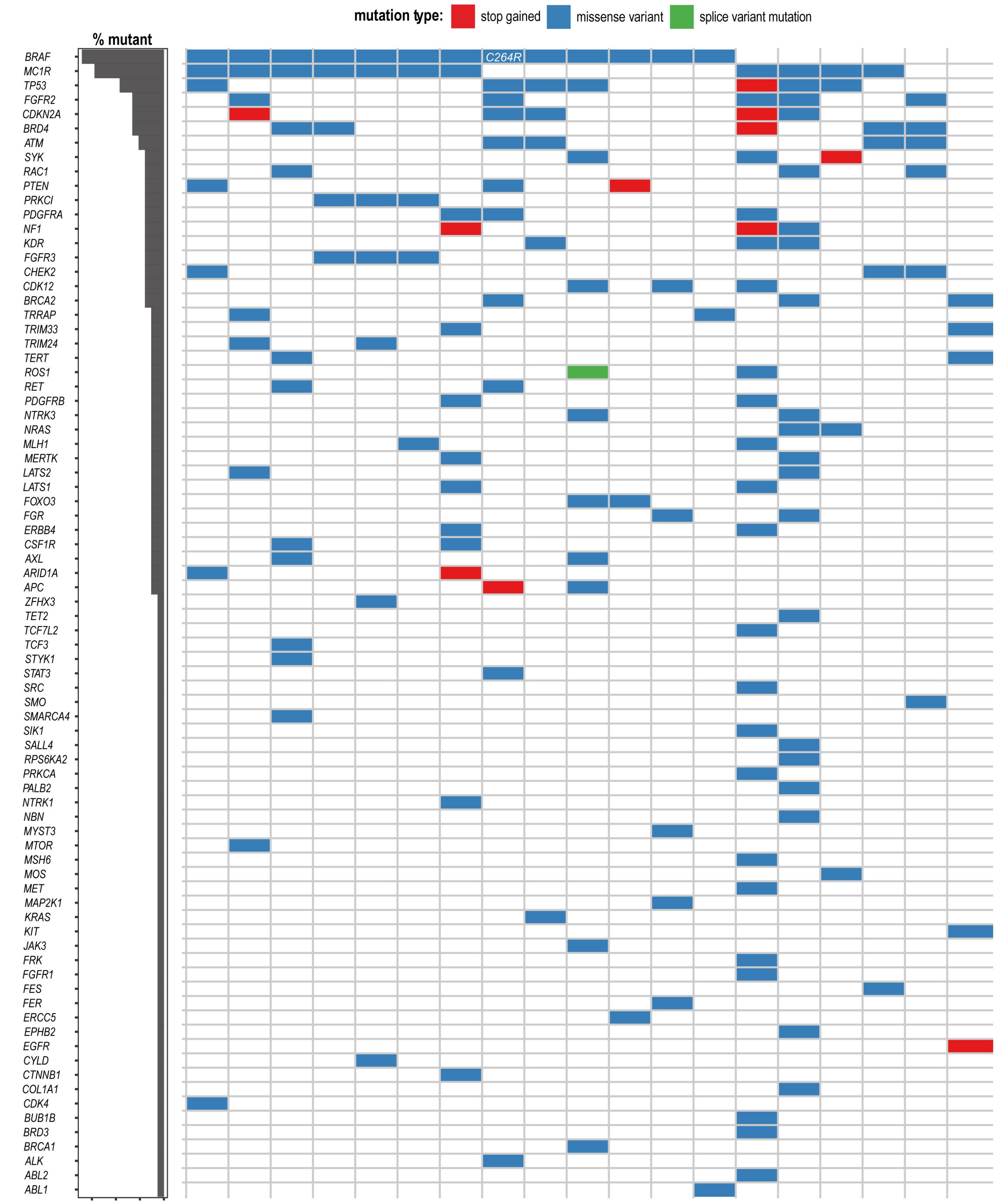

	SK-MEL-28	A-375	WM9	WM239A	WM266.4	WM115	LOX-IMVI	B16-F10	WM983B	WM451Lu	WM793B	WM1341B	WM35	MeWo	WM1366	WM852	FEMX-V	FEMX-I	WM1382
RAS/RAF/MEK/ERK	1	2	6	3	3	3	8	5	3	5	1	5	2	14	10	1	2	3	2
WNT			1				1	1		1				1					
hippo/YAP signaling		1					1							1	1				
PI3K/AKT/mTOR	1	1	1				1	2	1	2	2		1	2	1	1			
p53/DNA repair	2	2			1	1		3	2	2	1		1	4	4	1	2	2	1
nucleosome remodeling	1		2	1			1					1		2	1		1	1	

Supplementary Fig. 22 Oncogenic mutations in human and murine B16-F10 melanoma. Probable cancer driver mutations in 18 human and murine B16-F10 melanoma cell lines identified upon analysis of RNA and DNA gene panel sequencing data, and after whole-exome sequencing of B16-F10 cells[42] listed with the genes with the most frequent mutations at the top[70]. Stop gained mutations are indicated in red, missense mutations in blue, and splice variant mutation in green. The *MC1R* variants are assumed to be germ-line. The bottom table categorizes the mutations into the most frequently affected signaling pathways for each cell line as indicated below. The $BRAF^{C264R}$ mutation in B16-F10 is not homologous to human the $BRAF^{V600E}$ mutation and is indicated in the figure.

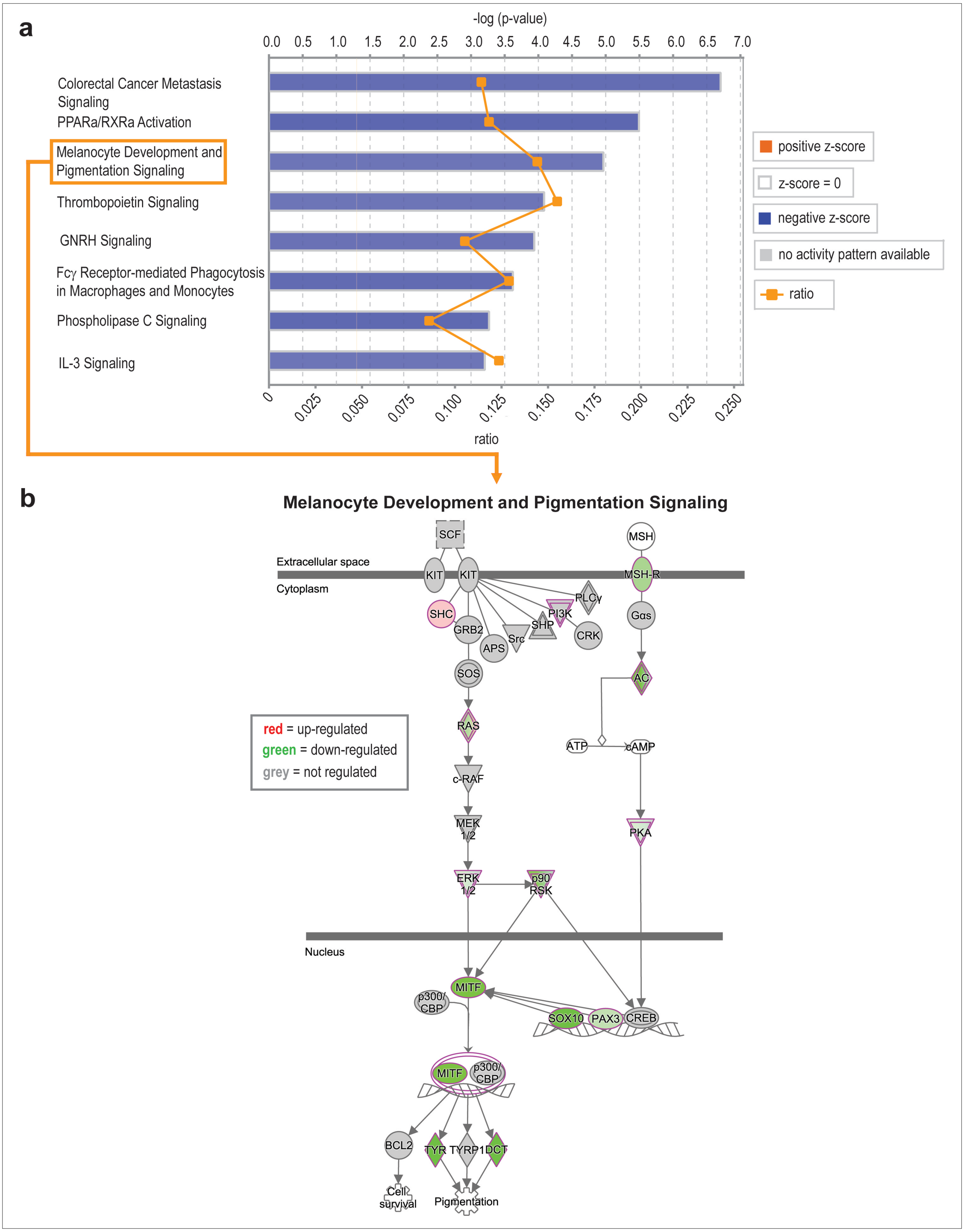
a
-log (p-value)
0.0 0.5 1.0 1.5 2.0 2.5 3.0 3.5 4.0 4.5 5.0 5.5 6.0 6.5 7.0
Colorectal Cancer Metastasis Signaling
PPARa/RXRa Activation
Melanocyte Development and Pigmentation Signaling
Thrombopoietin Signaling
GNRH Signaling
Fcγ Receptor-mediated Phagocytosis in Macrophages and Monocytes
Phospholipase C Signaling
IL-3 Signaling
0 0.025 0.050 0.075 0.100 0.125 0.150 0.175 0.200 0.225 0.250
ratio
positive z-score
z-score = 0
negative z-score
no activity pattern available
ratio
b
Melanocyte Development and Pigmentation Signaling
Extracellular space
Cytoplasm
Nucleus
SCF
MSH
KIT
KIT
MSH-R
PLCγ
PI3K
SHC
SHP
CRK
Gαs
GRB2
Src
APS
SOS
AC
RAS
ATP
cAMP
c-RAF
MEK 1/2
PKA
ERK 1/2
p90 RSK
MITF
p300/ CBP
SOX10
PAX3
CREB
MITF
p300/ CBP
BCL2
TYR
TYRP1
DCT
Cell survival
Pigmentation
red = up-regulated
green = down-regulated
grey = not regulated

Supplementary Fig. 23 IPA core analysis of RNA sequencing data identifies canonical pathways separating the baseline YAP^{high} versus the YAP^{low} groups. a, Differentially expressed genes, when comparing YAP^{high} versus YAP^{low} cell lines (see Fig. 6b), with a corrected *P* value of <0.05 were analyzed for direct relationships in a IPA core analysis. The plot is displaying the eight top canonical pathways identified with core analysis with a -log(*P* value) >3 and an absolute z-score >2. Negative z-score is shown as blue bars along the -log(*P* value) axis. The ratio of genes in this dataset matching the pathway, divided by the total genes in the pathway, is shown as the orange line. **b**, Pathway map for the IPA analysis-identified “Melanocyte Development and Pigmentation Signaling pathway”. Up-regulated RNA expression is indicated in red, down-regulated in green, no regulation in grey. Double lines: small = protein group, large = protein complex. Purple lines = target protein is regulated. Double purple lines = one or more targets of a group/complex is regulated.

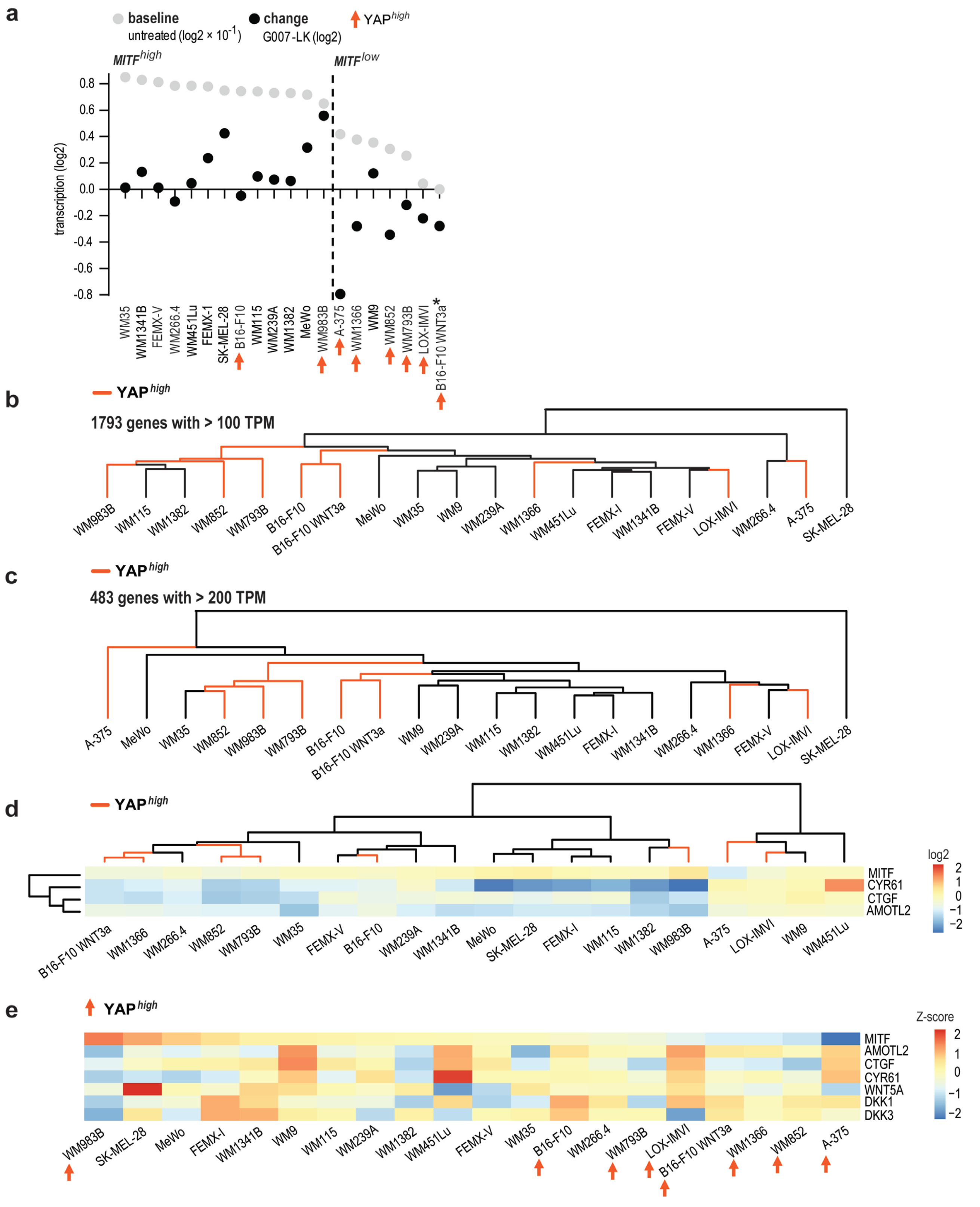
a
baseline
untreated (log2 × 10-1)
change
G007-LK (log2)
YAPhigh
MITFhigh
MITFlow
transcription (log2)
WM35
WM1341B
FEMX-V
WM266.4
WM451Lu
FEMX-1
SK-MEL-28
B16-F10
WM115
WM239A
WM1382
MeWo
WM983B
A-375
WM1366
WM9
WM852
WM793B
LOX-IMVI
B16-F10 WNT3a
b
YAPhigh
1793 genes with > 100 TPM
c
YAPhigh
483 genes with > 200 TPM
d
YAPhigh
log2
MITF
CYR61
CTGF
AMOTL2
e
YAPhigh
Z-score
MITF
AMOTL2
CTGF
CYR61
WNT5A
DKK1
DKK3

Supplementary Fig. 24 High relative baseline YAP signaling activity correlates with low *MITF* expression and G007-LK-induced changes in *MITF* expression cannot be explained alone by changes in YAP or WNT signalling activity. a, *MITF* expression in untreated samples (grey dots sorted descending from left to right, log2-transformed TPMs × 10^{-1}) and upon treatment with G007-LK (1 µM) for 24 hours (black dots, log2 values from treated versus untreated TPMs)(see also Fig. 6b). * indicates that no untreated value is inserted for this sample. The scattered vertical line depicts division between samples with increased (*MITF* ^{high}, left) or decreased (*MITF* ^{low}, right) expression of *MITF* upon tankyrase inhibitor treatment. For **a**-**e**: Change in gene expression for 18 G007-LK-treated (1 µM) human and murine B16-F10 melanoma cell lines is shown. Samples displaying high relative transcription of YAP signaling target genes (YAP^{high}, see Fig. 6a) are highlighted by orange arrows or by orange branches in the dendrogram. B16-F10 WNT3a = WNT3a + G007-LK relative to WNT3a-stimulated control. **b**, Clustering of 1793 genes with changed gene expression with >100 TPMs in >80% of the comparisons (log2). **c**, Clustering of 483 transcribed genes with >200 TPMs (log2). **d**, Heatmap and clustering of transcribed YAP signaling target genes (*Ccn1*, *Ccn2* and *Amotl2*) versus *MITF* upon G007-LK treatment. Decreased YAP signaling (YAP^{decreased}) does not correlate decreased *MITF* transcription (*MITF*^{decreased}). Scale bar indicates relative differences in log2 TPMs. **e**, Heatmap and clustering showing that altered levels of YAP signaling activity (*Ccn1*, *Ccn2* and *Amotl2*) or expression of DKK3, WNT5a and DKK1 do not coincide with decreased *MITF* transcription (sorted descending from right to left).

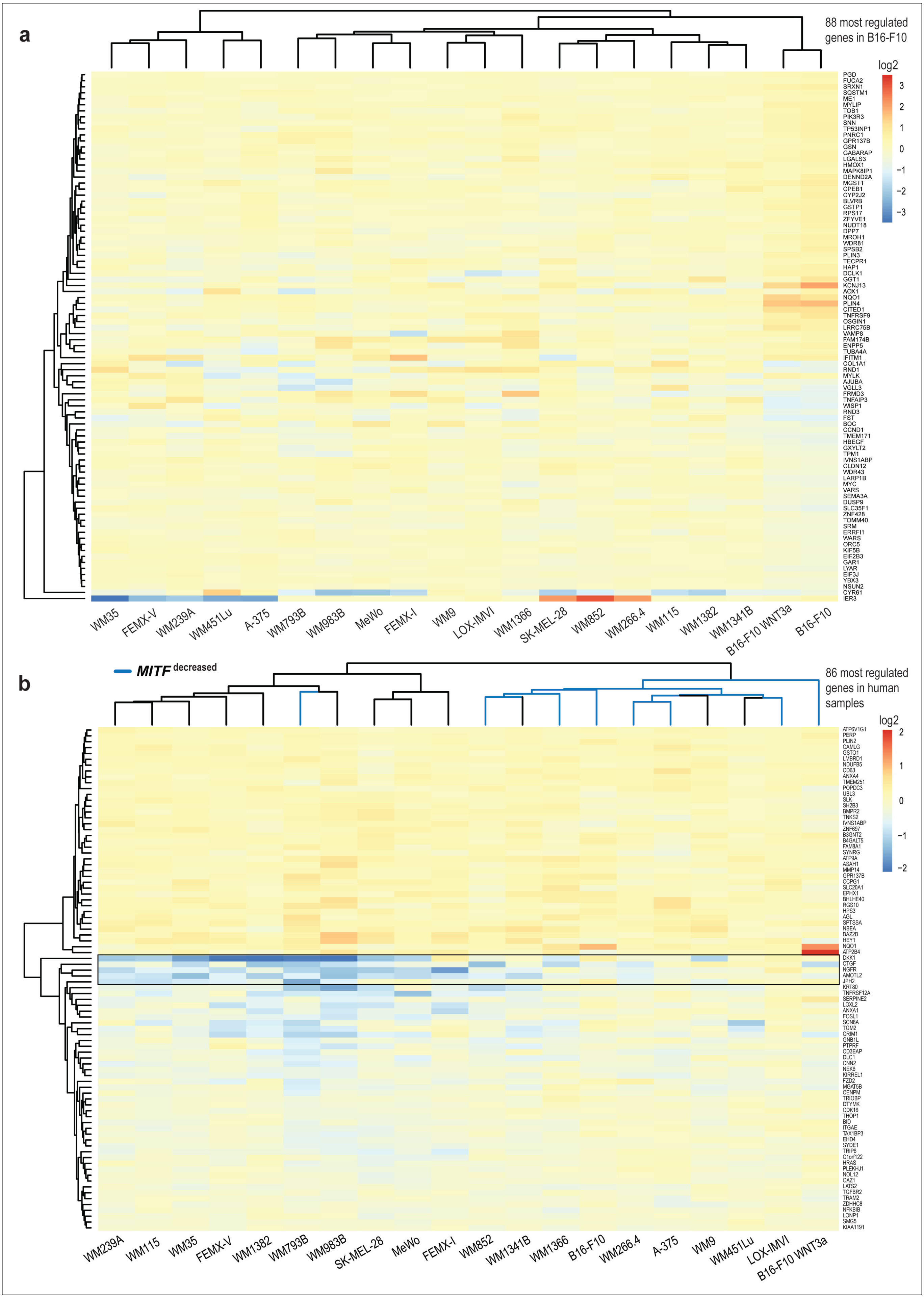
a
88 most regulated genes in B16-F10
log2
3
2
1
0
−1
−2
−3
PGD
FUCA2
SRXN1
SQSTM1
ME1
MYLIP
TOB1
PIK3R3
SNN
TP53INP1
PNRC1
GPR137B
GSN
GABARAP
LGALS3
HMOX1
MAPK8IP1
DENND2A
MGST1
CPEB1
CYP2J2
BLVRB
GSTP1
RPS17
ZFYVE1
NUDT18
DPP7
MROH1
WDR81
SPSB2
PLIN3
TECPR1
HAP1
DCLK1
GGT1
KCNJ13
AOX1
NQO1
PLIN4
CITED1
TNFRSF9
OSGIN1
LRRC75B
VAMP8
FAM174B
ENPP5
TUBA4A
IFITM1
COL1A1
RND1
MYLK
AJUBA
VGLL3
FRMD3
TNFAIP3
WISP1
RND3
FST
BOC
CCND1
TMEM171
HBEGF
GXYLT2
TPM1
IVNS1ABP
CLDN12
WDR43
LARP1B
MYC
VARS
SEMA3A
DUSP9
SLC35F1
ZNF428
TOMM40
SRM
ERRFI1
WARS
ORC5
KIF5B
EIF2B3
GAR1
LYAR
EIF3J
YBX3
NSUN2
CYR61
IER3
WM35
FEMX-V
WM239A
WM451Lu
A-375
WM793B
WM983B
MeWo
FEMX-I
WM9
LOX-IMVI
WM1366
SK-MEL-28
WM852
WM266.4
WM115
WM1382
WM1341B
B16-F10 WNT3a
B16-F10
b
MITF decreased
86 most regulated genes in human samples
log2
2
1
0
−1
−2
ATP6V1G1
PERP
PLIN2
CAMLG
GSTO1
LMBRD1
NDUFB5
CD63
ANXA4
TMEM251
POPDC3
UBL3
SLK
SH2B3
BMPR2
TNKS2
IVNS1ABP
ZNF697
B3GNT2
B4GALT5
FAM8A1
SYNRG
ATP9A
ASAH1
MMP14
GPR137B
CCPG1
SLC20A1
EPHX1
BHLHE40
RGS10
HPS3
AGL
SPTSSA
NBEA
BAZ2B
HEY1
NQO1
ATP2B4
DKK1
CTGF
NGFR
AMOTL2
JPH2
KRT80
TNFRSF12A
SERPINE2
LOXL2
ANXA1
FOSL1
SCN8A
TGM2
CRIM1
GNB1L
PTPRF
CD3EAP
DLC1
CNN2
NEK6
KIRREL1
FZD2
MGAT5B
CENPM
TRIOBP
DTYMK
CDK16
THOP1
BID
ITGAE
TAX1BP3
EHD4
SYDE1
TRIP6
C1orf122
HRAS
PLEKHJ1
NOL12
OAZ1
LATS2
TGFBR2
TRAM2
ZDHHC8
NFKBIB
LONP1
SMG5
KIAA1191
WM239A
WM115
WM35
FEMX-V
WM1382
WM793B
WM983B
SK-MEL-28
MeWo
FEMX-I
WM852
WM1341B
WM1366
B16-F10
WM266.4
A-375
WM9
WM451Lu
LOX-IMVI
B16-F10 WNT3a

Supplementary Fig. 25 Top lists for G007-LK-induced regulation of gene expression. a, The 88 most regulated genes in B16-F10 samples. Murine B16-F10 samples do not cluster with human samples. For **a** and **b**: Heatmap and clustering of change in gene expression for 18 G007-LK-treated (1 µM) human and murine B16-F10 melanoma cell lines. Scale bar indicates log2-values. B16-F10 WNT3a = WNT3a + G007-LK relative to WNT3a-stimulated control. **b**, The 86 most regulated genes in human samples. The clustering is in particular orchestrated by changes in expression of *DKK1*, *CCN2*, *NGFR*, *AMOTL2* and *JPH2* (highlighted by black box). 7 of 8 samples in the *MITF*^{decreased} subset (see Fig. 6b), including the B16-F10 samples, are found on the right side of the heatmap (highlighted by blue branches in the dendrogram).

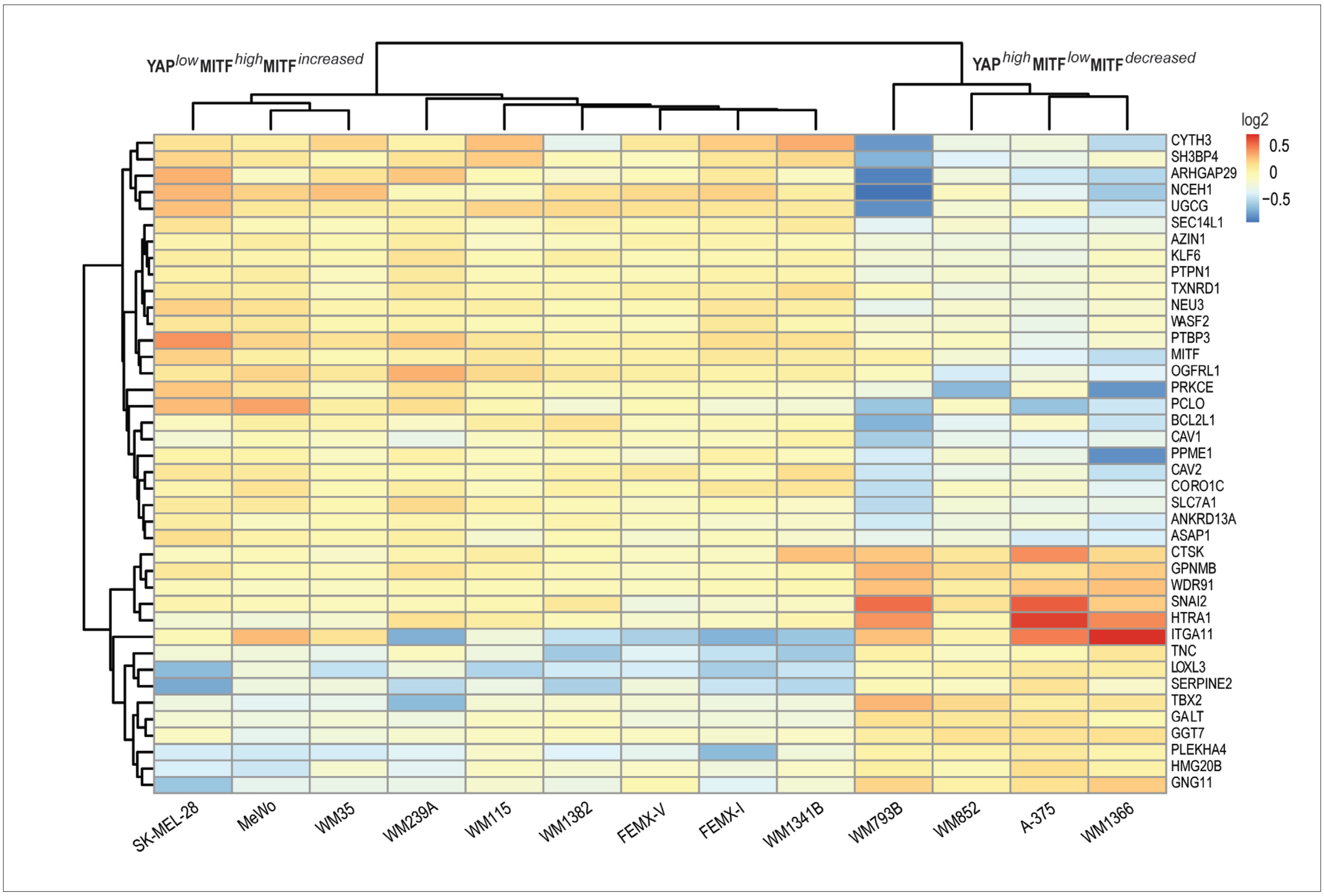

Supplementary Fig. 26 Tankyrase inhibition induces a transcriptional program subdividing the YAP^{low}*MITF*^{high}*MITF*^{increased} and YAP^{high}*MITF*^{low}*MITF*^{decreased} groups. Change in gene expression for G007-LK-treated (1 µM) YAP^{low}*MITF*^{high}*MITF*^{increased} (left) versus YAP^{high}*MITF*^{low}*MITF*^{decreased} (right) subgroups of human cell lines (see Fig. 6e). Differentially expressed genes with adjusted *P* value <0.01 are shown. Scale bar indicates relative differences in log2 counts.

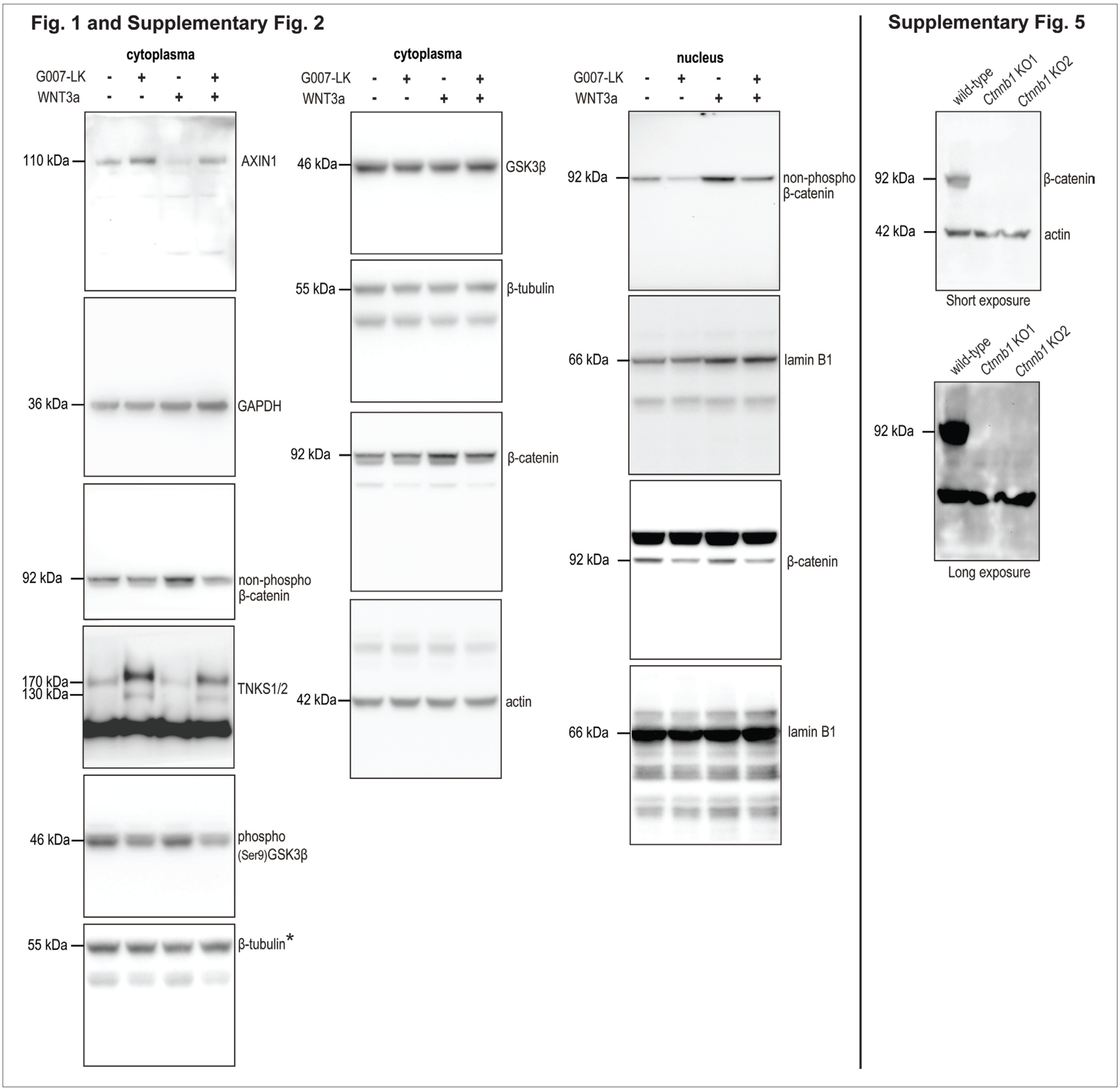

Supplementary Fig. 27 Full length immunoblots for Fig. 1 and Supplementary Fig. 2 and 5. * indicates that the same β-tubulin immunoblot is used as loading control for both TNKS1/2, (phospho[ser9]GSK3β) and β-catenin (non-phospho, Ser33/37/Thr41).

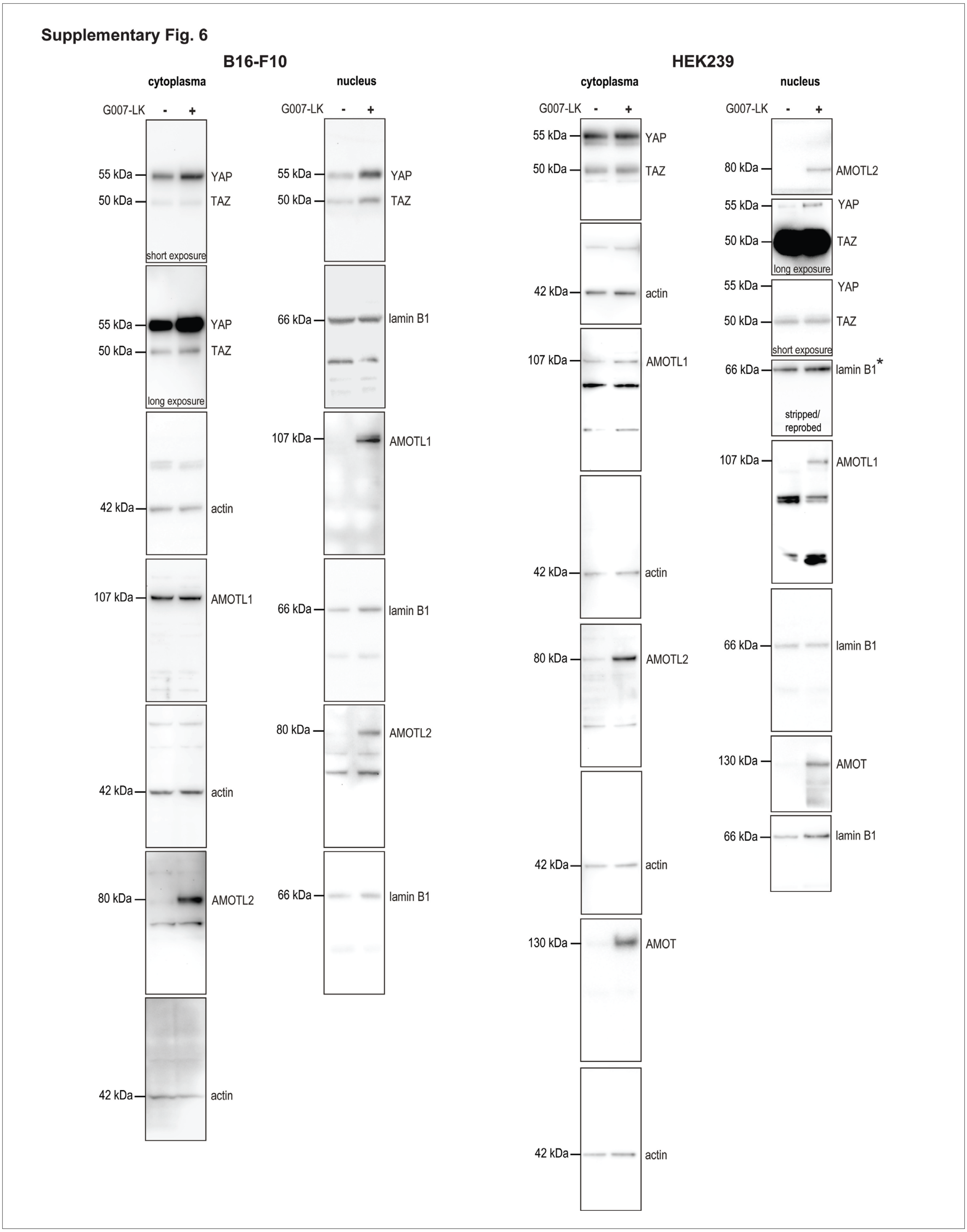

Supplementary Fig. 28 Full length immunoblots for Supplementary Fig. 6. * indicates that the same lamin B1 immunoblot is used as loading control for YAP, TAZ and AMOTL2.

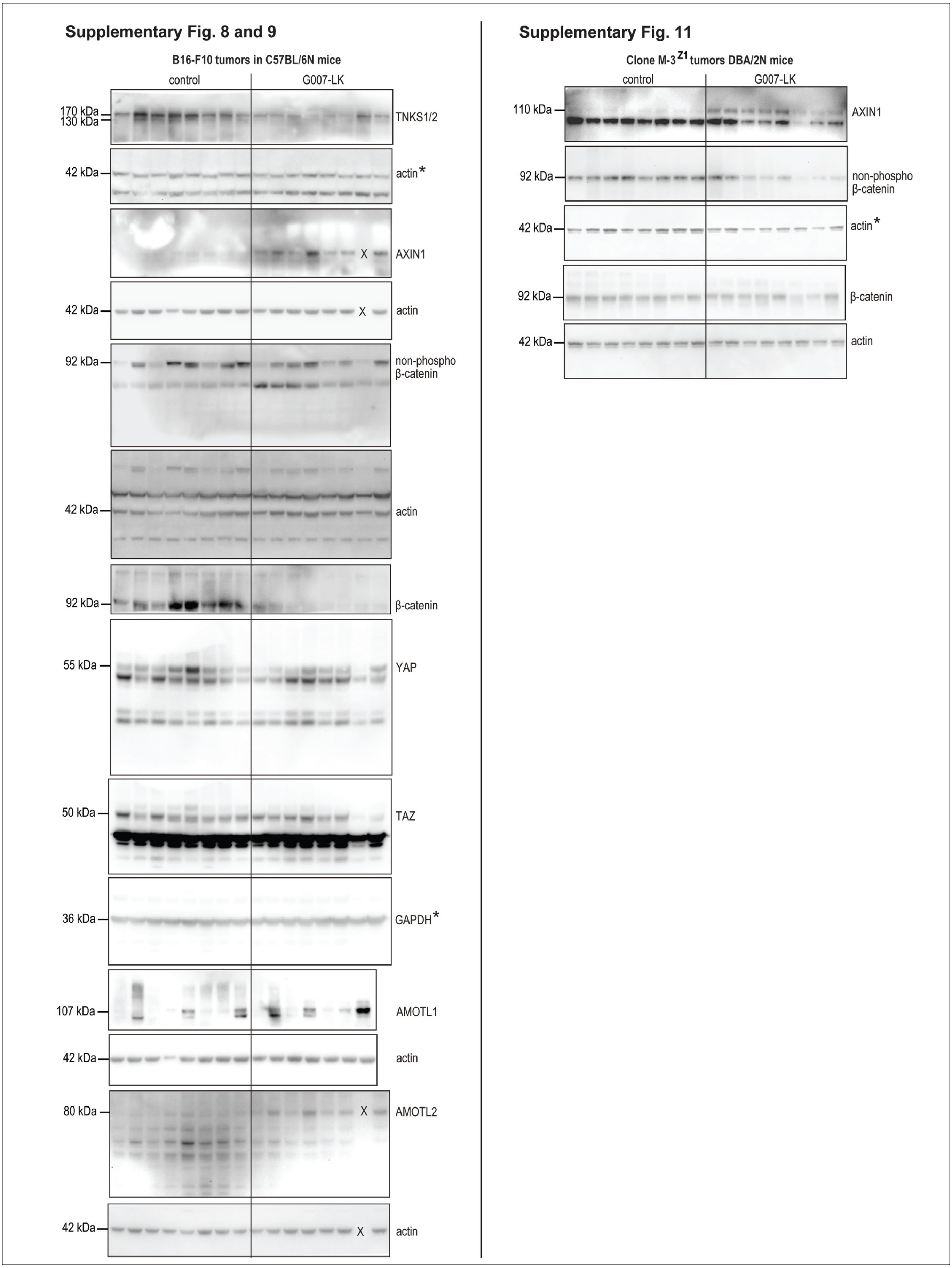

Supplementary Fig. 29 Full length immunoblots for Supplementary Fig. 8, 9 and 11. * indicates that the same actin immunoblot is used as loading control for TNKS1/2 and β-catenin, GAPDH immunoblot is used as loading control for YAP and TAZ (Supplementary Fig. 8 and 9) and actin immunoblot is used as loading control for AXIN1 and non-phospho β-catenin.

a

G007-LK / control

Upstream Regulator	Expr Log Ratio	Molecule Type	Activation z-score	p-value of overlap	Target molecules in dataset
MYC	-0.43	TR	-3.4	8.7E-13	BDNF,C1QBP,CCND1,CD9,CITED1,COL1A1,COL8A1,CXCL10,DDX21,DKC1,DUSP5,EIF2B3,FBL,FOXF1,FTH1,GABARAP,Gar1,GART,GCSH,GGT1,HMGA1,HMOX1,HSPA9,HSPD1,IER3,ITM2B,KLF4,LAMP2,Mt1,MYBBP1A,MYC,NOP56,NOP58,NQO1,ODC1,PA2G4,PEG10,PPAT,PRMT1,PYCR1,RRS1,RUVBL1,RUVBL2,SHMT2,SIRT2,SRM,TIMP1,TOB1,Tpm1,UGT1A6,VARS,WISP1,YBX3
NFE2L2	0.00	TR	3.2	1.2E-14	ALDH3A1,AOX1,BDNF,CAT,COL1A1,CREG1,CCN2,CXCL10,FTH1,FTL,Gsta4,GSTA5,GSTM1,GSTM5,GSTP1,HMOX1,HSPA9,IFRD1,IMPDH1,MAP1LC3B,ME1,MGST1,Mt1,NQO1,OSGIN1,PGD,PLIN2,RRS1,RUVBL1,SHMT2,SQSTM1,SRXN1,Tpm1,UGT1A6
PPARG		LDNR	3.1	2.0E-04	ATP6V1D,CAT,CCND1,COL1A1,CCN2,CTPS1,EHHADH,FST,HMOX1,INSIG1,KLF4,ME1,MGST1,ODC1,PLIN2,PLIN4,PRKG2,VAMP5,VAMP8
NR1H3		LDNR	2.6	3.5E-05	ACSL3,CAT,CXCL10,Gsta4,GSTA5,GSTM5,GSTP1,Mt1,MYLIP,TNFRSF9
NR1I2		LDNR	2.4	2.6E-04	CAT,Gsta4,GSTA5,GSTM1,GSTM5,GSTP1,INSIG1,MGST1,PGD,UGT1A6
NOTCH1	-0.09	TR	-2.4	7.6E-04	ANKRD1,CCND1,COL1A1,CCN2,CCN1,EFNB2,ESM1,HMOX1,MYC,MYLK,PTGDS,RND3
HNF4A		TR	2.4	2.3E-03	ADSS,AJUBA,ALDH3A1,ARHGEF19,ATP6V0C,ATP6V1D,BLVRB,BUD23,CAT,CCND1,CLDN12,CREBL2,CYP2J2,DIS3,E2F4,EIF1AD,FTH1,GRWD1,GSN,GSTA5,GTF2H1,HBEGF,HTRA2,IVNS1ABP,KLC4,LGALS3,LRRC40,LSG1,MDFI,MGST1,Mt1,MYC,NOP16,NUTF2,PATJ,PEG10,PIK3R3,POLR3E,PRMT1,PTGDS,RRP8,RUVBL2,SIRT2,SLC22A18,TARS,TIMP3,TMA16,TRMT6,UGT1A6,VAMP5,WDR12,WDR77
HMGA1	-0.19	TR	-2.4	7.4E-04	CCND1,CITED1,COL1A1,CCN1,GSN,IER3,INSIG1,MAPK8IP1,MYC
SMAD3	0.00	TR	-2.3	4.9E-04	ANKRD1,CCND1,COL1A1,CCN2,CXCL10,FST,FSTL3,HBEGF,HMOX1,MYC,TIMP1,TIMP3
NR1I3		LDNR	2.3	1.5E-06	Gsta4,GSTA5,GSTM1,GSTM5,GSTP1,INSIG1,Mt1,MYC,PGD,TUBA4A,WISP1
**YAP1**	0.00	TR	-2.2	2.8E-03	AMOTL2,ANKRD1,CCND1,CCN2,CCN1,MSLN,MYC
NRF1	-0.10	TR	2.2	1.6E-03	ESM1,FTH1,HMOX1,Mt1,NQO1
BACH1	0.21	TR	-2.2	1.0E-05	FTH1,HMOX1,ME1,NQO1,SQSTM1
GLIS2	-0.02	TR	2.0	1.7E-04	CCND1,COL1A1,CCN2,CXCL10
CCND1	-0.29	TR	-1.9	3.0E-03	CCND1,COL1A1,CPEB1,E2F4,ERRFI1,IER3,MYC,MYLK,NOP2,NOP58,TP53INP1,TP53INP2,TRIP13
FOXO3	0.11	TR	1.9	3.6E-06	ALDH3A1,APLN,CAT,CCND1,CHCHD4,CCN2,CXCL10,CCN1,FOXO4,GSTM5,IER3,LARS,Mt1,MXD4,MYC,OPTN,RUVBL1,TP53INP1
JUND	0.03	TR	-1.7	8.1E-04	BDNF,CCND1,FTH1,HMOX1,SERPINB9,TIMP1
JUN	-0.04	TR	-1.6	5.3E-06	BDNF,CCND1,Clca3a1/Clca3a2,COL1A1,CXCL10,CYP2J2,CCN1,FTH1,GSTA5,GSTP1,HMGA1,HMOX1,LAMP2,LGALS3,LIF,MYC,NQO1,PTGDS,SERPINB9,TIMP1,TIMP3,XPO5
PGR		LDNR	-1.6	2.6E-06	CCND1,DDX21,EIF1AX,HBEGF,IER3,KLF4,KLF9,MYC,NOP16,NSUN5,P4HA2,PLK2,PPIF,PRKG2,PRRX1,STAT5A,STEAP1
**CTNNB1**	0.05	TR	-1.5	1.1E-03	ADSS,BMF,CCND1,COL1A1,CREG1,CCN2,CYB5A,CCN1,FST,FSTL3,GPR137B,LBH,LGALS3,MAP1LC3B,ME1,MYC,MYLK,PDAP1,PDE1C,PRX,SQSTM1,
MTPN	-0.04	TR	-1.4	3.3E-03	CCND1,COL1A1,COL8A1,Mt1,MYC
STAT3	0.13	TR	-1.3	1.8E-03	BMF,BOC,CCND1,CCR1,CD9,COL1A1,CCN2,CXCL10,FST,HMOX1,KLF4,LIF,Mt1,MYC,PEG10,RALGDS,SERPINB9,TIMP1,WARS
KEAP1	0.03	TR	-1.2	1.7E-03	GSTP1,HMOX1,MAP1LC3B,NQO1

b

WNT3a + G007-LK / WNT3a

Upstream Regulator	Expr Log Ratio	Molecule Type	Activation z-score	p-value of overlap	Target molecules in dataset
SREBF2	-0.03	TR	2.9	5.2E-06	CYB5A,FABP7,FDFT1,HMGCS1,IDH1,IDI1,IRS2,LRP1,MSMO1,TIFA
PGR		LDNR	-2.7	6.2E-05	CCND1,DDX21,EDN1,EPAS1,F3,FAM49A,GPRC5A,IRS2,ITGA6,KRT18,MYC,P4HA2,PCDH1,PTGES,SLC7A5,ST3GAL4,UCK2
SOX2	-0.24	TR	2.7	1.7E-04	AXIN2,CCND1,CITED1,COL1A1,CST6,CCN2,DUSP4,EPHA2,FABP7,FOXF1,FST,GJA1,KRT18,PCDH18,TEAD1,TIMP3,TWIST1,WISP1
NFE2L2	-0.01	TR	2.5	5.9E-11	BDNF,COL1A1,CCN2,DCTN3,EPAS1,FTH1,FTL,Gsta4,GSTA5,GSTM1,GSTM5,GSTP1,HSPA9,IDH1,MAP1LC3B,ME1,MGST1,MGST3,MSMO1,Mt1,NQO1,OSGIN1,PGD,PPARGC1A,PRDX1,PREP,RARS,RRS1,SHMT2,SLC35B1,SQSTM1,SRXN1,Tpm1
SMAD3	-0.27	TR	-2.5	9.3E-06	ANKRD1,AXIN2,BMP2,CCND1,COL1A1,CCN2,DUSP4,EDN1,FST,FSTL3,MYC,PPARGC1A,PTHLH,SPHK1,SPP1,THBS1, TIMP3
**YAP1**	-0.04	TR	-2.4	1.5E-04	AMOTL2,ANKRD1,BMP2,CCND1,CCN2,CCN1,EDN1,HMGCS1,MSLN,MYC
MYC	-0.29	TR	-2.2	1.6E-14	ABCF1,AQP1,ATAD3A,BDNF,C1QBP,CAV1,CCND1,CD9,CITED1,CLIC4,CLUH,CNP,COL1A1,COL8A1,CST6,DDX21,DUSP4,EDN1,ENO1,EPHA2,F3,FBL,FOXF1,FTH1,GABARAP,GJA1,HAPLN1,HSPA9,HSPD1,IDH1,ITGA6,LAMP2,LIMA1,MGST3,Mt1,MTHFD1,MYBBP1A,MYC,NCL,NQO1,NUDC,PA2G4,PCDH18,PDGFRB,PREP,RARS,RHPN1,RRS1,SHMT2,SIRT2,SLC16A1,SLC3A2,SLC7A5,SPARC,SPP1,SRM,ST3GAL4,Tcf7,TEAD1,THBS1,TOB1,Tpm1,VARS,WISP1,YBX3
NOTCH1	0.05	TR	-2.2	4.6E-06	ADAM19,ANKRD1,BMP2,CCND1,COL1A1,CCN2,CCN1,EFNB2,ESM1,FABP7,IL18,ITGA6,MYC,MYLK,NOTCH3,PDGFRB, RND3,SPP1
PPARG		LDNR	2.1	1.3E-06	AGT,BACE1,CAV1,CCND1,COL1A1,CRAT,CCN2,CTPS1,EDN1,FST,GABARAPL2,HSD3B7,IDH1,IGFBP6,IRS2,KRT18,ME1,MGST1,PCTP,PLIN4,PPARGC1A,PTGES,SMO,SPP1,TLR4,VAMP5,VAMP8
ESR1	-0.11	LDNR	-1.8	2.5E-11	AATF,AGT,AQP1,AXIN2,BMP2,BYSL,CAMK1,CARD14,CAV1,CCND1,CDC42SE1,CENPF,CKB,COL8A1,CP,CYP26B1,CCN1,DDX21,DUSP4,EDN1,EFNB2,ENO1,F3,FST,GJA1,Gsta4,GSTA5,HSPD1,HTRA2,IRS2,ITGA6,JAK2,KLHL9,KPNB1,LIMA1,LOXL4,LTBP1,METTL7A,MR1,MYC,NFKBIE,NOTCH3,NQO1,NR1D2,NUP160,PCDH18,PPARGC1A,PPP5C,RIT1,RND1,RND3,SAMHD1,SERPINB9,SIPA1L1,SLC16A1,SLC3A2,SLC7A5,SNN,SPP1,SPTLC2,SQSTM1,SRM,TCIM,TNFAIP3,TOB1,UCK2
CREB1	-0.01	TR	-1.5	5.6E-05	ARFGEF3,BDNF,CCND1,CD9,CCN1,DKK2,EDN1,ERRFI1,FRMD6,GABARAP,HMGCS1,IDI1,IRS2,LITAF,MSMO1,MYC,PDE1C,PIM3,PPARGC1A,RNH1,Sik1,SLC16A1,SLC19A1,SQSTM1,SRXN1,TFAP2A,TG
NR1I3		LDNR	1.5	2.5E-06	FAM107B,Gsta4,GSTA5,GSTM1,GSTM5,GSTP1,Mt1,MYC,PGD,SLC29A1,TUBA4A,WISP1
BACH1	0.12	TR	-1.4	3.4E-05	FTH1,ME1,NQO1,SPP1,SQSTM1
HIF1A	0.02	TR	-1.4	4.6E-06	AGT,AXIN2,BACE1,BMP2,CAV1,CCND1,CCN2,CTPS1,CYB5A,CCN1,EDN1,ENO1,EPAS1,GJA1,HIST1H1C,IRS2,KRT18,MYC,P4HA2,PCDH10,SIRT2,SLC29A1,SPHK1,THBS1
ZNF217	0.02	TR	1.3	2.2E-06	CITED1,COL8A1,GPRC5A,HAPLN1,KRT18,MYC,SEC14L2,SEMA3A,SHC4,ZNF616
MTPN	0.04	TR	-1.3	5.3E-07	CCND1,COL1A1,COL8A1,CP,EDN1,Mt1,MYC,SPARC,SPP1,TUBA1A
GATA1		TR	1.2	1.1E-05	AQP1,CCND1,Clca3a1/Clca3a2,HSPA9,IL1RL1,IPO5,ITGA6,MGST2,MTHFD1,MYC,NCL,NOP2,PTPN6,SLC19A1,SLC3A2,SRM,Tcf7,TFEB,Tpm1
TAF4B	0.00	TR	-1.1	1.1E-04	CCN2,FST,ITGA6,SPP1,TNFAIP3
TP53	-0.07	TR	-1.1	3.5E-11	AATF,ACER2,AEN,AMOTL2,ANKRD1,AXIN2,BDNF,BMF,CAV1,CCND1,CENPF,CKB,CLIC4,CLUH,CNN2,COL1A1,CP,CCN2,CYB5A,CYP26B1,CCN1,DUSP4,E2F4,EDN1,EPAS1,EPHA2,ERF,F3,FBL,FDFT1,FRMD4A,FSTL3,FTH1,GJA1,GSTM1,GSTM5,GSTP1,GTF2H1,HMGCS1,HSPD1,IDH1,KCNJ2,KPNB1,KRT18,LGALS3,LIF,LIMA1,LTBP1,ME1,MGST2,MT-ND5,MYC,OSGIN1,PA2G4,PABPC4,PDGFRB,PGD,PIK3R3,PPARGC1A,PPP5C,PRRX2,PTPN6,RAD50
AHR	-0.07	LDNR	1.1	1.3E-04	ADAM19,CCND1,COL1A1,DMXL2,EDN1,ENO1,Gm21596/Hmgb1,GSTM5,ITGA6,LTBP1,Mt1,MYC,NOTCH3,NQO1,PDGFRB,SEMA3A,SPP1,THBS1,TIMP3
EGR1	0.01	TR	-0.9	2.7E-05	BACE1,CAV1,CCND1,COL1A1,DUSP4,F3,FTL,MAP1LC3B,ME1,MYC,PTGES,SPP1,THBS1,TLR4
EPAS1	0.53	TR	-0.9	3.0E-05	CAV1,CCND1,CKB,CCN2,EDN1,ENO1,GJA1,HIST1H1C,HMGCS1,IRS2,PCDH10,SLC29A1,SLC7A5,SPHK1,TNFAIP3
VHL	0.02	TR	0.9	8.4E-05	CAV1,CCND1,CCN2,EPAS1,F3,FTH1,FTL,MAF,NOC2L,PTHLH,SPARC
E2F1	0.01	TR	0.8	9.4E-05	CAV1,CCND1,CD9,CKB,CYB5A,DUSP4,E2F4,GABARAP,HSPD1,IRS2,LACTB,MAF,MTHFD1,MYC,NCL,NUDC,PA2G4,PPARGC1A,RND3,SLC3A2,THBS1,TP53INP1,UCK2,YBX3
CREM	0.21	TR	-0.8	1.7E-05	CCND1,COL8A1,DUSP4,ERRFI1,FRMD6,HMGCS1,IDI1,IRS2,MSMO1,Sik1,SLC16A1,THBS1,Tpm1
**CTNNB1**	-0.01	TR	-0.8	7.8E-06	AXIN2,BACE1,BMF,BMP2,CCND1,CNN2,COL1A1,CRAT,CCN2,CYB5A,CCN1,EDN1,FST,FSTL3,GJA1,HAPLN1,HMGCS1,ITGA6,LBH,LGALS3,MAP1LC3B,ME1,MYC,MYLK,NOTCH3,OGN,PDE1C,RAPSN,SDC2,SPP1,SQSTM1,Tcf7,TIMP3,TSC22D1,TWIST1,WISP1
TP73		TR	-0.8	1.1E-05	ADAM19,AEN,CITED1,COL1A1,CCN1,EDN1,EPHA2,FOXF1,FST,HS6ST1,MYC,PDGFRB,PIK3R3,PTGES,SMO,THBS1,TIMP3,TWIST1
NFKBIA	-0.06	TR	-0.7	1.4E-05	ANKRD1,AZGP1,BMP2,CCND1,CP,ESM1,F3,Gm21596/Hmgb1,GSTM5,IGFBP6,KRT18,LIF,LIMA1,LITAF,MR1,Mt1,MXD4,MYC,NFKBIE,OGN,TIMP3,TLR4,TNFAIP3,TWIST1

Supplementary Table 1. IPA core analysis of RNA sequencing data identifies CTNNB1 and YAP1 as upstream regulators upon G007-LK treatment in B16-F10 cells. a, IPA upstream regulator analysis of G007-LK versus vehicle control (0.01% DMSO) treatment (24 hours) displaying the identified upstream regulators with an activation z-score of >1.2 or <-1.2 and a *P* value of overlap <0.005. For **a** and **b**: CTNNB1 and YAP1 are highlighted in blue. Differentially expressed genes with a corrected *P* value of <0.1 were used in an IPA analysis for identifying upstream regulator components. Expression log ratio: Log2-value TPMs for transcription of the indicated upstream regulator. A low or high value indicates transcriptional difference of the upstream regulator itself. Molecule type: Depicts the biological function of the upstream regulator, TR = transcriptional regulator and LDNR = ligand-dependent nuclear receptor. Target molecule in dataset: Lists all differently expressed genes in the dataset that are linked to the upstream regulator. **b**, IPA upstream regulator analysis of G007-LK + WNT3a versus WNT3a treatment (24 hours) displaying the identified upstream regulators with an activation z-score >0.7 or <-0.7and *P* value of overlap <0.0002.

Upstream regulators for YAP^{high} versus YAP^{low} in untreated samples

upstream regulator	expression log ratio	molecule type	activation z-score	p-value of overlap	target molecules in dataset
**MITF**	-2.8	transcription regulator	-7.1	1.4E-49	ACP5,ASAH1,ATP1A1,BEST1,CAPN3,CDK5R1,CHKA,CXCL8,DCT,DSTYK,EDNRB,ESRP1,FRMD4B,GM2A,GNPTAB,GPM6B,GPNMB,GPR137B,GREB1,GZMB,INPP4B,IRF4,ITPKB,IVNS1ABP,KAZN,LGALS3,LYST,MC1R,MICAL1,MITF,MLANA,MMP14,OSTM1,PHACTR1,PIR,PMEL,QDPR,RHOQ,RRAGD,SCARB1,SEMA6A,SHTN1,SLC45A2,SLC7A8,SORT1,SOX13,SOX6,ST3GAL6,STX7,STXBP1,TBC1D16,TMCC2,TMEM251,TNFRSF14,TRPM1,TYR,UBL3,USP48,ZFYVE16
**NFATC2**	-1.7	transcription regulator	2.8	1.0E-05	ACP5,CXCL3,GPNMB,INHBA,IRF1,IRF4,MITF,MLANA,NR4A1,OASL,PMEL,STAT5A,TCF4
**SREBF1**	0.13	transcription regulator	-2.3	2.3E-05	ACADS,ADGRG1,ATOX1,BEST1,BHLHE41,FDFT1,GPNMB,HSPA1A/HSPA1B,IL1A,LGALS3,SC5D,SCARB1,SLC22A4,STX1A,STXBP1,TRIM63
**TP53**	0.40	transcription regulator	2.0	5.0E-04	ALDH4A1,AMOTL2,AREG,ATP1A1,BDNF,BHLHE41,CLU,CNN2,CTNNB1,CTSF,CXCL8,CYR61,EPHA2,FAT2,FDFT1,FERMT2,FGF2,FSTL3,FUBP1,GDA,GSN,HMGA1,HSPA1A/HSPA1B,IL1A,INHBA,KAT2B,LASP1,LGALS3,MAD1L1,MCL1,MYO10,NCOR2,NDRG2,NKD1,PARD6G,PMAIP1,PODXL,SCPEP1,SEMA6A,SFN,SLC25A13,SMURF2,TCF4,TIMP2,TUBB4A,UBL3,USP48
**TFEB**	-0.01	transcription regulator	-2.0	4.5E-04	CTSF,HEXA,NAGLU,SCPEP1,TYR
**HOXB9**	1.28	transcription regulator	2.0	1.8E-04	AREG,CXCL8,FGF2,NRG1
**NR3C1**	0.50	ligand-dependent nuclear receptor	-1.9	6.0E-05	AMIGO2,ATP6AP1,BDNF,CLCF1,CXCL3,CXCL8,CYP27A1,CYR61,DPP7,ERRFI1,IL11,IL1A,IL1RAP,IL7R,INHBA,IRF1,LYST,MAP3K14,MCL1,MMP8,OASL,RBMS2,SCARB1,SFTPC,STAT5A,TNFRSF12A,TRAF5,ZNF704
**RUNX3**	-0.76	transcription regulator	-1.9	5.1E-05	COL12A1,CRIM1,CYR61,DIP2C,EXT1,FGF2,FZD2,NUAK1,SGCD,TPM2
**SNAI1**	-0.80	transcription regulator	1.2	6.8E-06	AXL,CXCL8,CYR61,ESRP1,FLNB,GSN,LASP1,MMP14,PARVB,PEBP1,TPM1
**CTNNB1**	-0.72	transcription regulator	-1.2	2.0E-05	ADRA2C,ALDH1A1,APOD,CLU,CNN2,CTNNB1,CTSF,CXCL8,CYR61,DCT,FSTL3,GJB1,GPR137B,HMG20B,ID4,IRF4,LAMC2,LGALS3,MCL1,MITF,MMP14,NDRG2,NKD1,NR4A1,NRG1,OSBPL1A,PKP3,SFN,STAT5A,STXBP1,TCF4,TNIK
**MEOX2**	-	transcription regulator	-1.1	6.9E-06	CXCL3,CXCL8,FGF2,ID4,MMP14,PTX3,VEGFC
**SOX10**	-4.2	transcription regulator	-1.1	2.1E-04	EDNRB,GDA,GZMB,MITF,PLP1
**WT1**	1.3	transcription regulator	1.0	4.8E-04	AREG,CTNNB1,FDFT1,GSN,IL11,IL1RAP,LGALS3,MCL1,MMP14,PIR,PODXL,SMAD3,ZNF280B
**SOX2**	-0.80	transcription regulator	-1.0	3.8E-04	ABCC3,ALDH1A1,CITED1,CTNNB1,EPHA2,GAB2,GATA4,GREB1,INHBA,PARD6G,SALL4,SOX13,SOX6,TEAD3,TXNRD1,VEGFC
**NFKBIA**	0.68	transcription regulator	0.9	6.7E-07	AXL,CLU,CTNNB1,CTSF,CXCL3,CXCL8,FGF2,GM2A,GPR176,GZMB,HMGA1,IGFBP6,IL11,IL1A,IL7R,IRF1,MMP14,NAGLU,NDRG2,NR4A1,PTX3,SORL1,SPTBN1,TIMP2,VEGFC
**MYCN**	-	transcription regulator	-0.9	8.1E-04	ABCC3,ABCD1,ALDH1A1,AMOTL2,BASP1,CLU,CRIM1,GATA4,HMGA1,INHBA,MXI1,PMAIP1,TIMP2,TPM1
**SP1**	0.17	transcription regulator	-0.9	3.1E-04	BACE2,BDNF,CXCL3,CXCL8,EDA,FGF2,HMGA1,ID4,IL1A,IRF1,IRF4,MCL1,MECP2,MMP14,PDGFC,PMAIP1,ROBO4,SCARB1,SLC22A4,SMAD3,STX1A,TIMP2,TXNRD1
**JUN**	1.0	transcription regulator	0.8	3.2E-04	ACP5,AXL,BDNF,CDK5R1,CHL1,CLU,CXCL3,CXCL8,CYR61,FGF2,GREB1,HMGA1,IGFBP6,IL1A,IL7R,LGALS3,MMP8,MXI1,NR4A1,PTX3
**TP63**	-	transcription regulator	-0.8	8.8E-04	AREG,AXL,CTNNB1,CXCL8,CYR61,EPHA2,FUBP1,IGFBP6,IL1RAP,INHBA,MLPH,PMAIP1,SFN,SMURF2,STX1A,TPM1,USP48
**ESR1**	-	ligand-dependent nuclear receptor	-0.6	7.6E-04	ABCC3,AREG,ARSG,CDK5R1,CERS1,COL13A1,CRIM1,CTNNB1,CXCL3,CXCL8,EFNB2,FARP2,FZD2,GJB1,GM2A,GREB1,GSN,IFI44L,IL1A,IRF1,IRF4,MAP3K14,MC1R,PMAIP1,PTX3,RENBP,RHOQ,SCARB1,SCUBE2,SLC1A4,SLC6A8,SLC7A8,SMAD3,SMURF2,SPTBN1,STAT5A,TIMP2,TPM1,TRAK1,UBL3,VEGFC
**STAT6**	0.16	transcription regulator	-0.5	7.0E-04	ACP5,AREG,DDAH1,EXT1,FNIP2,GPM6B,IL1A,IRF1,IRF4,LDLRAD3,MMP14,PDGFC,SOX13,TNIK
**ATF4**	0.18	transcription regulator	0.5	4.7E-04	AREG,CTNNB1,CYP27A1,LGALS3,MCL1,PMAIP1,PTX3,SLC1A4,SLC6A9,TNFRSF12A

Supplementary Table 2 IPA core analysis of RNA sequencing data identifies MITF as the top upstream regulator separating the baseline YAP^{high} versus the YAP^{low} groups. Differentially expressed genes, when comparing YAP^{high} versus YAP^{low} cell lines (see Fig. 6b), with a corrected *P* value of <0.05 were used in an IPA analysis for identifying upstream regulator components. The table is displaying the identified upstream regulators with an activation z-score >0.5 or <-0.5 and *P* value of overlap <0.0005. Expression log ratio: Log2-value YAP^{high} versus YAP^{low} TPMs for transcription of the indicated upstream regulator. A low or high value indicates transcriptional difference of the upstream regulator itself. Molecule type: Depicts the biological function of the upstream regulator. Target molecule in dataset: Lists all differently expressed genes in the dataset that are linked to the upstream regulator. MITF was identified as the top upstream regulator separating the baseline YAP^{high} versus the YAP^{low} groups.

upstream regulator	expression log ratio	molecule type	activation z-score	p-value of overlap	target molecules in dataset
**TCF7L2**	0.061	transcription regulator	-2.6	6.6E-03	ANKRD13A,DOCK9,EPAS1,ERBIN,GCLM,NPC1,SNAI2,STK39
**KLF3**	-0.12	transcription regulator	2.3	2.0E-03	DYRK3,EXTL1,HLCS,HPS1,MSN,OGFRL1,TES,TMOD3,UGCG
**PGR**	1.46	ligand-dependent nuclear receptor	-1.5	5.0E-04	BCL2L1,CD36,DDX21,EPAS1,GPRC5A,NPC1,SNAPC1,TNC,UGCG
**AR**	-0.17	ligand-dependent nuclear receptor	-1.4	2.4E-03	BCL2L1,CAV1,CAV2,DSG2,EPAS1,MEF2C,NPC1,PROS1,SNAI2,STK39,TRIM25
**GLI1**	0.41	transcription regulator	-1.3	1.5E-03	BCL2L1,CAV1,MEF2C,NR2F2,PRKCE,PVR,TNC
**ESR1**	0.69	ligand-dependent nuclear receptor	-1.1	3.7E-07	BCL2L1,CAV1,CAV2,DAB2,DDB2,DDX21,DLG1,DSG2,EEA1,EXOC5,FOSL2,GNAQ,GNG12,HPS1,HTRA1,MAP3K20,NQO1,PDLIM5,PROS1,PVR,RGS3,SEC14L1,SLC7A1,SNAI2,SSR3,TACC1,TMOD3,TNFAIP3,TRIO,WASF2
**NFE2L2**	0.051	transcription regulator	-1.0	6.7E-04	BCL2L1,CD36,CTSK,EPAS1,GCLM,MEF2C,NFE2L1,NQO1,PTPN1,SNAI2,TXNRD1
**ERG**	-2.4	transcription regulator	-1.0	1.4E-03	CLIP1,ETS1,MAP3K5,NR4A2,RGS3,TACC1,WDR91

Supplementary Table 3 Tankyrase inhibition induces a transcriptional program subdividing the YAP^{low}*MITF*^{high}*MITF*^{increased} and YAP^{high}*MITF*^{low}*MITF*^{decreased} groups. IPA core analysis identifies *TCF7L2* (TCF4)(based on z-score) and *ESR1* (based on *P* value of overlap) as the top upstream regulator separating the in YAP^{low}*MITF*^{high}*MITF*^{increased} and YAP^{high}*MITF*^{low}*MITF*^{decreased} subgroups (see Supplementary Figure 26). Differentially expressed genes with an adjusted *P* value of <0.1 were used in an analysis for identifying upstream regulator components. The table is displaying the identified upstream regulators with an activation z-score >1 or <-1 and *P* value of overlap <0.01. Expression log ratio: Log2 values (counts) for YAP^{low}*MITF*^{high}*MITF*^{increased} versus YAP^{high}*MITF*^{low}*MITF*^{decreased} indicating transcription of the indicated upstream regulator. A low or high value indicates transcriptional difference of the upstream regulator itself. Molecule type: Depicts the biological function of the upstream regulator. Target molecule in dataset: Lists all differently expressed genes in the dataset that are linked to the upstream regulator.
