# Supplementary material for: Tankyrase inhibition sensitizes melanoma to PD-1 immune checkpoint blockade in syngeneic mouse models
Source: Commun Biol. 2020 Apr 24;3:196. doi: 10.1038/s42003-020-0916-2 (PMC7181813; doi:10.1038/s42003-020-0916-2)
Supplement: Supplementary file 2 — Description of Additional Supplementary Files [file 42003_2020_916_MOESM2_ESM.pdf]

Description of Additional Supplementary Files

File Name: **Supplementary Data 1**
Description: The source data underlying plots of Figs. 1-6
