# Supplementary material for: Tankyrase inhibition sensitizes melanoma to PD-1 immune checkpoint blockade in syngeneic mouse models
Source: Commun Biol. 2020 Apr 24;3:196. doi: 10.1038/s42003-020-0916-2 (PMC7181813; doi:10.1038/s42003-020-0916-2)
Supplement: Supplementary file 4 — Reporting Summary [file 42003_2020_916_MOESM4_ESM.pdf]

nature research

Corresponding author(s): Jo Waaler

Last updated by author(s): 11.03.2020

Reporting Summary

Nature Research wishes to improve the reproducibility of the work that we publish. This form provides structure for consistency and transparency in reporting. For further information on Nature Research policies, see Authors & Referees and the Editorial Policy Checklist.

Statistics

For all statistical analyses, confirm that the following items are present in the figure legend, table legend, main text, or Methods section.

n/a	Confirmed	
☐	☒	The exact sample size (*n*) for each experimental group/condition, given as a discrete number and unit of measurement
☐	☒	A statement on whether measurements were taken from distinct samples or whether the same sample was measured repeatedly
☐	☒	The statistical test(s) used AND whether they are one- or two-sided<br>*Only common tests should be described solely by name; describe more complex techniques in the Methods section.*
☐	☒	A description of all covariates tested
☐	☒	A description of any assumptions or corrections, such as tests of normality and adjustment for multiple comparisons
☐	☒	A full description of the statistical parameters including central tendency (e.g. means) or other basic estimates (e.g. regression coefficient) AND variation (e.g. standard deviation) or associated estimates of uncertainty (e.g. confidence intervals)
☐	☒	For null hypothesis testing, the test statistic (e.g. *F*, *t*, *r*) with confidence intervals, effect sizes, degrees of freedom and *P* value noted<br>*Give P values as exact values whenever suitable.*
☒	☐	For Bayesian analysis, information on the choice of priors and Markov chain Monte Carlo settings
☒	☐	For hierarchical and complex designs, identification of the appropriate level for tests and full reporting of outcomes
☒	☐	Estimates of effect sizes (e.g. Cohen's *d*, Pearson's *r*), indicating how they were calculated

Our web collection on statistics for biologists contains articles on many of the points above.

Software and code

Policy information about availability of computer code

Data collection

For DNA sequencing: Base calling, demultiplexing and quality filtering was performed using Illumina's software packages SCS2.8/RTA1.8 and Off-line Basecaller-v1.8. For RNA sequencing: Illumina`s SCS v1.2/RTA v2.1 for the NextSeq500 and bcl2fastq v2 for base calling.

Data analysis

For RNA sequencing
Transcripts were quantified with kallisto (v0.44) using ensembl transcriptome release 91 for human (GRCh38) and release 92 for mouse (GRCm38). Ensemble BioMart was used to map human orthologs in mouse60. Differentially expressed genes (DEGs) were identified with sleuth (v0.29), limma (3.34.9) (did not result in any comparisons with adjusted P values < 0.05) and DESeq264 in the R programming environment (The R Project for Statistical Computing). The R-package NMF (0.23.6) was used to make hierarchical clusters with TPM values as input. For detection of probable driver mutations, the RNAseq data was aligned with HISAT2 (v2.1.0) before VarDict (v1.2) restricted to SNVs reported more than once in COSMIC (v82) was applied. Due to the variable coverage in RNAseq data, additional SNVs found in an external unpublished gene panel sequencing experiment for the same cell lines, were included. Variants found in both data sets were annotated using ANNOVAR (2017-07-17). The data analysis was performed by the Bioinformatics Core Facility (Oslo University Hospital, Norway). Parts of the analysis are not reproducible from the github repository due to restriction regarding identifiable human data and use of commercial software. Expression data, including log2-fold change (melanoma samples), log2-fold change and p-values (B16-F10 samples), were uploaded into Ingenuity Pathway Analysis (IPA) version 01-10 (Qiagen). The expression data, with a cutoff of log2fold +/- 0.3, was analyzed using the core analysis function with the Ingenuity Knowledge Base (genes only) reference set and direct relationships, with no filters set for node types, data sources, confidence, species, tissues & cell lines and mutations.

1 Bray, N. L., Pimentel, H., Melsted, P. & Pachter, L. Near-optimal probabilistic RNA-seq quantification. Nat Biotechnol 34, 525-527, doi:10.1038/nbt.3519 (2016).
2 Kinsella, R. J. et al. Ensembl BioMarts: a hub for data retrieval across taxonomic space. Database (Oxford) 2011, bar030, doi:10.1093/database/bar030 (2011).
3 Pimentel, H., Bray, N. L., Puente, S., Melsted, P. & Pachter, L. Differential analysis of RNA-seq incorporating quantification uncertainty. Nat Methods 14, 687-690, doi:10.1038/nmeth.4324 (2017).
4 Gaujoux, R. & Seoighe, C. A flexible R package for nonnegative matrix factorization. BMC Bioinformatics 11, 367, doi:10.1186/1471-2105-11-367 (2010).

5 Kim, D., Langmead, B. & Salzberg, S. L. HISAT: a fast spliced aligner with low memory requirements. Nat Methods 12, 357-360, doi:10.1038/nmeth.3317 (2015).
6 Lai, Z. et al. VarDict: a novel and versatile variant caller for next-generation sequencing in cancer research. Nucleic Acids Research 44, e108-e108, doi:10.1093/nar/gkw227 (2016).
7 Forbes, S. A. et al. COSMIC: somatic cancer genetics at high-resolution. Nucleic Acids Research 45, D777-D783, doi:10.1093/nar/gkw1121 (2017).
8 Wang, K., Li, M. & Hakonarson, H. ANNOVAR: functional annotation of genetic variants from high-throughput sequencing data. Nucleic Acids Research 38, e164-e164, doi:10.1093/nar/gkq603 (2010).

For manuscripts utilizing custom algorithms or software that are central to the research but not yet described in published literature, software must be made available to editors/reviewers. We strongly encourage code deposition in a community repository (e.g. GitHub). See the Nature Research guidelines for submitting code & software for further information.

Data

Policy information about availability of data

All manuscripts must include a data availability statement. This statement should provide the following information, where applicable:
- Accession codes, unique identifiers, or web links for publicly available datasets
- A list of figures that have associated raw data
- A description of any restrictions on data availability

The authors declare that all data supporting the findings of this study are available within the paper and its supplementary information files.

Field-specific reporting

Please select the one below that is the best fit for your research. If you are not sure, read the appropriate sections before making your selection.

☒ Life sciences ☐ Behavioural & social sciences ☐ Ecological, evolutionary & environmental sciences

For a reference copy of the document with all sections, see nature.com/documents/nr-reporting-summary-flat.pdf

Life sciences study design

All studies must disclose on these points even when the disclosure is negative.

Sample size	No sample size calculation was performed. Sample sizes for both in vivo and in vitro experiments were determined based on experiment experience, pilots and preliminary experiments as well as what was reported in the literature. Samples sizes for each experiment and numbers of independent repeats are indicated in figure legends.
Data exclusions	For in vivo experiments, mice were occasionally euthanized for ethical reasons before experiment termination, due to skin ulcerations in the tumor area or excessive tumor size. Single outlier detections were identified by Dixon's and/or Grubb's tests (threshold, $P < 0.05$).
Replication	All in vivo experiments included contain ≥ 8 independent biological replicates, except for Fig. 4a,b (≥ 3) and Fig. 5e (≥ 3). For all in vitro assays, all attempts at replication were successful through repeated experiments (two or more replications).
Randomization	For in vivo experiments, randomization of mice to create treatment groups with ~equal tumor sizes was performed by random number generation within individual blocks (MS-Excel 2016).
Blinding	Investigators (CRO, Proqinase) were not blinded during animal experiments as all treatment groups were clearly labeled with corresponding treatments or vehicle.

Reporting for specific materials, systems and methods

We require information from authors about some types of materials, experimental systems and methods used in many studies. Here, indicate whether each material, system or method listed is relevant to your study. If you are not sure if a list item applies to your research, read the appropriate section before selecting a response.

Materials & experimental systems

n/a	Involved in the study	
☐	☒	Antibodies
☐	☒	Eukaryotic cell lines
☒	☐	Palaeontology
☐	☒	Animals and other organisms
☒	☐	Human research participants
☒	☐	Clinical data

Methods

n/a	Involved in the study	
☒	☐	ChIP-seq
☐	☒	Flow cytometry
☒	☐	MRI-based neuroimaging

Antibodies

Antibodies used

Western blot:
Primary antibodies: Tankyrase-1/2 (TNKS1/2, H-350, sc-8337, Santa Cruz Biotechnology), AXIN1 (C7B12, 3323, Cell Signaling Technology), non-phospho (active) β-catenin (D13A1, 8814, Cell Signaling Technology), total β-catenin (610153, BD Transduction Laboratories™), YAP (sc-101199, Santa Cruz Biotechnology), TAZ (HPA007415, Sigma Aldrich), AMOT1 (sc-166924, Santa Cruz Biotechnology), AMOTL1 (PA5-42267, Thermo Fisher Scientific), AMOTL2 (PA5-78770, Thermo Fisher Scientific), GSK3β (12456, Cell Signaling Technology), phospho-GSK3β (Ser9) (9323, Cell Signaling Technology). Secondary antibodies: GAPDH (sc-32233, Santa Cruz Biotechnology), β-Tubulin III (T2200, Sigma Aldrich), actin (A2066, Sigma Aldrich) and lamin B1 (ab16048, Abcam).

Cell culture immunofluorescent staining:
The following primary antibodies were used: β-catenin (610153, 1:500, BD Biosciences), Tankyrase-1/2 (H-350, sc-8337, 1:50, Santa Cruz Biotechnology [for SIM imaging]), Tankyrase-1/2 (E10, sc-365897, 1:50, Santa Cruz Biotechnology [for confocal imaging]), YAP (sc-101199, 1:100, Santa Cruz Biotechnology), AMOTL1 (PA5-42267, 1:50, Thermo Fisher Scientific) and AMOTL2 (PA5-78770, 1:50, Thermo Fisher Scientific). Secondary antibodies used (both from Thermo Fisher Scientific, 1:500): Anti-rabbit IgG Alexa488 (A-21206) and anti-Mouse IgG Alexa594 (A-11005).

In vivo checkpoint inhibition:
Anti-PD-1 (RMP1-14, BE0146, batch 614616A2, Bio X Cell) and anti-PD-L1 (10F.9G2, batch 6154598816S1, Bio X Cell).

Tumor immunostaining:
Primary antibody F4/80 (clone CI:A3, ab6640, Abcam), secondary antibody (donkey anti rat IgG Alexa Fluor 488, 5 µg/mL, Molecular Probes). Anti-CD8 (5 µg/mL, clone 4SM15, eBioscience) and secondary antibody (donkey anti-rat IgG Alexa Fluor 488, 5 µg/mL, Thermo Fisher Scientific)

Tumor flow cytometry:
Fc block (anti-mouse CD16/CD32, 1:50, 14-0161-85, clone 93, eBioscience). For T cells, the following antibodies against murine targets were used: CD45 (CD45-PacBlue [30-F11], 48-0451-82, eBioscience), CD3 (CD3-Violet 605 [17A2], 100237, BioLegend), CD4 (CD4-APC-Cy7 [GK1.5], 47-0041-82, eBioscience), CD8a (CD8-PerCP [53-6.7], 553036, BD Pharmigen), CD25 (CD25-APC [PC61.5], 17-0251-82, eBioscience) and CD44 (CD44-FITC [IM7], 11-0441-82, eBioscience). For myeloid cells, the following antibodies against murine targets were used: CD45 (CD45-PacBlue [30-F11], 48-0451-82, eBioscience), CD11b (CD11b-FITC [M1/70], 11-0112-82, eBioscience), CD11c (CD11c-APC-Cy7 [N418], 47-0114-80, eBioscience), Ly6G (Ly6G-APC [RB6-8C5], 17-5931-81, eBioscience), Ly6C (Ly6C-PE [HK1.4], 12-5932-82, eBioscience) and CD103 (CD103 [Integrin alpha E], PerCP-Cy5.5 [2E7], 121415, BioLegend). And anti-FoxP3 antibody (FoxP3-PE [FJK-16s], 12-5773-82, eBioscience).

In vivo depletion/neutralization:
Anti-IgG1 (BE0088, Bio X Cell, isotype control for INFγ), anti-IgG2 (BE0090, Bio X Cell, isotype control for anti-CD8α), anti-CD8α (BE0061, Bio X Cell) and anti-INFγ (BE0055, Bio X Cell). CD8 T cell flow cytometry: Fc block (anti-mouse CD16/CD32, 1:50, 14-0161-85, clone 93, eBioscience), BD Horizon™ BUV395 Rat Anti-Mouse CD45 (565967), BV786 Hamster Anti-Mouse CD3e (564379) and BD Horizon™ APC-R700 Rat Anti-Mouse CD8a (564983) (all from BD Biosciences).

Multiplex and ELISA immunoassays: Bio-Plex Pro Mouse Chemokine Panel 33-plex (12002231, Bio-Rad). ELISA: Mouse CCL4/ MIP-1 β Quantikine ELISA Kit (MMB00, R&D Systems)

In vitro T cell assays.
For polyclonal T cell activation, immobilized CD3 (0.5 µg/ml)/CD28 (5 µg/ml) antibodies (clones 145-2c11 and 37.51, BioXCell) were used. For flow cytometric analysis of surface markers the following antibodies were used: Anti-CD62L (1705-09L, Southern biotech), anti-CD69 (1715-02, Southern biotech), anti-CD8a (553036, BD Biosciences), anti-CD25 (17-0251-82, eBioscience), anti-CD44 (103049, Biolegend) and anti-CD3e (35-0031, TONBO Biosciences). For intracellular staining, anti-granzyme B (12-8898-82, eBioscience) was used. All antibodies were used at a working concentration of 2 µg/ml.

Validation

All antibodies were validated by manufacturers as noted on data sheets. The antibodies are commonly used, used in independent publications and catalogs numbers can be used to look up additional validation experiments.

Eukaryotic cell lines

Policy information about cell lines

Cell line source(s)

American Type Culture Collection (ATCC): Murine B16-F10 (ATCC® CRL-6475™), HEK293 cells (ATCC® CRL-1573™) Clone M-3Z1 cells (CRO, ProQinase) derived from Clone M-3 (ATCC® CCL-53.1™). The human cell lines SK-MEL-28, MeWo, and A-375 were obtained from the American Type Culture Collection (ATCC). WM35, WM115, WM1341B, WM1366, WM983B, WM451Lu, WM239A, WM266.4, WM852, WM1382, WM9, WM793B were obtained from the Wistar Institute. LOX-IMVI, FEMX-I and FEMX-V were established at the Norwegian Radium Hospital (Oslo, Norway).

Authentication

For murine cells: B16-F10 cell line authentication was performed by short tandem repeat profiling and subsequent analysis confirming C57BL/6 origin (Leibniz-Institute DSMZ).
For human cells: Cell line authentication was performed by short tandem repeat profiling and subsequent analysis at the Norwegian Radium Hospital (Oslo, Norway).

Mycoplasma contamination	The cells were routinely monitored for mycoplasma using MycoAlert Mycoplasma detection kit (Lonza). No mycoplasma was detected.
Commonly misidentified lines (See ICLAC register)	No commonly misidentified cell lines were used.

Animals and other organisms

Policy information about studies involving animals; ARRIVE guidelines recommended for reporting animal research

Laboratory animals	Spieces/strain/sex/age/provider<br>Mus musculus/C57BL/6N albino mice (C57BL/6N-TyrcBrd/BrdCrCrl)/female/5-6 weeks/Charles River.<br>Mus musculus/Rag2 mice (RAGN12F; B6.129S6-Rag2tm1Fwa N12/female/3-8 weeks/Taconic Biosciences.<br>Mus musculus/DBA/2N mice (DBA/2NCrl)/female/5-6weeks/Charles River.<br>Mus musculus/C57BL/6/female/3-8 weeks/Taconic Biosciences.<br>Mus musculus/C57BL/6-Tg(TcraTcrb)1100Mjb/J/Jackson Laboratories
Wild animals	No wild animals was used in this study.
Field-collected samples	This study does not involve samples collected from the field.
Ethics oversight	All animal experiment described were performed by ProQinase GmbH or Oslo University Hospital, following approval by local animal experiment authorities (Freiburg, Germany) or Norwegian Food Safety Authority (Norway) and in compliance with FELASA guidelines and recommendations.

Note that full information on the approval of the study protocol must also be provided in the manuscript.

Flow Cytometry

Plots

Confirm that:

☒ The axis labels state the marker and fluorochrome used (e.g. CD4-FITC).

☒ The axis scales are clearly visible. Include numbers along axes only for bottom left plot of group (a 'group' is an analysis of identical markers).

☒ All plots are contour plots with outliers or pseudocolor plots.

☒ A numerical value for number of cells or percentage (with statistics) is provided.

Methodology

Sample preparation	Tumor flow cytometry analysis:<br>Tumors were disrupted using gentleMACS Tubes (Miltenyi Biotec) containing the enzyme mix of the Tumor Dissociation Kit according to the manufacturer instructions (Miltenyi Biotech). Erythrocytes were removed with the Red Blood Cell Lysis Solution (Miltenyi Biotech). Single cell suspensions were counted, and up to 3×106 cells/well were dispensed into 96-well plates. The single cells were washed with PBS and stained for living cells (eBioscienceTM Fixable Viability Dye eFluorTM 455UV [65-0868-14, eBioscience]). After washing and centrifugation (400 x g), the samples were incubated with 50 µl/well with Fc block (anti-mouse CD16/CD32, 1:50, 14-0161-85, clone 93, eBioscience) for 30 minutes in FACS buffer (PBS with 2% FCS and 0.2% EDTA, 03690, Sigma Aldrich).<br><br>For CD8 T cell FACS analysis:<br>At experiment end, spleens from 2 animals/group were dissected and single cells were collected by squeezing the spleen through a cell strainer. Erythrocytes were removed with the Red Blood Cell Lysis Solution (Miltenyi Biotec) and 5×105 cells/well were dispensed into 96-well plates. The cells were washed with PBS and stained for living cells for 30 minutes (FVS780, BD Biosciences). After washing and centrifugation (400 x g), the samples were incubated with 50 µl/well of Fc block (anti-mouse CD16/CD32, 1:50, 14-0161-85, clone 93, eBioscience) for 15 minutes in FACS buffer (PBS with 2% FCS and 0.2% EDTA, 03690, Sigma Aldrich).<br><br>For flow cytometric analysis of surface markers on APC/ SIINFEKL or ConA-activated CD8+ T cells:<br>Wild-type C57BL/6 and C57BL/6-Tg(TcraTcrb)1100Mjb/J (OT-I) mice, harboring MHC class I (H2-Kb)-restricted and ovalbumin-specific CD8+ T cells, were obtained from Jackson Laboratories. For the T cell proliferation and cytokine release-assays, spleen and lymph nodes were harvested from OT-1 mice and CD8+ T cells were sorted by using the Miltenyi CD8a+ T Cell Isolation Kit (130-104-075). Splenocytes from wild-type C57BL/6 mice were irradiated (25 Gy) and used as antigen presenting cells. Antigen presenting cells and CD8+ T cells were mixed at a 4:1 ratio with 100,000 APCs and 25,000 T cells per well in 96-well plates. For activation of OT-1 CD8+ T cells, 4 µg/ml of synthetic SIINFEKL peptide (AnaSpec) was added. For polyclonal T cell activation, Concanavalin A (5 µg/ml, Sigma Aldrich) or immobilized CD3 (0.5 µg/ml)/CD28 (5 µg/ml) antibodies (clones 145-2c11 and 37.51, BioXCell) were used. To evaluate the effect of G007-LK on T cell proliferation and cytokine production, 1 µM G007-LK or vehicle control (0.01% DMSO) was added to all conditions. For flow cytometric analysis of surface markers on APC/ SIINFEKL or ConA-activated CD8+ T cells, the cells were pooled from 8-12 wells. For intracellular staining, the cells were incubated for 4 hours in protein-transport inhibitor before permeabilization and fixation, according to the manufactures' protocol (Fix/Perm kit with Golgistop 554715, BD Biosciences) and then stained with antibody.

Instrument	Tumor flow cytometry analysis: The samples were analyzed by flow cytometry using an LSR Fortessa (Beckton Dickenson) and the gating is shown in the Supplementary Figures. For flow cytometric analysis of surface markers on APC/ SIINFEKL or ConA-activated CD8+ T cells: The samples were analyzed using the Attune NxT flow cytometer (Thermo Fisher Scientific).
Software	Tumor flow cytometry analysis: The Kaluza Analysis flow cytometry software, Version 1.3, from Beckmann Coulter was used to analyze the flow cytometry data. For flow cytometric analysis of surface markers on APC/ SIINFEKL or ConA-activated CD8+ T cells: The samples were analyzed using Flow Jo software (BD Biosciences).
Cell population abundance	No cell sorting was performed.
Gating strategy	For all colors FMO samples were run in parallel and analyzed to differentiate negative and positive signals and set the gating. Tumor flow cytometry analysis: In the first gate, living leukocytes were classified as CD45 positive and live/dead negative cell population. The living leukocytes were then further gated for an additional marker for the major immune populations of T cells (CD3) or MDSCs (CD11b) before additional markers were used to differentiate sub-populations of each major immune population, respectively.

☒ Tick this box to confirm that a figure exemplifying the gating strategy is provided in the Supplementary Information.
